# Organic-photoredox-catalyzed three-component sulfonylative pyridylation of styrenes†

**DOI:** 10.1039/d0ra10180j

**Published:** 2020-12-22

**Authors:** Fang Wang, Jian Qin, Shengqing Zhu, Lingling Chu

**Affiliations:** State Key Laboratory for Modification of Chemical Fibers and Polymer Materials, Center for Advanced Low-Dimension Materials, College of Chemistry, Chemical Engineering and Biotechnology, Donghua University Shanghai 201620 China Lingling.chu1@dhu.edu.cn zhusq@dhu.edu.cn

## Abstract

An efficient, metal-free protocol for the three-component sulfonylative pyridylation of styrenes *via* organic-photoredox catalysis is described. This metal-free process enables the direct and selective installation of sulfonyl and heteroaryl motifs and tolerates a wide array of functional groups as well as complex molecular scaffolds, that could complement previous methods and would be of interest in pharmaceutical research.

## Abstract 2

An efficient, metal-free protocol for the three-component sulfonylative pyridylation of alkenes *via* organic-photoredox catalysis is described.

## Graphical abstract

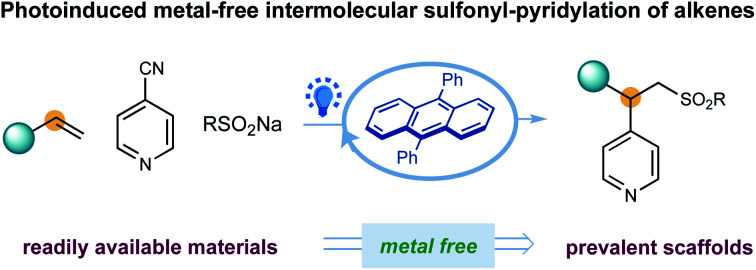

One of the most significant goals in modern chemical synthesis could be developing efficient and environment-friendly methodologies for the precise synthesis of functionalized organic molecules. Visible-light organic photoredox catalysis offers a powerful solution to this goal.^1–3^ This strategy utilizes organic chromophores to harness light energy for the catalytic generation of reactive open-shell radicals under mild and even redox-neutral conditions,^2^ offering a practical, sustainable, and pharmaceutically benign complement to the well-developed transition metal-based photoredox catalysis.^3^ Despite impressive progress in this area, there is still an increasing demand for further exploration of this metal-free, green strategy in the synthesis of complex scaffolds that would find potential applications in the pharmaceutical industry (Fig. 1).

**Fig. 1:**
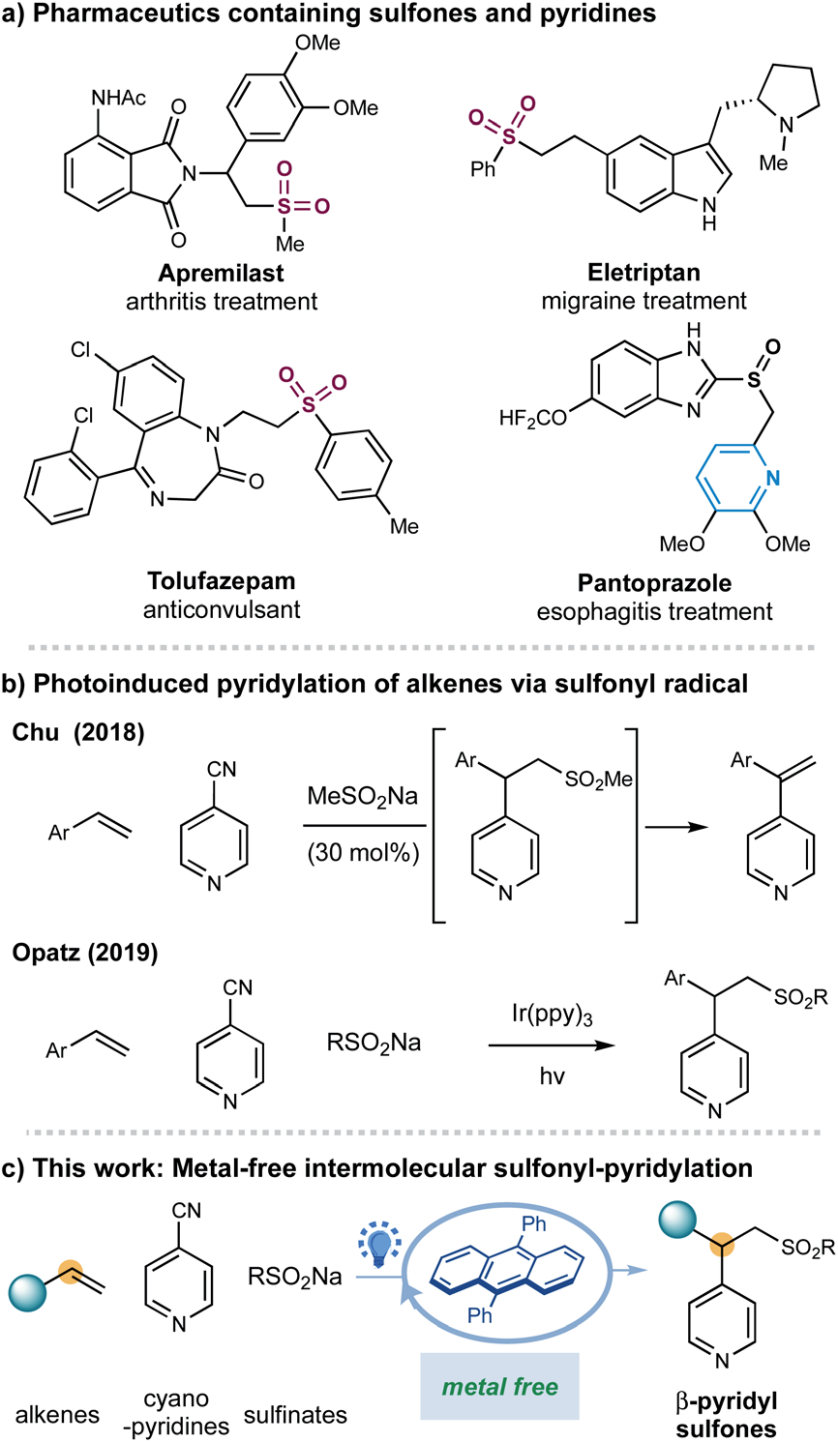
Transition-metal-free three-component sulfonyl-pyridylation of styrenes.

Sulfones are important scaffolds that display biological activities and are prevalently found in natural products and pharmaceuticals including arthritis treatment apremilast and anticonvulsant drug tolufazepam.^4^ Besides, the sulfone groups can be readily exploited as efficient coupling components^5^ and recently as diverse activators for C-radical intermediates.^6^ Regarding the synthesis of sulfones, the addition of sulfonyl radicals to alkenes represents one of the most attractive approaches for constructing functionalized alkylsulfones.^7,8^ More recently, advances in visible light-photoredox catalysis enable the generation of sulfonyl radical, promoting these transformations under milder and redox-neutral conditions.^8^ Nonetheless, rare examples of catalytic intermolecular carbosulfonylation of alkenes, that can simultaneously introduce another C–C bond thus assemble complex molecular skeletons from simple materials, have been disclosed.^8*j*,*o*,*r*,9^ Herein, we report an organic-photoredox-catalyzed three-component sulfonylative pyridylation of alkenes with sulfinate salts and pyridines under metal-free conditions.

Recently, our groups have developed several radical pyridylation of alkenes *via* a photoinduced sequential radical addition–radical coupling strategy, constructing β-functionalized pyridines with distinct selectivity.^10^ In 2018, we utilized MeSO_2_Na as an addition/elimination mediator to achieve a photoinduced branch-selective pyridylation of alkenes in the presence of Ir(ppy)_3_, where β-sulfonyl pyridines were the crucial intermediates.^11^ Considering the importance of both sulfones and pyridines,^12^ we herein further disclosed the photoinduced 9,10-diphenylanthracene (DPA)-mediated β-sulfonylative pyridylation of alkenes with sulfinates and pyridines under metal-free conditions. This metal-free protocol could complement Opatz's protocol which disclosed a similar transformation by using Ir(ppy)_3_ as the photocatalyst.^9*d*^

On the basis of our previous work, we chose 4-^*t*^Bu-styrene 1, 4-cyanopyridine 2 and sodium methanesulfinate as template substrates to evaluate organic photoredox catalysts (Table 1). Under the irradiation of blue LED light, we were pleased to find that the inexpensive organic dye 9,10-diphenylanthracene (DPA) could effectively promote the desired three-component difunctionalization reaction in the presence of NH_4_Cl, affording the β-sulfonylative pyridine product 3 in 94% yield (entry 1). Eosin Y also demonstrated relatively high efficiency (entry 2); while other organic photocatalysts showed dramatically decreased efficiency (entries 3–6). Anthracenes with 9,10-CN or -OMe substituents didn't work in this transformation (entries 5 and 6). Medium polar solvents such as acetone and MeCN were more effective than polar solvents, and the use of ethanol as co-solvent was optimal, probably due to the improved solubility of sulfonate salts in the co-solvent system (entries 7–10). The use of acidic additive such as NH_4_Cl could be able to some extent neutralize the reaction condition and was beneficial to the reaction yield (entry 11). This finding is consistent to our previous work that elimination of sulfonyl groups proceeded under basic conditions.^11^ Finally, control studies confirmed that both the light and the photocatalyst were required to this sulfonyl-pyridylation reaction (entries 12 and 13).

**Table 1:** Optimization of reaction conditions^a^

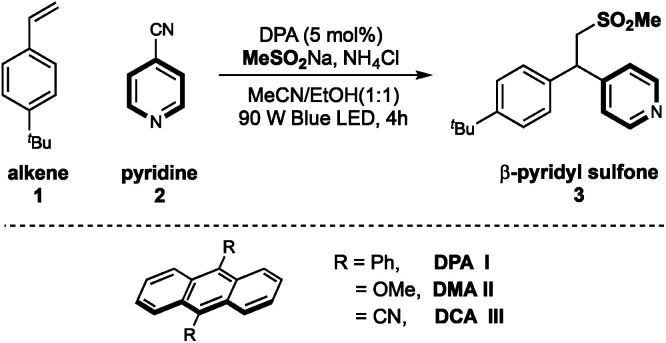		
Entry	Variations from “standard condition”	Yield of 3
1	None	94%
2	Eosin-Y instead of DPA I	82%
3	4CzIPN, instead of DPA I	45%
4	Benzophenone, instead of DPA I	8%
5	DCA II, instead of DPA I	23%
6	DMA III, instead of DPA I	5%
7	MeCN, instead of MeCN/EtOH	83%
8	Acetone, instead of MeCN/EtOH	79%
9	EtOH, instead of MeCN/EtOH	63%
10	DMSO, instead of MeCN/EtOH	0
11	W/o NH_4_Cl	78%
12	W/o DPA I	0
13	W/o light	0

a Reaction conditions: DPA I (5 mol%), styrene 1 (0.1 mmol), 4-cyanopyridine 2 (2.0 equiv.), MeSO_2_Na (1.5 equiv.), and NH_4_Cl (2.0 equiv.), MeCN/EtOH (1 : 1) [0.025 M], 90 W blue LED, r.t., 4 h. Yields were determined by ^1^H NMR using 1,3-benzodioxole as an internal standard.

With optimized conditions established, the substrate scope of this light-induced metal-free intermolecular sulfonylative pyridylation protocol was next evaluated. As shown in Scheme 1, a wide array of terminal styrenes incorporated with electron-donating and electron-withdrawing groups smoothly underwent selective cross-couplings with 4-cyanopyridine 2 and sodium methanesulfinate under the standard conditions, delivering the corresponding β-sulfonyl pyridines with high efficiency (products 3–14, 69–95% yields). This mild and metal-free conditions were well-compatible to most functional groups, including unprotected amines and iodides that are reactive in transition-metal-catalyzed cross-coupling reactions, were well compatible, delivering the final products that could be leveraged for further synthetic manipulations (products 8 and 11, 69% and 89% yields). The reaction efficiency was not impacted by sterically demanding *ortho*-substituents on styrenes, as exemplified by product 15 (92% yield). Furthermore, naphthalene- and benzofuran-derived alkenes were also competent substrates (products 16 and 17, 77% and 86% yields). Additionally, cyclic internal alkenes such as 1,2-dihydronaphthalene were suitable substrates in this reaction, forming the sulfonylative products with moderate yields and excellent regioselectivity (product 18, 79% yield). Interestingly, the reactions of 1,1-disubstituted alkenes exclusively delivered β-sulfonyl tertiary-alkyl pyridines with efficient construction of quaternary carbon centers (products 19 and 20, 98% and 73% yields). The sulfones and pyridines could also be successfully incorporated into complex drugs-derived molecules with high efficiency, further demonstrated the synthetic utility of this metal-free protocol in late-stage modifications (products 21–24, 60–97% yields). Furthermore, a scaled-up reaction was also performed using the standard and operationally simple conditions, and 89% yield of product 3 was obtained in gram scale (Scheme 1).

**Scheme 1:**
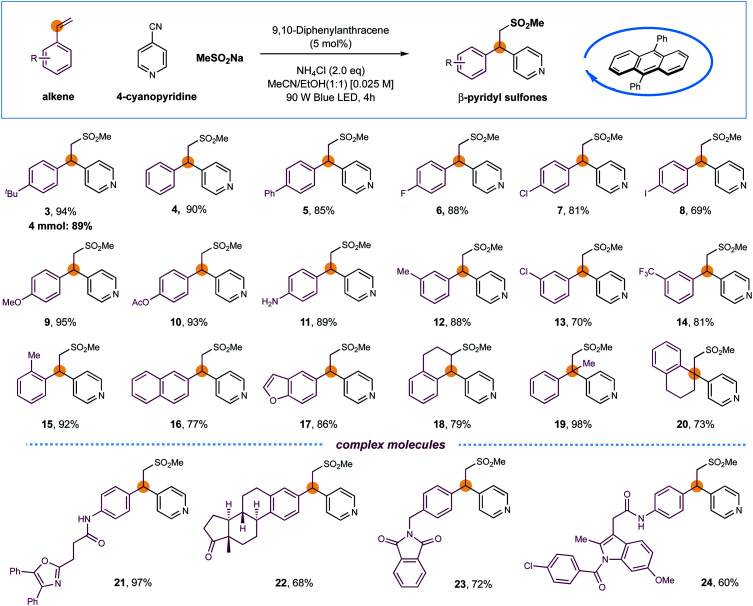
Substrate scope of styrenes. Reaction conditions: DPA I (5 mol%), styrene (0.2 mmol), 4-cyanopyridine 2 (2.0 equiv.), MeSO_2_Na (1.5 equiv.), and NH_4_Cl (2.0 equiv.), MeCN/EtOH (1 : 1) [0.025 M], 90 W blue LED, r.t., 4 h. All cited yields are isolated yields.

Next, the applicability of pyridines was investigated (Scheme 2). Cyanopyridines with substituents such as methyl, bromo and phenyl at the 2-positions were amenable to the desired couplings to introduce pyridines with excellent efficiency and regioselectivity (products 25–28, 83–90% yields). 3-Substituents (Me, Cl, CN) on pyridines were also well compatible, giving 3,4-disubstituted pyridines with excellent yields (products 29–31, 88–97% yields). 2-Cyanopyridines were also suitable, albeit with moderate efficiency (products 32 and 33, 72%, 71% yields, respectively). To our delight, the reaction of fused-heterocycles including 1-cyano-isoquinoline and unprotected azaindole nitrile, prevalent scaffolds in drugs and natural products, went very well, regioselectively generating the functionalized heteroarene products in high yields (products 34 and 35, 84% and 74% yield, respectively).

**Scheme 2:**
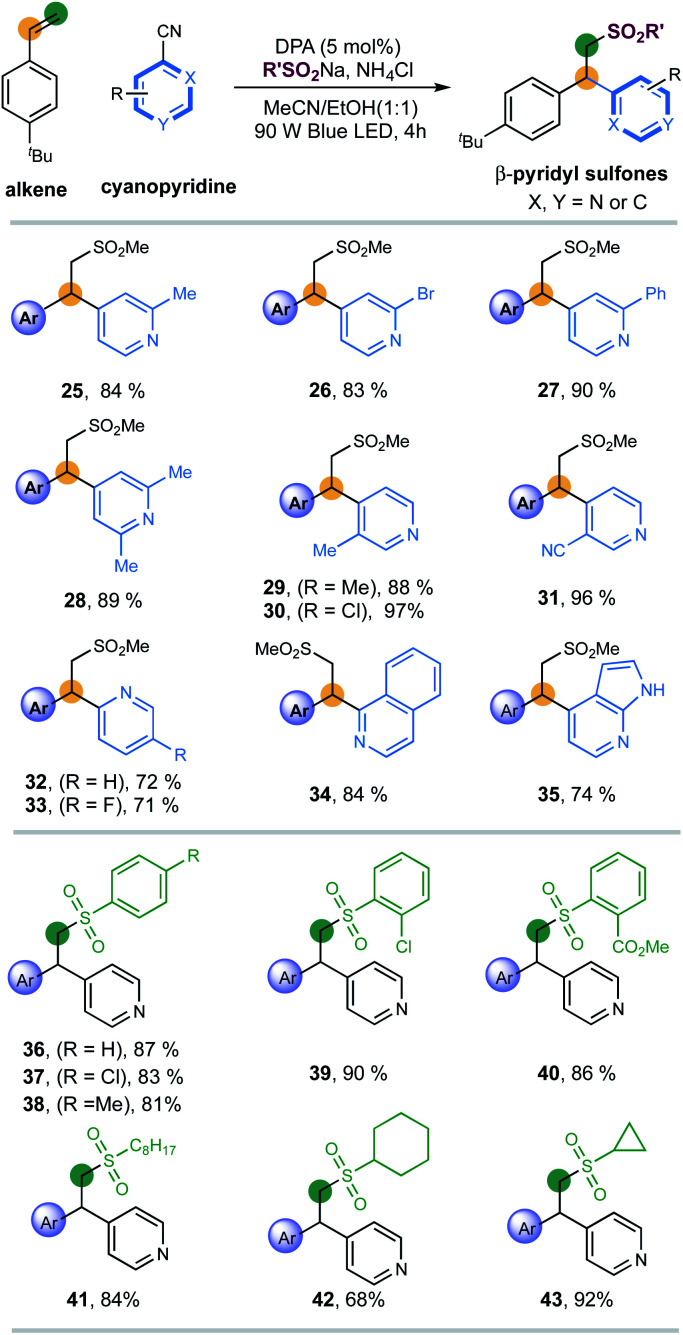
Substrate scope of cyanopyridines and sulfinates. Reaction conditions: DPA I (5 mol%), styrene 1 (0.2 mmol), heteroarenes (2.0 equiv.), RSO_2_Na (1.5 equiv.), and NH_4_Cl (2.0 equiv.), MeCN/EtOH (1 : 1) [0.025 M], 90 W blue LED, r.t., 4 h. All cited yields are isolated yields.

Finally, a series of sulfinates were investigated under the standard conditions (Scheme 2). Both aryl and alkyl sulfinates underwent the expected difunctionalization reaction with excellent levels of efficiency (products 36–43, 68–92% yields). With regards to aryl sulfinates, no obvious steric or electronic effect of substituents has been observed, suggesting the robustness of this radical protocol (products 36–40, 81–90% yields).

To probe the potential reaction pathway, we have performed several preliminary mechanistic studies. Under the standard conditions, the reaction of 4-cyanopyridine 2 and sulfinate with cyclopropyl-styrene 44, a typical radical clock agent, gave 47% yield of product 45 (Scheme 3a). We assumed that addition of sulfonyl radical to alkene followed by a ring opening gave allylic radical 45-I, which subsequently underwent an intramolecular cyclization to generate radical 45-II. A selective radical–radical coupling of 45-II with pyridine could form intermediate 45-III, that was unstable in this photoinduced system and prone to undergo oxidative aromatization to furnish the final product 45 (Scheme 3a). Additionally, Stern–Volmer fluorescence quenching experiments showed that the excited state of DPA I (*E*_1/2_ = −1.77 V *versus* SCE in acetonitrile)^13^ was only quenched by cyanopyridine 2 (*E*_1/2_ = −1.75 V *versus* SCE in acetonitrile)^14^, other than sodium sulfonate or alkene (Scheme 3b).

**Scheme 3:**
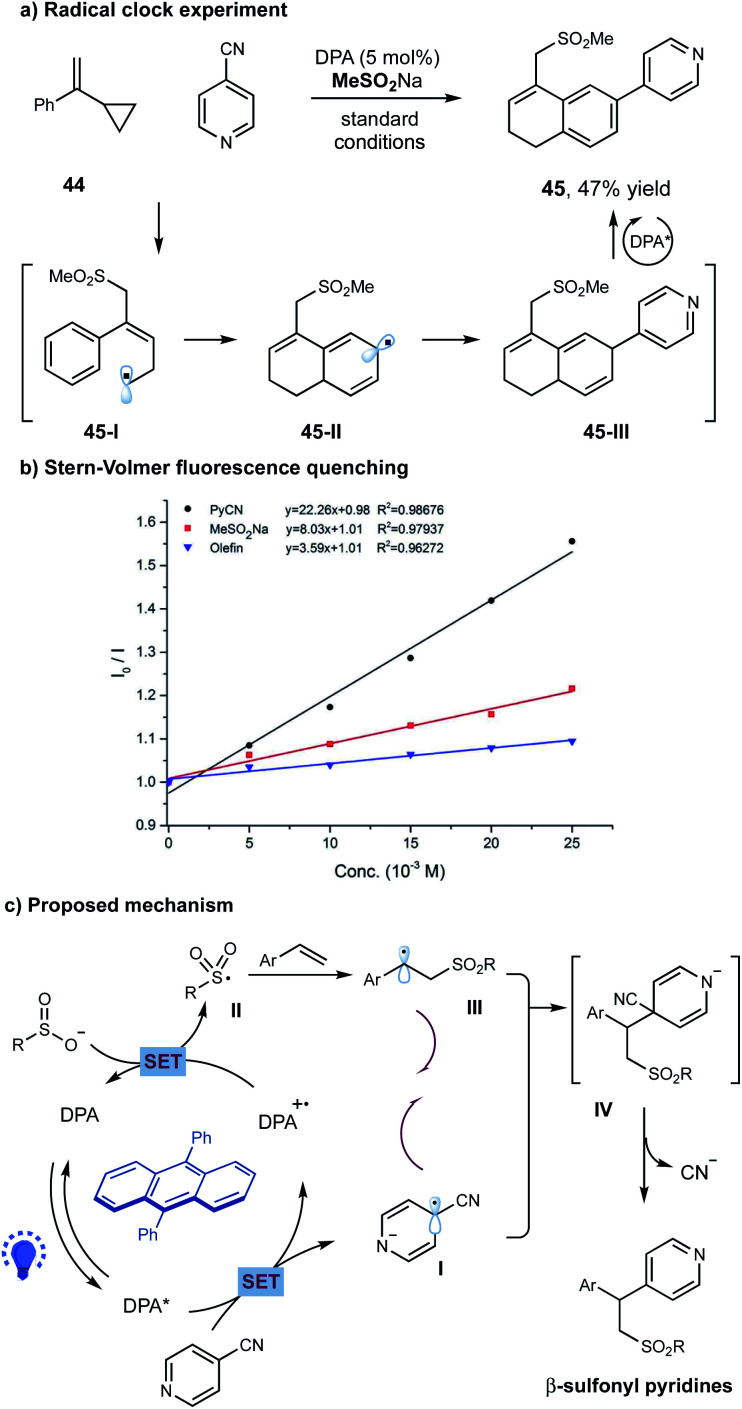
Mechanistic studies and proposed reaction pathway. (a) Radical clock reaction; (b) Stern–Volmer quenching studies; (c) proposed reaction pathway.

On the basis of these experimental results, a potential reaction pathway was depicted in Scheme 3c. Initially, a single-electron-transfer (SET) event between the photoexcited *DPA and cyanopyridine gave the pyridyl radical anion species I and DPA˙^+^. The oxidizing DPA˙^+^ species was capable to oxidize sodium sulfinate (*E*_1/2_ = 0.50 V *versus* SCE in acetonitrile)^11^, delivering the ground state DPA and the electrophilic sulfonyl radical II. Subsequently, radical II added to an alkene, giving the nucleophilic alkyl radical III. At this stage, a selective radical–radical coupling between the transient alkyl radical III and the persistent radical anion I could proceed to form the coupled intermediate IV, which then underwent a facile elimination of cyanide to furnish the final β-sulfonyl pyridine product.

## Conclusions

In conclusion, we have developed a metal-free photoinduced protocol for the catalytic three-component sulfonylative pyridylation of alkenes. This operationally simple reaction exhibits a broad tolerance of functional groups, facilitating the direct and selective incorporation of both important heteroaryl and sulfonyl groups from simple starting materials. Mechanistic studies indicated the involvement of a single-electron-transfer reduction of cyanopyridine step. Considering the valuableness of these scaffolds as well as the metal-free feature of this methodology, we expect that it would find interesting applications in pharmaceutical and agrochemical research.

## Conflicts of interest

There are no conflicts of interest to declare.

## Supplementary Material

RA-011-D0RA10180J-s001
