# Supplementary material for: Organic-photoredox-catalyzed three-component sulfonylative pyridylation of styrenes
Source: RSC Adv. 2020 Dec 22;11(1):142–6. doi: 10.1039/d0ra10180j (PMC8691066; doi:10.1039/d0ra10180j)
Supplement: RA-011-D0RA10180J-s001 [file RA-011-D0RA10180J-s001.pdf]

Electronic Supplementary Material (ESI) for RSC Advances.
This journal is © The Royal Society of Chemistry 2020

Supplementary Materials for

# Organic-Photoredox-Catalyzed Three-Component Sulfonylative Pyridylation of Styrenes

Fang Wang, Jian Qin, Shengqing Zhu, and Lingling Chu

# Table of Contents

**1. General Information** .......... S3

**2. General Procedures** .......... S4

**3. Optimization of the Reaction Conditions** .......... S6

**4. Preparation of Substrates** .......... S9

**5. Characterizations Date for Products** .......... S10

**6. Mechanistic Studies** .......... S37

**7. NMR Spectra** .......... S40

**8. References** .......... S85

## 1. General Information

**General Considerations:** Commercial reagents were purchased from Adamas, Aldrich, TCI, Energy Chemical, Bide, Leyan and J&K chemical, and were used as received. All reactions were carried out in oven-dried glassware under an atmosphere of nitrogen unless otherwise noted. Chromatographic purification of products was accomplished by flash chromatography using silica gel. Thin-layer chromatography (TLC) was performed on Silicycle 250 mm silica gel F-254 plates. $^{1}$H, $^{13}$C and $^{19}$F NMR spectra were recorded on Bruker 400 (400, 100 and 375 MHz) and Bruker 600 (600, 150 and 565 MHz), and are internally referenced to residual solvent signals (for $CDCl_3$, 7.26 and 77.0 ppm, and for DMSO-$d_6$, 2.50, 39.5 ppm). Data for $^{1}$H NMR and $^{19}$F NMR are reported as follows: chemical shift (δ ppm), multiplicity (s = singlet, d = doublet, t = triplet, m = multiplet, br = broad), integration, coupling constant (Hz). $^{13}$C spectra were reported as chemical shifts in ppm and multiplicity where appropriate. High resolution mass spectra were obtained at Shanghai Institute of Organic Chemistry mass spectrometry facilitie Photochemical experiments have been performed using 90 W Blue LED light (commercialized from WATTCAS$^{TM}$).

## 2. General Procedures

**2.1. General procedure for the sulfonylative pyridylation of alkenes:** To a flame-dried 10 mL reaction vial was charged with 9, 10-diphenylanthracene (0.01 mmol, 5 mol %), Cyanopyridine (0.4 mmol, 2.0 equiv), and Sodium methyl sulfinate (0.3 mmol, 1.5 equiv), Olefins (0.2 mmol, 1.0 equiv, if solid) and Ammonium chloride (0.4 mmol, 2.0 equiv). The vial was capped. MeCN/EtOH (1:1) [0.025 M] was added via a syringe. It was bubbled with nitrogen for 15 minutes, followed by the addition of Olefins (0.2 mmol, 1.0 equiv., if liquid). The reaction mixture was then irradiated with a 90 W Blue LED lamp for 4 h at room temperature. After reaction completed, the mixture was evaporated on rotary evaporator. Then, the residue was dilute with ethyl acetate and wash with $H_2O$. The combined organic layers were dried with $MgSO_4$, filtered, and concentrated in vacuo. The crude material was purified by flash chromatography to afford the product.

**2.2. The gram-scale synthesis of β-pyridyl sulfone 3**

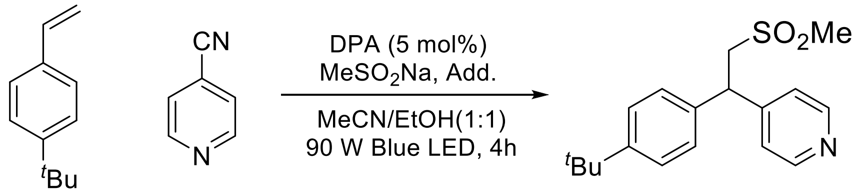


4.0 mmol

**3**, 89 % Yield

To a 250 mL oven-dried round bottom flask containing a magnetic stir bar was added 9, 10-diphenylanthracene (66.1 mg, 0.2 mmol, 5 mol %), Cyanopyridine (832 mg, 8.0 mmol, 2.0 equiv), Sodium methyl sulfinate (612 mg, 6.0 mmol, 1.5 equiv) and Ammonium chloride (428 mg, 8.0 mmol, 2.0 equiv). The vial was capped and MeCN/EtOH (80 mL/80 mL) was added via a syringe. It was bubbled with nitrogen for 30 minutes, followed by the addition of 1-(tert-butyl)-4-vinylbenzene (0.73 mL, 4.0 mmol, 1.0 equiv.). The reaction mixture was then irradiated with a 90 W Blue

LED lamp for 4 h at room temperature. After reaction completed, the mixture was evaporated on rotary evaporator. Then, the residue was dilute with ethyl acetate and wash with $H_2O$. The combined organic layers were dried with $MgSO_4$, filtered, and concentrated in vacuo. The product was isolated by flash chromatography (petroleum ether: ethyl acetate = 1: 2) as a pale-yellow oil (1.13 g, 89 %)

## 3. Optimization of the Reaction Conditions

**General procedure for the optimization studies:** To a flame-dried 10 mL reaction vial was charged with 9, 10-diphenylanthracene (0.005 mmol, 5 mol %), Cyanopyridine (0.2 mmol, 2.0 equiv), and Sodium methyl sulfinate (0.15 mmol, 1.5 equiv), Ammonium chloride (0.2 mmol, 2.0 equiv) and MeCN/EtOH (1:1) [0.025 M] was added via a syringe. It was bubbled with nitrogen for 15 minutes, followed by the addition of 1-(tert-butyl)-4-vinylbenzene (0.1 mmol, 1.0 equiv). The reaction mixture was then irradiated with a 90 W Blue LED lamp for 4 h at room temperature. The reaction mixtures were analyzed by $^1$H NMR with an internal standard.

**Table S1.Catalyst effect.**

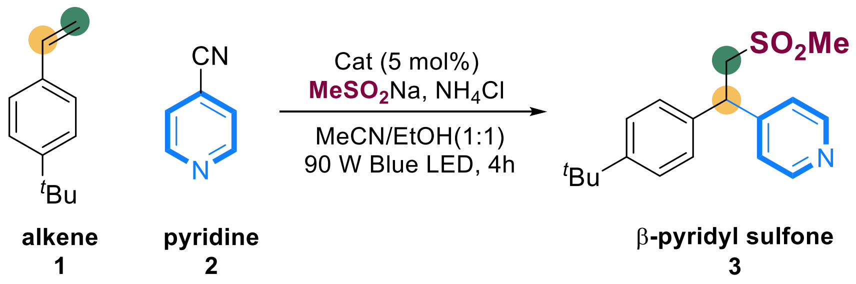


| Entry | Catalyst | Yields of 3 |
|---|---|---|
| 1 | DPA | 94% |
| 2 | Eosin-Y | 82% |
| 3 | 4CzIPN | 45% |
| 4 | DCA | 23% |
| 5 | DMA | 5% |
| 6 | Benzophenone | 8% |
| 7 | 9-Fluorenone | Tr |
| 8 | 4,4’-Biphenyldicarbonitrile | Tr |
| 9 | Anthraquinone | 43% |
| 10 | Pyrene | 67% |

**Table S2. Solvent effect**

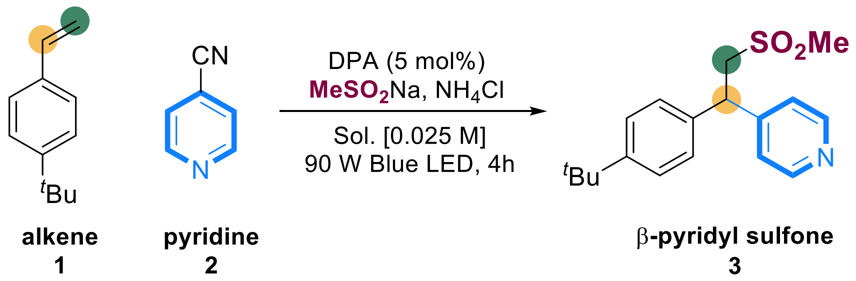


| Entry | Solvent | Yields of 3 |
|---|---|---|
| 1 | MeCN/EtOH | 94% |
| 2 | MeCN | 83% |
| 3 | Acetone | 79% |
| 4 | THF | 7% |
| 5 | DCM | <5% |
| 6 | DMSO | 0% |
| 7 | Toluene | 0% |
| 8 | DCE | 25% |
| 9 | EtOH | 63% |

**Table S3. Additive effect**

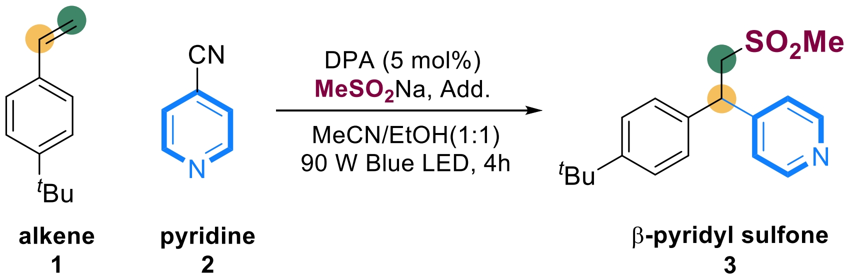


| Entry | Additive | Yields of 3 |
|---|---|---|
| 1 | $NH_4Cl$ | 94% |
| 2 | $NH_4OAc$ | 49% |
| 3 | TBAI | 51% |
| 4 | $NH_4PF_6$ | 54% |
| 5 | $NH_4HCO_3$ | 73% |
| 6 | HOAc | 53% |
| 7 | TFA | 88% |
| 8 | $AlCl_3$ | 0% |

# 4. Preparation of Substrates

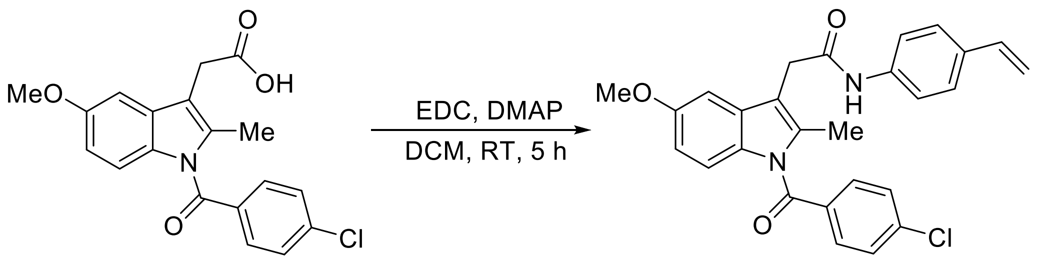


**S1**, 70 % yield

To a 250 mL oven-dried round bottom flask containing a magnetic stir bar was charged with Indometacin (3.0 mmol, 1.0 equiv), 1-(3-Dimethylaminopropyl)-3-ethylcarbodiimide hydrochloride (4.5 mmol, 1.5 equiv), and 4-Dimethylaminopyridine (0.3 mmol, 1.5 equiv). After it was evacuated and backfilled nitrogen three times, $CH_2Cl_2$ (60 mL) was added via a syringe, followed by the addition of 4-Vinylbenzenamine (3.06 mmol, 1.02 equiv.). The reaction mixture was stirred at room temperature for 5 hours and then quenched with 15 mL $H_2O$. The resulting mixture was extracted with $CH_2Cl_2$ and the combined extracts were dried with $Na_2SO_4$, filtered, concentrated in vacuo and purified by silica gel chromatography (petroleum ether: ethyl acetate = 5:1) to give target product as pale-yellow solid. (700 mg, 70%).

$^1$H NMR (400 MHz, $CDCl_3$) δ 7.59 (d, J = 8.3 Hz, 2H), 7.41 (d, J = 8.3 Hz, 2H), 7.36 – 7.19 (m, 5H), 6.87 (d, J = 1.6 Hz, 1H), 6.80 (d, J = 9.0 Hz, 1H), 6.64 (dd, J = 9.0, 1.8 Hz, 1H), 6.56 (dd, J = 17.6, 10.9 Hz, 1H), 5.58 (d, J = 17.6 Hz, 1H), 5.10 (d, J = 10.9 Hz, 1H), 3.73 (s, 5H), 2.37 (s, 3H). $^{13}$C NMR (101 MHz, $CDCl_3$) δ 168.29, 168.07, 156.37, 139.63, 136.90, 136.64, 135.97, 134.01, 133.46, 131.19, 130.92, 130.11, 129.22, 126.72, 120.02, 115.19, 113.20, 112.44, 112.29, 100.70, 55.74, 33.31, 13.32. HRMS (ESI+): calcd for $C_{27}H_{24}ClN_2O_3^+$ (M+H) 459.1470, found 459.1466.

## 5. Characterizations Date for Products

**4-(1-(4-(tert-butyl)phenyl)-2-(methylsulfonyl)ethyl)pyridine (3)**

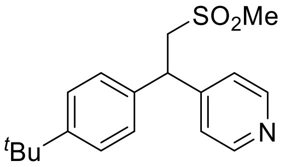


According to the general procedure, 9,10-diphenylanthracene (3.3 mg, 0.01 mmol, 5 mol%), 4-cyanopyridine (41.6 mg, 0.4 mmol, 2.0 equiv.), and Sodium methyl sulfinate (34 mg, 0.3 mmol, 1.5 equiv.), 1-(tert-butyl)-4-vinylbenzene (36.6 μL, 0.2 mmol, 1.0 equiv.) and Ammonium chloride (21.4 mg, 0.4 mmol, 2.0 equiv.), the product was isolated by flash chromatography (petroleum ether: ethyl acetate = 1: 2) as a pale-yellow oil (59.7 mg, 94%). $^1$H NMR (400 MHz, $CDCl_3$) δ 8.55 (d, J = 6.0 Hz, 2H), 7.36 (d, J = 8.3 Hz, 2H), 7.22 (m, 4H), 4.62 (t, J = 7.2 Hz, 1H), 3.75 (m, 2H), 2.42 (s, 3H), 1.27 (s, 9H). $^{13}$C NMR (101 MHz, $CDCl_3$) δ 151.17, 150.90, 150.15, 136.47, 127.42, 126.26, 122.83, 59.70, 44.95, 42.06, 34.47, 31.16. HRMS (ESI+): calcd for $C_{18}H_{24}NO_2S^+$ (M+H) 318.1522, found 318.1520.

**4-(2-(methylsulfonyl)-1-phenylethyl)pyridine (4)**

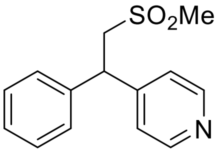


According to the general procedure, 9,10-diphenylanthracene (3.3 mg, 0.01 mmol, 5.0 mol%), 4-cyanopyridine (41.6 mg, 0.4 mmol, 2.0 equiv.), and Sodium methyl sulfinate (34.0 mg, 0.3 mmol, 1.5 equiv.), Styrene (23.0 μL, 0.2 mmol, 1.0 equiv.) and Ammonium chloride (21.4 mg, 0.4 mmol, 2.0 equiv.), the product was isolated by flash chromatography (petroleum ether: ethyl acetate = 1: 2) as a pale-yellow oil (47.0 mg, 90%). $^1$H NMR (400 MHz, $CDCl_3$) δ 8.48 (d, J = 5.6 Hz, 2H), 7.33 – 7.27 (m,

2H), 7.25 – 7.20 (m, 3H), 7.16 (d, J = 5.6 Hz, 2H), 4.64 – 4.55 (m, 1H), 3.78 – 3.62 (m, 2H), 2.37 (s, 3H). $^{13}$C NMR (101 MHz, $CDCl_3$) δ 150.57, 150.11, 139.64, 129.39, 128.06, 127.84, 122.70, 59.63, 45.35, 42.12.[1]

**4-(1-([1,1'-biphenyl]-4-yl)-2-(methylsulfonyl)ethyl)pyridine (5)**

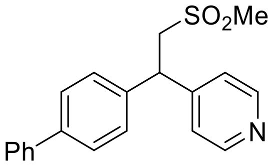


According to the general procedure, 9,10-diphenylanthracene (3.3 mg, 0.01 mmol, 5 mol%), 4-cyanopyridine (41.6 mg, 0.4 mmol, 2.0 equiv.), and Sodium methyl sulfinate (34 mg, 0.3 mmol, 1.5 equiv.), 4-vinyl-1,1'-biphenyl (36.0 mg, 0.2 mmol, 1.0 equiv.) and Ammonium chloride (21.4 mg, 0.4 mmol, 2.0 equiv.), the product was isolated by flash chromatography (petroleum ether: ethyl acetate = 1: 2) as a yellow solid (57.4 mg, 85%). $^{1}$H NMR (400 MHz, $CDCl_3$) δ 8.57 (d, J = 5.2 Hz, 2H), 7.57 (m, 4H), 7.43 (t, J = 7.6 Hz, 2H), 7.39 – 7.32 (m, 3H), 7.27 (d, J = 4.7 Hz, 2H), 4.71 (t, J = 7.1 Hz, 1H), 3.81 (m, 2H), 2.50 (s, 3H). $^{13}$C NMR (101 MHz, $CDCl_3$) δ 150.88, 149.89, 141.02, 139.85, 138.51, 128.84, 128.26, 128.01, 127.69, 126.94, 122.88, 59.58, 45.02, 42.25. HRMS (ESI+): calcd for $C_{20}H_{20}NO_2S^{+}$ (M+H) 338.1209, 338.1205.

**4-(1-(4-fluorophenyl)-2-(methylsulfonyl)ethyl)pyridine (6)**

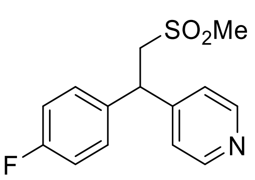


According to the general procedure, 9,10-diphenylanthracene (3.3 mg, 0.01 mmol, 5 mol%), 4-cyanopyridine (41.6 mg, 0.4 mmol, 2.0 equiv.), and Sodium methyl sulfinate (34 mg, 0.3 mmol, 1.5 equiv.), 1-fluoro-4-vinylbenzene (23.9 μL, 0.2 mmol,

1.0 equiv.) and Ammonium chloride (21.4 mg, 0.4 mmol, 2.0 equiv.), the product was isolated by flash chromatography (petroleum ether: ethyl acetate = 1: 2) as a pale-yellow oil (49.1 mg, 88%). $^1$H NMR (400 MHz, $CDCl_3$) δ 8.50 (d, J = 5.8 Hz, 2H), 7.24 – 7.18 (m, 2H), 7.15 (d, J = 5.8 Hz, 2H), 6.99 (t, J = 8.6 Hz, 2H), 4.61 (t, J = 7.2 Hz, 1H), 3.69 (d, J = 7.2 Hz, 2H), 2.45 (s, 3H). $^{19}$F NMR (377 MHz, $CDCl_3$) δ -113.42 – -113.49 (m). $^{13}$C NMR (101 MHz, $CDCl_3$) δ 162.18 (d, J = 248.1 Hz), 150.55, 150.09, 135.49 (d, J = 3.3 Hz), 129.51 (d, J = 8.1 Hz), 122.70, 116.36 (d, J = 21.7 Hz), 59.57, 44.48, 42.27. HRMS (ESI+): calcd for $C_{14}H_{15}FNO_2S^+$ (M+H) 280.0802, found 280.0800.

**4-(1-(4-chlorophenyl)-2-(methylsulfonyl)ethyl)pyridine (7)**

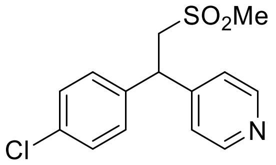


According to the general procedure, 9,10-diphenylanthracene (3.3 mg, 0.01 mmol, 5 mol%), 4-cyanopyridine (41.6 mg, 0.4 mmol, 2.0 equiv.), and Sodium methyl sulfinate (34 mg, 0.3 mmol, 1.5 equiv.), 1-chloro-4-vinylbenzene (24.0 μL, 0.2 mmol, 1.0 equiv.) and Ammonium chloride (21.4 mg, 0.4 mmol, 2.0 equiv.), the product was isolated by flash chromatography (petroleum ether: ethyl acetate = 1: 2) as a white solid (47.9 mg, 81%). $^1$H NMR (600 MHz, $CDCl_3$) δ 8.55 (d, J = 5.2 Hz, 2H), 7.33 (d, J = 8.4 Hz, 2H), 7.22 (m, 4H), 4.65 (t, J = 7.1 Hz, 1H), 3.75 (d, J = 7.1 Hz, 2H), 2.53 (s, 3H). $^{13}$C NMR (101 MHz, $CDCl_3$) δ 150.31, 150.07, 138.18, 134.06, 129.56, 129.19, 122.73, 59.34, 44.57, 42.32. HRMS (ESI+): calcd for $C_{14}H_{15}ClNO_2S^+$ (M+H) 296.0507, found 296.0505.

**4-(1-(4-iodophenyl)-2-(methylsulfonyl)ethyl)pyridine (8)**

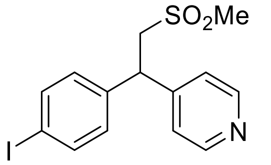


According to the general procedure, 9,10-diphenylanthracene (3.3 mg, 0.01 mmol, 5 mol%), 4-cyanopyridine (41.6 mg, 0.4 mmol, 2.0 equiv.), and Sodium methyl sulfinate (34 mg, 0.3 mmol, 1.5 equiv.), 1-iodo-4-vinylbenzene (46.1 mg, 0.2 mmol, 1.0 equiv.) and Ammonium chloride (21.4 mg, 0.4 mmol, 2.0 equiv.), the product was isolated by flash chromatography (petroleum ether: ethyl acetate = 1: 2) as a brown oil (53.4 mg, 69%). $^1$H NMR (400 MHz, $CDCl_3$) δ 8.54 (d, J = 5.4 Hz, 2H), 7.67 (d, J = 8.2 Hz, 2H), 7.20 (d, J = 5.4 Hz, 2H), 7.03 (d, J = 8.2 Hz, 2H), 4.61 (t, J = 7.2 Hz, 1H), 3.74 (d, J = 7.2 Hz, 2H), 2.53 (s, 3H). $^{13}$C NMR (101 MHz, $CDCl_3$) δ 150.28, 150.05, 139.36, 138.50, 129.72, 122.77, 93.66, 59.25, 44.76, 42.37. HRMS (ESI+): calcd for $C_{14}H_{15}INO_2S^+$ (M+H) 387.9863, found 387.9859.

**4-(1-(4-methoxyphenyl)-2-(methylsulfonyl)ethyl)pyridine (9)**

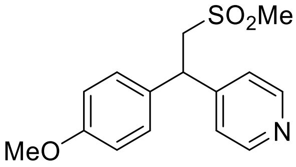


According to the general procedure, 9,10-diphenylanthracene (3.3 mg, 0.01 mmol, 5 mol%), 4-cyanopyridine (41.6 mg, 0.4 mmol, 2.0 equiv.), and Sodium methyl sulfinate (34 mg, 0.3 mmol, 1.5 equiv.), 1-methoxy-4-vinylbenzene (26.6 μL, 0.2 mmol, 1.0 equiv.) and Ammonium chloride (21.4 mg, 0.4 mmol, 2.0 equiv.), the product was isolated by flash chromatography (ethyl acetate) as a pale-yellow oil (55.3 mg, 95%). $^1$H NMR (400 MHz, $CDCl_3$) δ 8.55 (d, J = 5.5 Hz, 2H), 7.26 – 7.15 (m, 4H), 6.89 (d, J = 8.6 Hz, 2H), 4.62 (dd, J = 8.3, 5.9 Hz, 1H), 3.85 – 3.75 (m, 4H), 3.70 (dd, J = 14.6, 5.9 Hz, 1H), 2.45 (s, 3H). $^{13}$C NMR (101 MHz, $CDCl_3$) δ 159.22,

151.17, 149.89, 131.40, 128.97, 122.66, 114.74, 59.77, 55.25, 44.60, 42.17. HRMS (ESI+): calcd for $C_{15}H_{18}NO_3S^+$ (M+H) 292.1002, found 292.1001.

**4-(2-(methylsulfonyl)-1-(pyridin-4-yl)ethyl)phenyl acetate (10)**

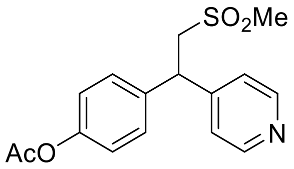


According to the general procedure, 9,10-diphenylanthracene (3.3 mg, 0.01 mmol, 5 mol%), 4-cyanopyridine (41.6 mg, 0.4 mmol, 2.0 equiv.), and Sodium methyl sulfinate (34 mg, 0.3 mmol, 1.5 equiv.), 4-vinylphenyl acetate (30.6 μL, 0.2 mmol, 1.0 equiv.) and Ammonium chloride (21.4 mg, 0.4 mmol, 2.0 equiv.), the product was isolated by flash chromatography (ethyl acetate) as a pale-yellow oil (59.4 mg, 93%). $^1$H NMR (400 MHz, $CDCl_3$) δ 8.49 (d, J = 5.8 Hz, 2H), 7.26 – 7.20 (m, 2H), 7.17 (d, J = 5.8 Hz, 2H), 7.06 – 7.00 (m, 2H), 4.61 (t, J = 7.2 Hz, 1H), 3.69 (d, J = 7.2 Hz, 2H), 2.43 (s, 3H), 2.21 (s, 3H). $^{13}$C NMR (101 MHz, $CDCl_3$) δ 169.16, 150.43, 150.20, 149.94, 137.15, 128.85, 122.84, 122.54, 59.54, 44.67, 42.22, 21.02. HRMS (ESI+): calcd for $C_{16}H_{18}NO_4S^+$ (M+H) 320.0951, found 320.0948.

**4-(2-(methylsulfonyl)-1-(pyridin-4-yl)ethyl)aniline (11)**

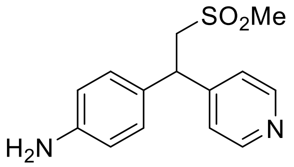


According to the general procedure, 9,10-diphenylanthracene (3.3 mg, 0.01 mmol, 5 mol%), 4-cyanopyridine (41.6 mg, 0.4 mmol, 2.0 equiv.), and Sodium methyl sulfinate (34 mg, 0.3 mmol, 1.5 equiv.), 4-vinylaniline (23.5 μL, 0.2 mmol, 1.0 equiv.) and Ammonium chloride (21.4 mg, 0.4 mmol, 2.0 equiv.), the product was isolated by flash chromatography (ethyl acetate) as a yellow solid (49.2 mg, 89%). $^1$H NMR (400

MHz, $CDCl_3$) δ 8.52 (d, J = 6.0 Hz, 2H), 7.19 (d, J = 6.0 Hz, 2H), 7.05 (d, J = 8.4 Hz, 2H), 6.65 (d, J = 8.4 Hz, 2H), 4.53 (dd, J = 8.8, 5.6 Hz, 1H), 4.08 – 3.77 (brs, 2H), 3.77 – 3.71 (m, 1H), 3.63 (dd, J = 14.6, 5.6 Hz, 1H), 2.42 (s, 3H). $^{13}C$ NMR (101 MHz, $CDCl_3$) δ 151.35, 149.92, 146.26, 128.87, 128.85, 122.59, 115.63, 59.90, 44.70, 42.14. HRMS (ESI+): calcd for $C_{14}H_{17}N_2O_2S^+$ (M+H) 277.1005, found 277.1001.

**4-(2-(methylsulfonyl)-1-(m-tolyl)ethyl)pyridine (12)**

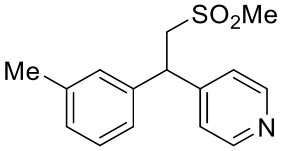


According to the general procedure, 9,10-diphenylanthracene (3.3 mg, 0.01 mmol, 5 mol%), 4-cyanopyridine (41.6 mg, 0.4 mmol, 2.0 equiv.), and Sodium methyl sulfinate (34 mg, 0.3 mmol, 1.5 equiv.), 1-methyl-3-vinylbenzene (26.2 µL, 0.2 mmol, 1.0 equiv.) and Ammonium chloride (21.4 mg, 0.4 mmol, 2.0 equiv.), the product was isolated by flash chromatography (ethyl acetate) as a pale-yellow solid (48.5 mg, 88%). $^1H$ NMR (400 MHz, $CDCl_3$) δ 8.48 (d, J = 5.9 Hz, 2H), 7.24 – 7.12 (m, 3H), 7.03 (d, J = 8.0 Hz, 3H), 4.59 – 4.51 (m, 1H), 3.69 (ddd, J = 20.9, 14.6, 7.2 Hz, 2H), 2.37 (s, 3H), 2.26 (s, 3H). $^{13}C$ NMR (101 MHz, $CDCl_3$) δ 150.75, 150.04, 139.56, 139.26, 129.25, 128.82, 128.61, 124.71, 122.73, 59.67, 45.33, 42.12, 21.37. HRMS (ESI+): calcd for $C_{15}H_{18}NO_2S^+$ (M+H) 276.1053, found 276.1047.

**4-(1-(3-chlorophenyl)-2-(methylsulfonyl)ethyl)pyridine (13)**

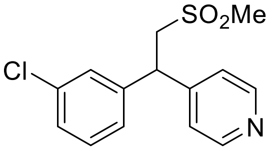


According to the general procedure, 9,10-diphenylanthracene (3.3 mg, 0.01 mmol, 5 mol%), 4-cyanopyridine (41.6 mg, 0.4 mmol, 2.0 equiv.), and Sodium methyl

sulfinate (34 mg, 0.3 mmol, 1.5 equiv.), 1-chloro-3-vinylbenzene (25.4 μL, 0.2 mmol, 1.0 equiv.) and Ammonium chloride (21.4 mg, 0.4 mmol, 2.0 equiv.), the product was isolated by flash chromatography (ethyl acetate) as a pale-yellow oil (41.4 mg, 70%). $^{1}$H NMR (400 MHz, $CDCl_3$) δ 8.51 (d, J = 5.4 Hz, 2H), 7.25 – 7.19 (m, 3H), 7.16 (d, J = 5.4 Hz, 2H), 7.12 (dt, J = 7.1, 1.6 Hz, 1H), 4.59 (t, J = 7.1 Hz, 1H), 3.69 (d, J = 7.1 Hz, 2H), 2.48 (s, 3H). $^{13}$C NMR (101 MHz, $CDCl_3$) δ 150.19, 149.98, 141.74, 135.26, 130.65, 128.32, 127.93, 126.04, 122.79, 59.26, 44.85, 42.33. HRMS (ESI+): calcd for $C_{14}H_{15}ClNO_2S^+$ (M+H) 296.0507, found 296.0505.

**4-(2-(methylsulfonyl)-1-(3-(trifluoromethyl)phenyl)ethyl)pyridine (14)**

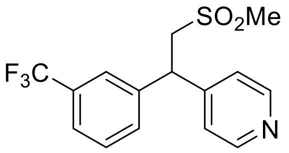


According to the general procedure, 9,10-diphenylanthracene (3.3 mg, 0.01 mmol, 5 mol%), 4-cyanopyridine (41.6 mg, 0.4 mmol, 2.0 equiv.), and Sodium methyl sulfinate (34 mg, 0.3 mmol, 1.5 equiv.), 1-(trifluoromethyl)-3-vinylbenzene (34.4 mg, 0.2 mmol, 1.0 equiv.) and Ammonium chloride (21.4 mg, 0.4 mmol, 2.0 equiv.), the product was isolated by flash chromatography (ethyl acetate) as a pale-yellow oil (53.3 mg, 81%). $^{1}$H NMR (400 MHz, $CDCl_3$) δ 8.60 (d, J = 5.2 Hz, 2H), 7.60 – 7.47 (m, 4H), 7.24 (d, J = 5.2 Hz, 2H), 4.77 (t, J = 7.1 Hz, 1H), 3.85 – 3.74 (m, 2H), 2.57 (s, 3H). $^{13}$C NMR (101 MHz, $CDCl_3$) δ 150.49, 149.55, 140.90, 131.70 (q, J = 32.3 Hz), 131.31, 129.91, 124.91 (q, J = 3.0 Hz), 124.38 (q, J = 3.6 Hz), 123.62 (q, J = 273.7 Hz), 122.72, 59.17, 44.85, 42.31. $^{19}$F NMR (377 MHz, $CDCl_3$) δ -62.64. HRMS (ESI+): calcd for $C_{15}H_{15}F_3NO_2S^+$ (M+H) 330.0770, found 330.0767.

**4-(2-(methylsulfonyl)-1-(o-tolyl)ethyl)pyridine (15)**

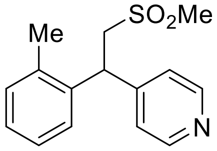


According to the general procedure, 9,10-diphenylanthracene (3.3 mg, 0.01 mmol, 5 mol%), 4-cyanopyridine (41.6 mg, 0.4 mmol, 2.0 equiv.), and Sodium methyl sulfinate (34 mg, 0.3 mmol, 1.5 equiv.), 1-methyl-2-vinylbenzene (25.7 μL, 0.2 mmol, 1.0 equiv.) and Ammonium chloride (21.4 mg, 0.4 mmol, 2.0 equiv.), the product was isolated by flash chromatography (ethyl acetate) as a pale-yellow oil (50.7 mg, 92%). $^1$H NMR (600 MHz, $CDCl_3$) δ 8.52 (d, J = 4.9 Hz, 2H), 7.25 – 7.16 (m, 6H), 4.92 (t, J = 7.1 Hz, 1H), 3.75 (ddd, J = 20.9, 14.6, 7.1 Hz, 2H), 2.44 (s, 3H), 2.39 (s, 3H). $^{13}$C NMR (101 MHz, $CDCl_3$) δ 150.35, 149.94, 137.66, 136.33, 131.42, 127.85, 126.77, 126.74, 122.99, 59.68, 42.09, 40.59, 19.72. HRMS (ESI+): calcd for $C_{15}H_{18}NO_2S^+$ (M+H) 276.1053, found 276.1046.

**4-(2-(methylsulfonyl)-1-(naphthalen-2-yl)ethyl)pyridine (16)**

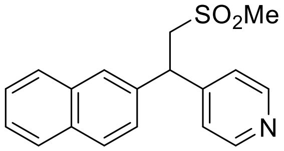


According to the general procedure, 9,10-diphenylanthracene (3.3 mg, 0.01 mmol, 5 mol%), 4-cyanopyridine (41.6 mg, 0.4 mmol, 2.0 equiv.), and Sodium methyl sulfinate (34 mg, 0.3 mmol, 1.5 equiv.), 2-vinylnaphthalene (30.8 mg, 0.2 mmol, 1.0 equiv.) and Ammonium chloride (21.4 mg, 0.4 mmol, 2.0 equiv.), the product was isolated by flash chromatography (ethyl acetate) as a pale-yellow oil (48.0 mg, 77%). $^1$H NMR (400 MHz, $CDCl_3$) δ 8.57 (d, J = 6.0 Hz, 2H), 7.88 – 7.77 (m, 4H), 7.57 – 7.47 (m, 2H), 7.34 (m, 1H), 7.30 – 7.25 (m, 2H), 4.90 – 4.81 (m, 1H), 3.88 (ddd, J = 20.8, 14.6, 7.1 Hz, 2H), 2.44 (s, 3H). $^{13}$C NMR (101 MHz, $CDCl_3$) δ 150.39, 150.28,

136.88, 133.32, 132.65, 129.51, 127.84, 127.73, 126.92, 126.91, 126.68, 125.17, 122.81, 59.57, 45.44, 42.25. HRMS (ESI+): calcd for $C_{18}H_{18}NO_2S^+$ (M+H) 312.1053, found 312.1048.

**4-(1-(benzofuran-5-yl)-2-(methylsulfonyl)ethyl)pyridine (17)**

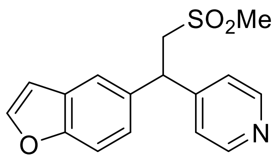


According to the general procedure, 9,10-diphenylanthracene (3.3 mg, 0.01 mmol, 5 mol%), 4-cyanopyridine (41.6 mg, 0.4 mmol, 2.0 equiv.), and Sodium methyl sulfinate (34 mg, 0.3 mmol, 1.5 equiv.), 5-vinylbenzofuran (27.7 μL, 0.2 mmol, 1.0 equiv.) and Ammonium chloride (21.4 mg, 0.4 mmol, 2.0 equiv.), the product was isolated by flash chromatography (ethyl acetate) as a pale-yellow oil (51.8 mg, 86%). $^1$H NMR (400 MHz, $CDCl_3$) δ 8.53 (d, J = 5.8 Hz, 2H), 7.63 (d, J = 2.1 Hz, 1H), 7.54 (d, J = 1.3 Hz, 1H), 7.48 (d, J = 8.5 Hz, 1H), 7.25 – 7.16 (m, 3H), 6.73 (d, J = 2.1 Hz, 1H), 4.76 (dd, J = m, 1H), 3.81 (ddd, J = 20.6, 14.6, 7.2 Hz, 2H), 2.40 (s, 3H). $^{13}$C NMR (101 MHz, $CDCl_3$) δ 154.21, 151.25, 149.83, 146.19, 134.21, 128.27, 123.87, 122.74, 120.61, 112.32, 106.50, 59.91, 45.23, 42.16. HRMS (ESI+): calcd for $C_{16}H_{16}NO_3S^+$ (M+H) 302.0845, found 302.0843.

**4-(2-(methylsulfonyl)-1,2,3,4-tetrahydronaphthalen-1-yl)pyridine (18)**

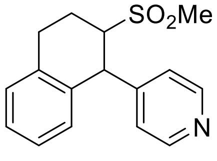


According to the general procedure, 9,10-diphenylanthracene (3.3 mg, 0.01 mmol, 5 mol%), 4-cyanopyridine (41.6 mg, 0.4 mmol, 2.0 equiv.), and Sodium methyl

sulfinate (34 mg, 0.3 mmol, 1.5 equiv.), 1,2-dihydronaphthalene (26.1 μL, 0.2 mmol, 1.0 equiv.) and Ammonium chloride (21.4 mg, 0.4 mmol, 2.0 equiv.), the product was isolated by flash chromatography (ethyl acetate) as a pale-yellow oil (45.4 mg, 79%). $^1$H NMR (400 MHz, $CDCl_3$) δ 8.62 – 8.51 (m, 2H), 7.19 (m, 2H), 7.17 – 7.05 (m, 3H), 6.88 – 6.76 (m, 1H), 4.78 – 4.66 (m, 1H), 3.49 (m, 1H), 3.09 (m, 1H), 3.04 – 2.92 (m, 1H), 2.71 (s, 1H), 2.60 (d, J = 3.5 Hz, 3H), 2.54 – 2.45 (m, 1H), 2.21 – 2.09 (m, 1H). $^{13}$C NMR (101 MHz, $CDCl_3$) δ 153.41, 150.14, 135.59, 134.63, 129.99, 128.71, 127.20, 126.99, 124.13, 66.32, 43.98, 40.25, 27.22, 21.51. HRMS (ESI+): calcd for $C_{16}H_{18}NO_2S^+$ (M+H) 288.1053, found 288.1051.

**4-(1-(methylsulfonyl)-2-phenylpropan-2-yl)pyridine (19)**

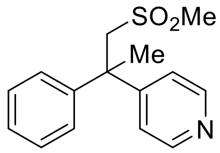


According to the general procedure, 9,10-diphenylanthracene (3.3 mg, 0.01 mmol, 5 mol%), 4-cyanopyridine (41.6 mg, 0.4 mmol, 2.0 equiv.), and Sodium methyl sulfinate (34 mg, 0.3 mmol, 1.5 equiv.), prop-1-en-2-ylbenzene (26.0 μL, 0.2 mmol, 1.0 equiv.) and Ammonium chloride (21.4 mg, 0.4 mmol, 2.0 equiv.), the product was isolated by flash chromatography (ethyl acetate) as a pale-yellow oil (53.9 mg, 98%). $^1$H NMR (600 MHz, $CDCl_3$) δ 8.52 (d, J = 5.9 Hz, 2H), 7.34 (t, J = 7.6 Hz, 2H), 7.29 (d, J = 7.6 Hz, 1H), 7.24 – 7.16 (m, 2H), 7.09 (m, 2H), 3.84 (dd, J = 38.7, 14.6 Hz, 2H), 2.09 (s, 3H), 1.98 (s, 3H). $^{13}$C NMR (101 MHz, $CDCl_3$) δ 156.47, 149.88, 143.72, 128.71, 127.59, 127.55, 121.81, 64.87, 45.36, 42.61, 26.73.[1]

**4-(1-((methylsulfonyl)methyl)-1,2,3,4-tetrahydronaphthalen-1-yl)pyridine (20)**

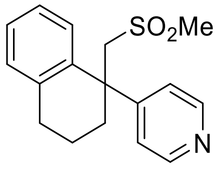


According to the general procedure, 9,10-diphenylanthracene (3.3 mg, 0.01 mmol, 5 mol%), 4-cyanopyridine (41.6 mg, 0.4 mmol, 2.0 equiv.), and Sodium methyl sulfinate (34 mg, 0.3 mmol, 1.5 equiv.), 1-methylene-1,2,3,4-tetrahydronaphthalene (28.5 μL, 0.2 mmol, 1.0 equiv.) and Ammonium chloride (21.4 mg, 0.4 mmol, 2.0 equiv.), the product was isolated by flash chromatography (ethyl acetate) as a pale-yellow oil (43.9 mg, 73%). $^{1}$H NMR (600 MHz, $CDCl_3$) δ 8.49 (d, J = 4.7 Hz, 2H), 7.23 (dd, J = 7.9, 1.5 Hz, 2H), 7.20 – 7.15 (m, 1H), 7.04 (d, J = 7.9 Hz, 1H), 6.98 (d, J = 6.0 Hz, 2H), 3.95 – 3.85 (m, 2H), 2.97 – 2.86 (m, 2H), 2.77 (dt, J = 16.6, 3.5 Hz, 1H), 2.35 (s, 3H), 2.07 (dd, J = 13.0, 3.5 Hz, 1H), 1.80 (ddd, J = 17.1, 8.6, 5.2 Hz, 1H), 1.53 – 1.42 (m, 1H). $^{13}$C NMR (101 MHz, $CDCl_3$) δ 156.87, 149.70, 139.40, 136.02, 130.22, 129.23, 127.62, 126.09, 122.24, 64.50, 45.79, 43.61, 35.33, 29.63, 18.76. HRMS (ESI+): calcd for $C_{17}H_{20}NO_2S^+$ (M+H) 302.1209, found 302.1206.

**3-(4,5-diphenyloxazol-2-yl)-N-(4-(2-(methylsulfonyl)-1-(pyridin-4-yl)ethyl)phenyl )propanamide (21)**

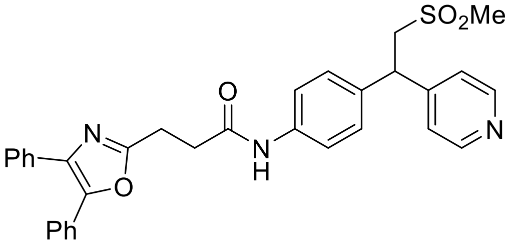


According to the general procedure, 9,10-diphenylanthracene (3.3 mg, 0.01 mmol, 5 mol%), isonicotinonitrile (41.6 mg, 0.4 mmol, 2.0 equiv.), and sodium methanesulfinate (30.6 mg, 0.3 mmol, 1.5 equiv.),

3-(4,5-diphenyloxazol-2-yl)-N-(4-vinylphenyl)propanamide (78.9 mg, 0.2 mmol, 1.0 equiv.) and Ammonium chloride (21.4 mg, 0.4 mmol, 2.0 equiv.), the product was isolated by flash chromatography (ethyl acetate) as a white solid (107.0 mg, 97%). $^1$H NMR (600 MHz, $CDCl_3$) δ 9.15 (s, 1H), 8.48 (d, J = 5.1 Hz, 2H), 7.57 (dd, J = 7.6, 1.4 Hz, 2H), 7.51 (d, J = 8.0 Hz, 4H), 7.35 – 7.28 (m, 6H), 7.15 (t, J = 7.1 Hz, 4H), 4.62 – 4.51 (m, 1H), 3.74 (dd, J = 14.5, 8.3 Hz, 1H), 3.65 (dd, J = 14.5, 6.1 Hz, 1H), 3.22 (t, J = 7.0 Hz, 2H), 2.90 (t, J = 7.0 Hz, 2H), 2.45 (s, 3H). $^{13}$C NMR (101 MHz, $CDCl_3$) δ 170.14, 162.44, 150.69, 150.21, 145.72, 138.19, 134.98, 134.75, 132.21, 128.72, 128.68, 128.61, 128.45, 128.32, 127.83, 126.47, 122.72, 120.43, 59.55, 44.80, 42.30, 33.93, 23.90. HRMS (ESI+): calcd for $C_{32}H_{30}N_3O_4S^+$ (M+H) 552.1952, found 552.1949.

**(8R,9S,13S,14S)-13-methyl-3-(2-(methylsulfonyl)-1-(pyridin-4-yl)ethyl)-6,7,8,9,11,12,13,14,15,16-decahydro-17H-cyclopenta$^2$phenanthren-17-one (22)**

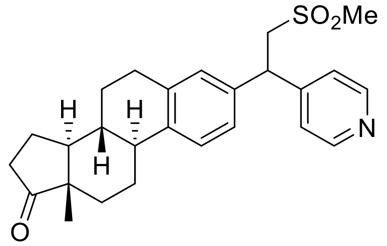


According to the general procedure, 9,10-diphenylanthracene (3.3 mg, 0.01 mmol, 5 mol%), isonicotinonitrile (41.6 mg, 0.4 mmol, 2.0 equiv.), and sodium methanesulfinate (30.6 mg, 0.3 mmol, 1.5 equiv.), (8R,9S,13S,14S)-13-methyl-3-vinyl-6,7,8,9,11,12,13,14,15,16-decahydro-17H-cyclopenta$^2$phenanthren-17-one (56.1 mg, 0.2 mmol, 1.0 equiv.) and Ammonium chloride (21.4 mg, 0.4 mmol, 2.0 equiv.), the product was isolated by flash chromatography (ethyl acetate) as a white solid (59.5 mg, 68%). $^1$H NMR (400 MHz, $CDCl_3$) δ 8.55 (d, J = 5.4 Hz, 2H), 7.29 – 7.23 (m, 3H), 7.06 (d, J = 8.1 Hz, 1H), 7.00 (d, J = 5.4 Hz, 1H),

4.60 (t, J = 7.0 Hz, 1H), 3.83 – 3.68 (m, 2H), 2.96 – 2.81 (m, 2H), 2.58 – 2.45 (m, 4H), 2.43 – 2.35 (m, 1H), 2.26 (m, 1H), 2.15 (m, 1H), 2.06 (m, 2H), 1.97 – 1.92 (m, 1H), 1.67 – 1.41 (m, 6H), 0.90 (s, 3H). $^{13}$C NMR (101 MHz, $CDCl_3$) δ 219.56, 149.93, 149.13, 138.76 (d, J = 2.3 Hz), 136.74, 136.05 (d, J = 6.4 Hz), 127.38 (d, J = 20.4 Hz), 125.36 (d, J = 3.6 Hz), 123.82 (d, J = 20.1 Hz), 121.82, 58.67, 49.43, 46.87, 43.99 (d, J = 3.8 Hz), 43.18, 41.23, 36.92, 34.76, 30.49, 28.33, 25.28, 24.55, 20.51, 12.79. HRMS (ESI+): calcd for $C_{26}H_{32}NO_3S^+$ (M+H) 438.2097, found 438.2096.

**2-(4-(2-(methylsulfonyl)-1-(pyridin-4-yl)ethyl)benzyl)isoindoline-1,3-dione (23)**

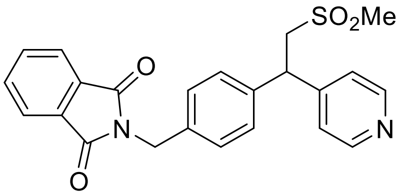


According to the general procedure, 9,10-diphenylanthracene (3.3 mg, 0.01 mmol, 5 mol%), isonicotinonitrile (41.6 mg, 0.4 mmol, 2.0 equiv.), and sodium methanesulfinate (30.6 mg, 0.3 mmol, 1.5 equiv.), 2-(4-vinylbenzyl)isoindoline-1,3-dione (52.6 mg, 0.2 mmol, 1.0 equiv.) and Ammonium chloride (21.4 mg, 0.4 mmol, 2.0 equiv.), the product was isolated by flash chromatography (ethyl acetate) as a white solid (60.5 mg, 72%). $^{1}$H NMR (400 MHz, DMSO-d6) δ 8.44 (s, 2H), 7.85 (ddd, J = 7.7, 5.0, 2.1 Hz, 4H), 7.48 – 7.34 (m, 4H), 7.29 – 7.22 (m, 2H), 4.71 (s, 2H), 4.58 (d, J = 4.9 Hz, 1H), 4.11 (m, 1H), 4.01 (m, 1H), 2.79 (s, 3H). $^{13}$C NMR (101 MHz, DMSO-d6) δ 167.69, 151.32, 149.69, 140.86, 135.50, 134.55, 131.53, 127.95, 127.81, 123.22, 122.94, 56.89, 43.82, 41.37, 40.46. HRMS (ESI+): calcd for $C_{23}H_{21}N_2O_4S^+$ (M+H) 421.1217, found 421.1212.

**2-(1-(4-chlorobenzoyl)-6-methoxy-2-methyl-1H-indol-3-yl)-N-(4-(2-(methylsulfonyl)-1-(pyridin-4-yl)ethyl)phenyl)acetamide (24)**

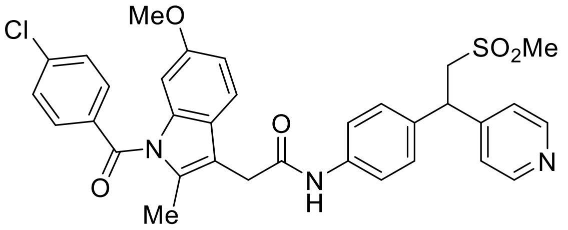


According to the general procedure, 9,10-diphenylanthracene (3.3 mg, 0.01 mmol, 5 mol%), isonicotinonitrile (41.6 mg, 0.4 mmol, 2.0 equiv.), and sodium methanesulfinate (30.6 mg, 0.3 mmol, 1.5 equiv.), 2-(1-(4-chlorobenzoyl)-6-methoxy-2-methyl-1H-indol-3-yl)-N-(4-vinylphenyl)acetamide (91.6 mg, 0.2 mmol, 1.0 equiv.) and Ammonium chloride (21.4 mg, 0.4 mmol, 2.0 equiv.), the product was isolated by flash chromatography (ethyl acetate) as a white solid (73.8 mg, 60 %). $^1$H NMR (400 MHz, $CDCl_3$) δ 8.51 (d, J = 5.4 Hz, 2H), 7.65 (d, J = 8.4 Hz, 2H), 7.54 (d, J = 7.5 Hz, 1H), 7.47 (d, J = 8.5 Hz, 2H), 7.41 (d, J = 8.6 Hz, 2H), 7.17 (t, J = 6.8 Hz, 4H), 6.92 (d, J = 2.4 Hz, 1H), 6.85 (d, J = 9.0 Hz, 1H), 6.69 (dd, J = 9.0, 2.5 Hz, 1H), 4.65 – 4.55 (m, 1H), 3.78 (s, 3H), 3.77 (s, 2H), 3.75 – 3.62 (m, 2H), 2.46 (s, 3H), 2.42 (s, 3H). $^{13}$C NMR (101 MHz, $CDCl_3$) δ 168.42, 168.36, 156.37, 150.63, 150.22, 139.75, 137.36, 136.74, 135.62, 133.41, 131.24, 130.99, 130.15, 129.28, 128.45, 122.70, 120.91, 115.21, 112.22, 112.16, 100.98, 59.52, 55.81, 44.76, 42.33, 33.27, 13.33. HRMS (ESI+): calcd for $C_{33}H_{31}ClN_3O_5S^+$ (M+H) 616.1667, found 616.1666.

**4-(1-(4-(tert-butyl)phenyl)-2-(methylsulfonyl)ethyl)-2-methylpyridine (25)**

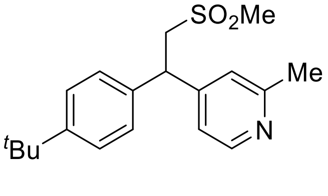

According to the general procedure, 9,10-diphenylanthracene (3.3 mg, 0.01 mmol, 5 mol%), 2-methylisonicotinonitrile (47.3 mg, 0.4 mmol, 2.0 equiv.), and Sodium methyl sulfinate (34 mg, 0.3 mmol, 1.5 equiv.), 1-(tert-butyl)-4-vinylbenzene (36.6 μL, 0.2 mmol, 1.0 equiv.) and Ammonium chloride (21.4 mg, 0.4 mmol, 2.0 equiv.), the product was isolated by flash chromatography (ethyl acetate) as a yellow solid (55.4 mg, 84%). $^1$H NMR (600 MHz, $CDCl_3$) δ 8.41 (d, J = 5.2 Hz, 1H), 7.35 (d, J = 8.2 Hz, 2H), 7.20 (d, J = 8.2 Hz, 2H), 7.09 (s, 1H), 7.03 (d, J = 5.2 Hz, 1H), 4.56 (t, J = 7.1 Hz, 1H), 3.80 – 3.64 (m, 2H), 2.51 (s, 3H), 2.40 (s, 3H), 1.26 (s, 9H). $^{13}$C NMR (101 MHz, $CDCl_3$) δ 158.98, 151.00, 150.93, 149.41, 136.71, 127.35, 126.17, 122.33, 119.68, 59.78, 44.98, 42.00, 34.43, 31.14, 24.27. HRMS (ESI+): calcd for $C_{19}H_{26}NO_2S^+$ (M+H) 332.1679, found 332.1679.

**2-bromo-4-(1-(4-(tert-butyl)phenyl)-2-(methylsulfonyl)ethyl)pyridine (26)**

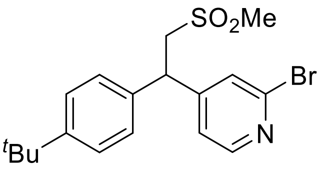


According to the general procedure, 9,10-diphenylanthracene (3.3 mg, 0.01 mmol, 5 mol%), 2-bromoisonicotinonitrile (73.2 mg, 0.4 mmol, 2.0 equiv.), and Sodium methyl sulfinate (34 mg, 0.3 mmol, 1.5 equiv.), 1-(tert-butyl)-4-vinylbenzene (36.6 μL, 0.2 mmol, 1.0 equiv.) and Ammonium chloride (21.4 mg, 0.4 mmol, 2.0 equiv.), the product was isolated by flash chromatography (ethyl acetate) as a pale-yellow oil (65.8 mg, 83%). $^1$H NMR (400 MHz, $CDCl_3$) δ 8.31 (d, J = 5.1 Hz, 1H), 7.47 – 7.31 (m, 3H), 7.21 – 7.11 (m, 3H), 4.61 (t, J = 7.1 Hz, 1H), 3.72 (ddd, J = 20.6, 14.5, 7.1 Hz, 2H), 2.48 (s, 3H), 1.30 (s, 9H). $^{13}$C NMR (101 MHz, $CDCl_3$) δ 153.57, 151.55, 150.55, 142.89, 135.84, 127.46, 126.90, 126.49, 121.90, 59.45, 44.60, 42.18, 34.57, 31.20. HRMS (ESI+): calcd for $C_{18}H_{23}BrNO_2S^+$ (M+H) 396.0627, found 396.0624.

**4-(1-(4-(tert-butyl)phenyl)-2-(methylsulfonyl)ethyl)-2-phenylpyridine (27)**

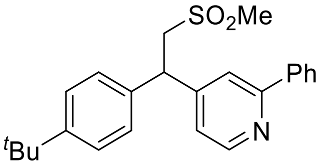


According to the general procedure, 9,10-diphenylanthracene (3.3 mg, 0.01 mmol, 5 mol%), 2-phenylisonicotinonitrile (72.0 mg, 0.4 mmol, 2.0 equiv.), and Sodium methyl sulfinate (34 mg, 0.3 mmol, 1.5 equiv.), 1-(tert-butyl)-4-vinylbenzene (36.6 μL, 0.2 mmol, 1.0 equiv.) and Ammonium chloride (21.4 mg, 0.4 mmol, 2.0 equiv.), the product was isolated by flash chromatography (ethyl acetate) as a pale-yellow oil (70.8 mg, 90%). $^1$H NMR (400 MHz, $CDCl_3$) δ 8.63 (d, J = 5.1 Hz, 1H), 7.99 – 7.90 (m, 2H), 7.66 (s, 1H), 7.49 – 7.36 (m, 5H), 7.28 – 7.24 (m, 2H), 7.16 (dd, J = 5.1, 1.5 Hz, 1H), 4.70 (t, J = 7.1 Hz, 1H), 3.89 – 3.72 (m, 2H), 2.44 (s, 3H), 1.29 (s, 9H). $^{13}$C NMR (101 MHz, $CDCl_3$) δ 158.23, 151.29, 151.16, 150.20, 138.93, 136.68, 129.21, 128.75, 127.44, 126.99, 126.30, 120.93, 119.73, 59.92, 45.28, 42.12, 34.51, 31.20. HRMS (ESI+): calcd for $C_{24}H_{28}NO_2S^+$ (M+H) 394.1835, found 394.1831.

**4-(1-(4-(tert-butyl)phenyl)-2-(methylsulfonyl)ethyl)-2-methoxypyridine (28)**

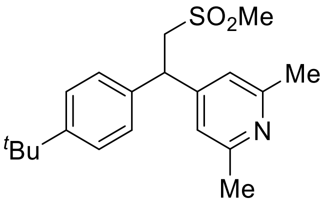


According to the general procedure, 9,10-diphenylanthracene (3.3 mg, 0.01 mmol, 5 mol%), 2,6-dimethylisonicotinonitrile (52.8 mg, 0.4 mmol, 2.0 equiv.), and Sodium methyl sulfinate (34 mg, 0.3 mmol, 1.5 equiv.), 1-(tert-butyl)-4-vinylbenzene (36.6 μL, 0.2 mmol, 1.0 equiv.) and Ammonium chloride (21.4 mg, 0.4 mmol, 2.0 equiv.), the product was isolated by flash chromatography (ethyl acetate) as a pale-yellow oil (61.8 mg, 89%). $^1$H NMR (600 MHz, $CDCl_3$) δ 7.36 (d, J = 8.4 Hz, 2H), 7.21 (d, J =

8.4 Hz, 2H), 6.89 (s, 2H), 4.53 (t, J = 7.2 Hz, 1H), 3.72 (qd, J = 14.7, 7.2 Hz, 2H), 2.49 (s, 6H), 2.39 (s, 3H), 1.28 (s, 9H). $^{13}$C NMR (101 MHz, $CDCl_3$) δ 158.50, 150.99, 150.96, 136.93, 127.37, 126.21, 119.18, 60.01, 45.08, 42.05, 34.51, 31.23, 24.53. HRMS (ESI+): calcd for $C_{20}H_{28}NO_2S^+$ (M+H) 346.1835, found 346.1832.

**4-(1-(4-(tert-butyl)phenyl)-2-(methylsulfonyl)ethyl)-3-methylpyridine (29)**

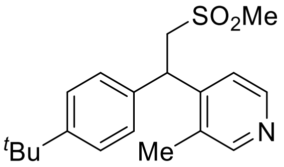


According to the general procedure, 9,10-diphenylanthracene (3.3 mg, 0.01 mmol, 5 mol%), 3-methylisonicotinonitrile (47.3 mg, 0.4 mmol, 2.0 equiv.), and Sodium methyl sulfinate (34 mg, 0.3 mmol, 1.5 equiv.), 1-(tert-butyl)-4-vinylbenzene (36.6 μL, 0.2 mmol, 1.0 equiv.) and Ammonium chloride (21.4 mg, 0.4 mmol, 2.0 equiv.), the product was isolated by flash chromatography (petroleum ether: ethyl acetate = 1: 2) as a colorless oil (58.1 mg, 88%). $^{1}$H NMR (600 MHz, $CDCl_3$) δ 8.45 (d, J = 4.9 Hz, 1H), 8.37 (s, 1H), 7.33 (d, J = 8.2 Hz, 2H), 7.21 (dd, J = 13.2, 6.7 Hz, 3H), 4.81 (t, J = 7.1 Hz, 1H), 3.72 (ddd, J = 21.1, 14.6, 7.1 Hz, 2H), 2.40 (s, 3H), 2.35 (s, 3H), 1.26 (s, 9H). $^{13}$C NMR (101 MHz, $CDCl_3$) 13C NMR (101 MHz, CDCl3) δ 151.42, 150.97, 148.54, 147.70, 135.75, 131.69, 127.73, 126.07, 120.81, 59.93, 42.11, 40.92, 34.42, 31.14, 16.49. HRMS (ESI+): calcd for $C_{19}H_{26}NO_2S^+$ (M+H) 332.1679, found 332.1673.

**4-(1-(4-(tert-butyl)phenyl)-2-(methylsulfonyl)ethyl)-3-chloropyridine (30)**

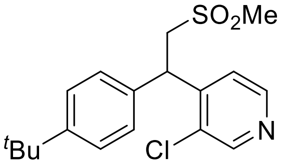

According to the general procedure, 9,10-diphenylanthracene (3.3 mg, 0.01 mmol, 5 mol%), 3-chloroisonicotinonitrile (55.4 mg, 0.4 mmol, 2.0 equiv.), and Sodium methyl sulfinate (34 mg, 0.3 mmol, 1.5 equiv.), 1-(tert-butyl)-4-vinylbenzene (36.6 μL, 0.2 mmol, 1.0 equiv.) and Ammonium chloride (21.4 mg, 0.4 mmol, 2.0 equiv.), the product was isolated by flash chromatography(petroleum ether: ethyl acetate = 3: 1) as a pale-yellow oil (68.6 mg, 97%). $^1$H NMR (600 MHz, $CDCl_3$) δ 8.56 (s, 1H), 8.47 (d, J = 5.0 Hz, 1H), 7.37 (d, J = 8.2 Hz, 2H), 7.32 – 7.25 (m, 3H), 5.16 – 5.07 (m, 1H), 3.77 (ddd, J = 20.8, 14.6, 7.2 Hz, 2H), 2.53 (s, 3H), 1.28 (s, 9H). $^{13}$C NMR (101 MHz, $CDCl_3$) δ 151.21, 150.05, 148.03, 147.53, 134.77, 131.50, 127.78, 126.08, 122.62, 58.76, 41.89, 41.32, 34.43, 31.11. HRMS (ESI+): calcd for $C_{18}H_{23}ClNO_2S^+$ (M+H) 352.1133, found 352.1130.

**4-(1-(4-(tert-butyl)phenyl)-2-(methylsulfonyl)ethyl)nicotinonitrile (31)**

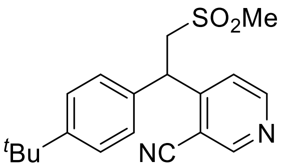


According to the general procedure, 9,10-diphenylanthracene (3.3 mg, 0.01 mmol, 5 mol%), pyridine-3,4-dicarbonitrile (51.7 mg, 0.4 mmol, 2.0 equiv.), and Sodium methyl sulfinate (34 mg, 0.3 mmol, 1.5 equiv.), 1-(tert-butyl)-4-vinylbenzene (36.6 μL, 0.2 mmol, 1.0 equiv.) and Ammonium chloride (21.4 mg, 0.4 mmol, 2.0 equiv.), the product was isolated by flash chromatography (petroleum ether: ethyl acetate = 1: 1) as a white solid (65.9mg, 96%). $^1$H NMR (600 MHz, $CDCl_3$) δ 8.80 (s, 1H), 8.76 (d, J = 5.3 Hz, 1H), 7.49 (d, J = 5.3 Hz, 1H), 7.39 (d, J = 8.3 Hz, 2H), 7.29 (d, J = 8.8 Hz, 2H), 5.01 (t, J = 7.3 Hz, 1H), 3.88 (d, J = 7.3 Hz, 2H), 2.69 (s, 3H), 1.28 (s, 9H). $^{13}$C NMR (101 MHz, $CDCl_3$) δ 153.50, 153.13, 151.66, 134.72, 127.43, 126.39, 121.81,

115,71, 109.95, 58.21, 43.29, 41.85, 34.47, 31.07. HRMS (ESI+): calcd for $C_{19}H_{23}N_2O_2S^+$ (M+H) 343.1475, found 343.1471.

**2-(1-(4-(tert-butyl)phenyl)-2-(methylsulfonyl)ethyl)pyridine (32)**

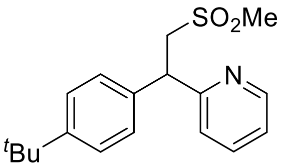


According to the general procedure, 9,10-diphenylanthracene (3.3 mg, 0.01 mmol, 5 mol%), picolinonitrile (41.6 mg, 0.4 mmol, 2.0 equiv.), and Sodium methyl sulfinate (34 mg, 0.3 mmol, 1.5 equiv.), 1-(tert-butyl)-4-vinylbenzene (36.6 μL, 0.2 mmol, 1.0 equiv.) and Ammonium chloride (21.4 mg, 0.4 mmol, 2.0 equiv.), the product was isolated by flash chromatography (petroleum ether: ethyl acetate = 1: 2) as a pale-yellow oil (45.7mg, 72%). $^1$H NMR (400 MHz, $CDCl_3$) δ 8.60 (d, J = 4.6 Hz, 1H), 7.60 (t, J = 7.8 Hz, 1H), 7.31 (q, J = 8.4 Hz, 4H), 7.24 (d, J = 7.8 Hz, 1H), 7.19 – 7.14 (m, 1H), 4.76 – 4.65 (m, 1H), 4.42 (dd, J = 14.6, 8.0 Hz, 1H), 3.62 (dd, J = 14.6, 5.7 Hz, 1H), 2.41 (s, 3H), 1.27 (s, 9H). $^{13}$C NMR (101 MHz, $CDCl_3$) δ 159.81, 150.51, 149.16, 137.98, 136.99, 127.54, 125.94, 123.95, 122.24, 59.59, 47.38, 42.02, 34.44, 31.25. HRMS (ESI+): calcd for $C_{18}H_{24}NO_2S^+$ (M+H) 318.1522, found 318.1519.

**2-(1-(4-(tert-butyl)phenyl)-2-(methylsulfonyl)ethyl)-5-fluoropyridine (33)**

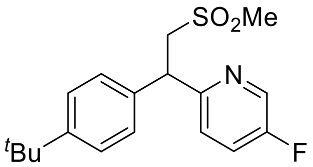


According to the general procedure, 9,10-diphenylanthracene (3.3 mg, 0.01 mmol, 5 mol%), 5-fluoropicolinonitrile (48.8 mg, 0.4 mmol, 2.0 equiv.), and Sodium methyl sulfinate (34 mg, 0.3 mmol, 1.5 equiv.), 1-(tert-butyl)-4-vinylbenzene (36.6 μL, 0.2 mmol, 1.0 equiv.) and Ammonium chloride (21.4 mg, 0.4 mmol, 2.0 equiv.), the

product was isolated by flash chromatography (petroleum ether: ethyl acetate = 5: 1) as a pale-yellow oil (47.6mg, 71%). $^1$H NMR (400 MHz, $CDCl_3$) δ 8.44 (d, J = 2.4 Hz, 1H), 7.40 – 7.29 (m, 3H), 7.28 – 7.20 (m, 3H), 4.73 (m, 1H), 4.35 (dd, J = 14.6, 7.9 Hz, 1H), 3.62 (dd, J = 14.6, 5.8 Hz, 1H), 2.46 (s, 3H), 1.27 (s, 9H). $^{13}$C NMR (101 MHz, $CDCl_3$) δ 158.56 (d, J = 255.8 Hz), 155.87 (d, J = 3.7 Hz), 150.74, 137.57 (d, J = 45.2 Hz), 137.11, 127.52, 126.06, 124.74 (d, J = 4.2 Hz), 123.90 (d, J = 18.4 Hz), 59.67, 46.58, 42.12, 34.50, 31.27. $^{19}$F NMR (377 MHz, $CDCl_3$) δ -129.11 (dd, J = 8.1, 4.3 Hz). HRMS (ESI+): calcd for $C_{18}H_{23}FNO_2S^+$ (M+H) 336.1428, found 336.1422.

**1-(1-(4-(tert-butyl)phenyl)-2-(methylsulfonyl)ethyl)isoquinoline (34)**

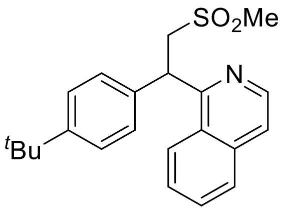


According to the general procedure, 9,10-diphenylanthracene (3.3 mg, 0.01 mmol, 5 mol%), isoquinoline-1-carbonitrile (61.7 mg, 0.4 mmol, 2.0 equiv.), and Sodium methyl sulfinate (34 mg, 0.3 mmol, 1.5 equiv.), 1-(tert-butyl)-4-vinylbenzene (36.6 μL, 0.2 mmol, 1.0 equiv.) and Ammonium chloride (21.4 mg, 0.4 mmol, 2.0 equiv.), the product was isolated by flash chromatography (petroleum ether: ethyl acetate = 4: 1) as a pale-yellow oil (61.8mg, 84%). $^1$H NMR (400 MHz, $CDCl_3$) δ 8.53 (d, J = 5.7 Hz, 1H), 8.27 (d, J = 8.4 Hz, 1H), 7.78 (d, J = 8.1 Hz, 1H), 7.64 – 7.51 (m, 3H), 7.36 (d, J = 8.3 Hz, 2H), 7.28 (d, J = 8.3 Hz, 2H), 5.60 (dd, J = 7.7, 5.7 Hz, 1H), 4.65 (m, 1H), 3.76 (m, 1H), 2.42 (s, 3H), 1.22 (s, 9H). $^{13}$C NMR (101 MHz, $CDCl_3$) δ 158.47, 150.38, 141.10, 137.59, 136.58, 130.05, 127.67, 127.43, 126.53, 125.89, 124.71, 120.50, 60.22, 43.29, 42.20, 34.35, 31.15. HRMS (ESI+): calcd for $C_{22}H_{26}NO_2S^+$ (M+H) 368.1679, found 368.1676.

**4-(1-(4-(tert-butyl)phenyl)-2-(methylsulfonyl)ethyl)-1H-pyrrolo[2,3-b]pyridine (35)**

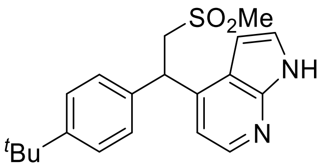


According to the general procedure, 9,10-diphenylanthracene (3.3 mg, 0.01 mmol, 5 mol%), 1H-pyrrolo[2,3-b]pyridine-4-carbonitrile (57.3 mg, 0.4 mmol, 2.0 equiv.), and Sodium methyl sulfinate (34 mg, 0.3 mmol, 1.5 equiv.), 1-(tert-butyl)-4-vinylbenzene (36.6 μL, 0.2 mmol, 1.0 equiv.) and Ammonium chloride (21.4 mg, 0.4 mmol, 2.0 equiv.), the product was isolated by flash chromatography (petroleum ether: ethyl acetate = 1: 2) as a white solid (52.8 mg, 74%). $^1$H NMR (400 MHz, $CDCl_3$) δ 11.44 (s, 1H), 8.29 (d, J = 5.0 Hz, 1H), 7.39 – 7.31 (m, 5H), 7.02 (d, J = 5.0 Hz, 1H), 6.66 (d, J = 3.5 Hz, 1H), 5.08 (m, 1H), 3.93 (dd, J = 6.8, 4.0 Hz, 2H), 2.35 (s, 3H), 1.26 (s, 9H). $^{13}$C NMR (101 MHz, $CDCl_3$) δ 150.72, 148.88, 143.00, 142.68, 136.67, 127.49, 125.99, 125.66, 119.15, 113.58, 99.11, 59.84, 43.23, 41.81, 34.42, 31.18. HRMS (ESI+): calcd for $C_{20}H_{25}N_2O_2S^+$ (M+H) 357.1631, found 357.1630.

**4-(1-(4-(tert-butyl)phenyl)-2-(phenylsulfonyl)ethyl)pyridine (36)**

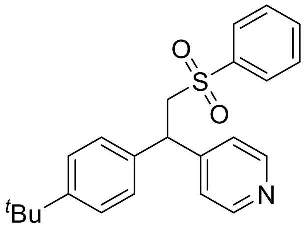


According to the general procedure, 9,10-diphenylanthracene (3.3 mg, 0.01 mmol, 5 mol%), isonicotinonitrile (41.6 mg, 0.4 mmol, 2.0 equiv.), and sodium benzenesulfinate hydrate (60.1 mg, 0.3 mmol, 1.5 equiv.), 1-(tert-butyl)-4-vinylbenzene (36.6 μL, 0.2 mmol, 1.0 equiv.) and Ammonium

chloride (21.4 mg, 0.4 mmol, 2.0 equiv.), the product was isolated by flash chromatography (petroleum ether: ethyl acetate = 1: 1) as a pale-yellow oil (66.0 mg, 87%). $^{1}$H NMR (600 MHz, CDCl$_3$) δ 8.39 (d, J = 5.7 Hz, 2H), 7.63 (dd, J = 8.3, 1.1 Hz, 2H), 7.47 (t, J = 7.5 Hz, 1H), 7.32 (q, J = 7.5 Hz, 2H), 7.18 (d, J = 8.4 Hz, 2H), 7.08 (d, J = 5.7 Hz, 2H), 6.99 (d, J = 8.4 Hz, 2H), 4.57 (t, J = 7.1 Hz, 1H), 3.89 (m, 2H), 1.22 (s, 9H). $^{13}$C NMR (101 MHz, CDCl$_3$) δ 150.71, 150.27, 149.38, 139.13, 136.37, 133.39, 128.86, 127.75, 127.01, 125.75, 122.84, 60.32, 45.08, 34.22, 31.05. HRMS (ESI+): calcd for $C_{23}H_{26}NO_2S^+$ (M+H) 380.1679, found 380.1675.

**4-(1-(4-(tert-butyl)phenyl)-2-((4-chlorophenyl)sulfonyl)ethyl)pyridine (37)**

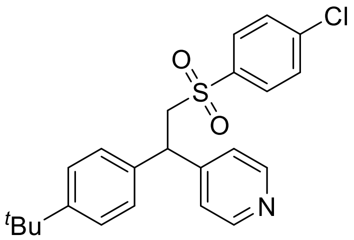


According to the general procedure, 9,10-diphenylanthracene (3.3 mg, 0.01 mmol, 5 mol%), isonicotinonitrile (41.6 mg, 0.4 mmol, 2.0 equiv.), and sodium 4-chlorobenzenesulfinate (59.6 mg, 0.3 mmol, 1.5 equiv.), 1-(tert-butyl)-4-vinylbenzene (36.6 μL, 0.2 mmol, 1.0 equiv.) and Ammonium chloride (21.4 mg, 0.4 mmol, 2.0 equiv.), the product was isolated by flash chromatography (petroleum ether: ethyl acetate = 1: 1) as a pale-yellow oil (69.1 mg, 83%). $^{1}$H NMR (600 MHz, CDCl$_3$) δ 8.45 (d, J = 5.4 Hz, 2H), 7.55 – 7.50 (m, 2H), 7.27 – 7.24 (m, 2H), 7.19 – 7.14 (m, 2H), 7.10 (d, J = 6.0 Hz, 2H), 6.95 (d, J = 8.4 Hz, 2H), 4.57 (t, J = 7.2 Hz, 1H), 3.89 (ddd, J = 21.2, 14.8, 7.2 Hz, 2H), 1.24 (s, 9H). $^{13}$C NMR (101 MHz, CDCl$_3$) δ 151.09, 150.78, 149.47, 140.15, 137.69, 135.70, 129.36, 129.15, 127.19, 125.90, 122.87, 60.55, 45.28, 34.37, 31.19.[3]

**4-(1-(4-(tert-butyl)phenyl)-2-tosylethyl)pyridine (38)**

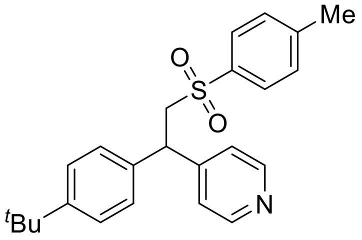


According to the general procedure, 9,10-diphenylanthracene (3.3 mg, 0.01 mmol, 5 mol%), isonicotinonitrile (41.6 mg, 0.4 mmol, 2.0 equiv.), and sodium 4-methylbenzenesulfinate hydrate (75.1 mg, 0.3 mmol, 1.5 equiv.), 1-(tert-butyl)-4-vinylbenzene (36.6 μL, 0.2 mmol, 1.0 equiv.) and Ammonium chloride (21.4 mg, 0.4 mmol, 2.0 equiv.), the product was isolated by flash chromatography (petroleum ether: ethyl acetate = 1: 1) as a pale-yellow oil (63.9 mg, 81%). $^{1}$H NMR (600 MHz, $CDCl_3$) δ 8.40 (d, J = 5.4 Hz, 2H), 7.51 (d, J = 8.4 Hz, 2H), 7.19 (d, J = 8.4 Hz, 2H), 7.12 (d, J = 8.0 Hz, 2H), 7.07 (d, J = 5.9 Hz, 2H), 6.99 (d, J = 8.4 Hz, 2H), 4.55 (t, J = 7.1 Hz, 1H), 3.86 (qd, J = 14.7, 7.1 Hz, 2H), 2.35 (s, 3H), 1.23 (s, 9H). $^{13}$C NMR (101 MHz, $CDCl_3$) δ 150.70, 150.38, 149.59, 144.45, 136.60, 136.29, 129.53, 127.89, 127.08, 125.78, 122.89, 60.60, 45.18, 34.32, 31.16, 21.47.[1]

**4-(1-(4-(tert-butyl)phenyl)-2-((2-chlorophenyl)sulfonyl)ethyl)pyridine (39)**

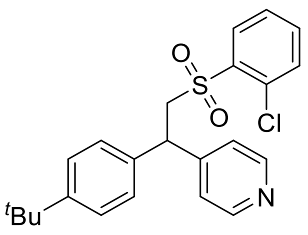


According to the general procedure, 9,10-diphenylanthracene (3.3 mg, 0.01 mmol, 5 mol%), isonicotinonitrile (41.6 mg, 0.4 mmol, 2.0 equiv.), and sodium 2-chlorobenzenesulfinate (59.6 mg, 0.3 mmol, 1.5 equiv.),

1-(tert-butyl)-4-vinylbenzene (36.6 μL, 0.2 mmol, 1.0 equiv.) and Ammonium chloride (21.4 mg, 0.4 mmol, 2.0 equiv.), the product was isolated by flash chromatography (petroleum ether: ethyl acetate = 1: 2) as a pale-yellow oil (74.5 mg, 90%). $^{1}$H NMR (400 MHz, $CDCl_3$) δ 8.32 (d, J = 5.0 Hz, 2H), 7.64 (m, 1H), 7.33 – 7.22 (m, 2H), 7.10 (m, 3H), 7.02 (d, J = 5.5 Hz, 2H), 6.95 (d, J = 8.3 Hz, 2H), 4.49 (t, J = 7.3 Hz, 1H), 4.14 (qd, J = 14.9, 7.3 Hz, 2H), 1.14 (s, 9H). $^{13}$C NMR (101 MHz, $CDCl_3$) δ 150.40, 149.99, 149.79, 136.70, 135.97, 134.51, 132.24, 131.52, 131.36, 127.12, 127.09, 125.77, 122.71, 58.21, 45.36, 34.32, 31.12. HRMS (ESI+): calcd for $C_{23}H_{25}ClNO_2S^+$ (M+H) 414.1289, found 414.1288.

**methyl 2-((2-(4-(tert-butyl)phenyl)-2-(pyridin-4-yl)ethyl)sulfonyl)benzoate (40)**

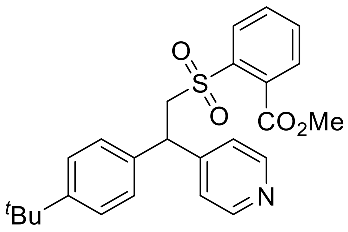


According to the general procedure, 9,10-diphenylanthracene (3.3 mg, 0.01 mmol, 5 mol%), isonicotinonitrile (41.6 mg, 0.4 mmol, 2.0 equiv.), and sodium 2-(methoxycarbonyl)benzenesulfinate (66.7 mg, 0.3 mmol, 1.5 equiv.), 1-(tert-butyl)-4-vinylbenzene (36.6 μL, 0.2 mmol, 1.0 equiv.) and Ammonium chloride (21.4 mg, 0.4 mmol, 2.0 equiv.), the product was isolated by flash chromatography (petroleum ether: ethyl acetate = 1: 2) as a pale-yellow oil (75.3 mg, 86%). $^{1}$H NMR (400 MHz, $CDCl_3$) δ 8.39 (d, J = 5.8 Hz, 2H), 7.57 (t, J = 8.8 Hz, 2H), 7.50 (dd, J = 10.9, 4.2 Hz, 1H), 7.29 – 7.25 (m, 1H), 7.17 (dd, J = 9.0, 7.4 Hz, 4H), 7.09 (d, J = 8.4 Hz, 2H), 4.66 (t, J = 7.4 Hz, 1H), 4.49 – 4.31 (m, 2H), 3.98 (s, 3H), 1.23 (s, 9H). $^{13}$C NMR (101 MHz, $CDCl_3$) δ 167.61, 150.37, 150.20, 149.87, 138.22,

136.45, 133.09, 132.47, 130.66, 130.63, 129.22, 127.31, 125.73, 122.92, 60.56, 53.23, 45.45, 34.34, 31.18. HRMS (ESI+): calcd for $C_{25}H_{28}NO_4S^+$ (M+H) 438.1734, found 438.1730.

**4-(1-(4-(tert-butyl)phenyl)-2-(octylsulfonyl)ethyl)pyridine (41)**

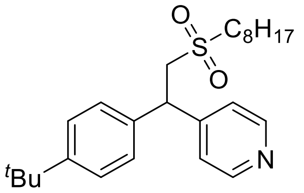


According to the general procedure, 9,10-diphenylanthracene (3.3 mg, 0.01 mmol, 5 mol%), isonicotinonitrile (41.6 mg, 0.4 mmol, 2.0 equiv.), and sodium octane-1-sulfinate (60.0 mg, 0.3 mmol, 1.5 equiv.), 1-(tert-butyl)-4-vinylbenzene (36.6 μL, 0.2 mmol, 1.0 equiv.) and Ammonium chloride (21.4 mg, 0.4 mmol, 2.0 equiv.), the product was isolated by flash chromatography (petroleum ether: ethyl acetate = 1: 2) as a pale-yellow oil (69.7 mg, 84%). $^1$H NMR (400 MHz, $CDCl_3$) δ 8.55 (d, J = 6.0 Hz, 2H), 7.37 (d, J = 8.4 Hz, 2H), 7.25 – 7.13 (m, 4H), 4.77 – 4.47 (m, 1H), 3.68 (ddd, J = 20.7, 14.6, 7.1 Hz, 2H), 2.37 (m, 2H), 1.57 (dd, J = 12.0, 6.7 Hz, 2H), 1.29 (s, 9H), 1.18 (dd, J = 36.0, 12.6 Hz, 10H), 0.87 (t, J = 7.0 Hz, 3H). $^{13}$C NMR (101 MHz, $CDCl_3$) δ 151.16, 150.71, 150.30, 136.82, 127.55, 126.23, 122.70, 57.57, 54.08, 44.83, 34.53, 31.61, 31.24, 29.68, 28.81, 28.22, 22.55, 21.90, 14.02. HRMS (ESI+): calcd for $C_{25}H_{38}NO_2S^+$ (M+H) 416.2618, found 416.2615.

**4-(1-(4-(tert-butyl)phenyl)-2-(cyclohexylsulfonyl)ethyl)pyridine (42)**

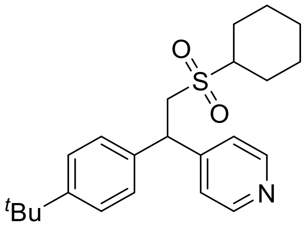

According to the general procedure, 9,10-diphenylanthracene (3.3 mg, 0.01 mmol, 5 mol%), isonicotinonitrile (41.6 mg, 0.4 mmol, 2.0 equiv.), and sodium cyclohexanesulfinate (51.0 mg, 0.3 mmol, 1.5 equiv.), 1-(tert-butyl)-4-vinylbenzene (36.6 μL, 0.2 mmol, 1.0 equiv.) and Ammonium chloride (21.4 mg, 0.4 mmol, 2.0 equiv.), the product was isolated by flash chromatography (petroleum ether: ethyl acetate = 1: 1) as a pale-yellow oil (52.5 mg, 68%). $^1$H NMR (600 MHz, $CDCl_3$) δ 8.55 (d, J = 5.5 Hz, 2H), 7.37 (d, J = 8.4 Hz, 2H), 7.30 – 7.26 (m, 2H), 7.22 (d, J = 8.4 Hz, 2H), 4.66 (m, 1H), 3.75 (dd, J = 14.4, 7.8 Hz, 1H), 3.59 (dd, J = 14.4, 6.3 Hz, 1H), 2.09 – 2.00 (m, 2H), 1.84 (m, 2H), 1.77 (d, J = 13.5 Hz, 1H), 1.59 (d, J = 13.3 Hz, 1H), 1.47 – 1.40 (m, 2H), 1.30 (s, 9H), 1.14 – 1.07 (m, 1H), 0.97 m, 1H), 0.90 – 0.85 (m, 1H). $^{13}$C NMR (101 MHz, $CDCl_3$) δ 151.79, 150.95, 149.28, 136.90, 127.49, 126.07, 123.00, 61.36, 54.08, 44.38, 34.42, 31.16, 25.37, 24.91, 24.77, 24.72, 23.77. HRMS (ESI+): calcd for $C_{23}H_{32}NO_2S^+$ (M+H) 386.2148, found 386.2144.

**4-(1-(4-(tert-butyl)phenyl)-2-(cyclopropylsulfonyl)ethyl)pyridine (43)**

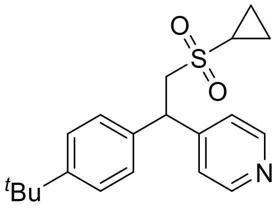


According to the general procedure, 9,10-diphenylanthracene (3.3 mg, 0.01 mmol, 5 mol%), isonicotinonitrile (41.6 mg, 0.4 mmol, 2.0 equiv.), and sodium cyclopropanesulfinate (38.4 mg, 0.3 mmol, 1.5 equiv.), 1-(tert-butyl)-4-vinylbenzene (36.6 μL, 0.2 mmol, 1.0 equiv.) and Ammonium chloride (21.4 mg, 0.4 mmol, 2.0 equiv.), the product was isolated by flash chromatography (petroleum ether: ethyl acetate = 1: 2) as a pale-yellow oil (63.2 mg, 92%). $^1$H NMR (400 MHz, $CDCl_3$) δ 8.47 (d, J = 5.5 Hz, 2H), 7.28 (d, J = 8.4 Hz, 2H), 7.19 (d, J = 5.5 Hz, 2H), 7.14 (d, J = 8.4 Hz, 2H), 4.59 (t, J = 7.1 Hz, 1H), 3.72 (d, J = 7.1 Hz, 2H), 1.72 (tt, J = 8.0, 4.8 Hz,

1H), 1.20 (s, 9H), 1.10 – 0.97 (m, 2H), 0.73 – 0.61 (m, 2H). $^{13}$C NMR (101 MHz, $CDCl_3$) δ 150.97, 150.81, 149.98, 137.08, 127.32, 126.02, 122.88, 58.75, 44.85, 34.42, 31.15, 30.71, 5.24, 5.15. HRMS (ESI+): calcd for $C_{20}H_{26}NO_2S^+$ (M+H) 344.1679, found 344.1675.

## 6. Mechanistic Studies

### 6.1. Radical clock experiments

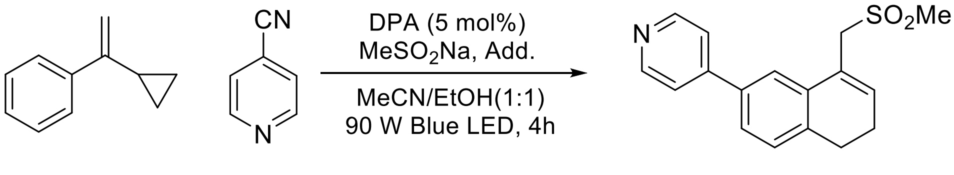


**45**, 47% yield

To a flame-dried 10 mL reaction vial was charged with 9,10-diphenylanthracene (3.3 mg, 0.01 mmol, 5 mol%), isonicotinonitrile (41.6 mg, 0.4 mmol, 2.0 equiv.), and Sodium methyl sulfinate (34 mg, 0.3 mmol, 1.5 equiv.), and Ammonium chloride (21.4 mg, 0.4 mmol, 2.0 equiv.). The vial was capped. MeCN/EtOH (1:1) [0.025 M] was added via a syringe. It was bubbled with nitrogen for 15 minutes, followed by the addition of (1-cyclopropylvinyl)benzene (30.8 μL, 0.2 mmol, 1.0 equiv.). The reaction mixture was then irradiated with a 90 W Blue LED lamp for 4 h at room temperature. After reaction completed, the mixture was evaporated on rotary evaporator. Then, the residue was dilute with ethyl acetate and wash with $H_2O$. The combined organic layers were dried with $MgSO_4$, filtered, and concentrated in vacuo. The product was isolated by flash chromatography (petroleum ether: acetone = 1: 1) as a white solid (28.1 mg, 47 %).

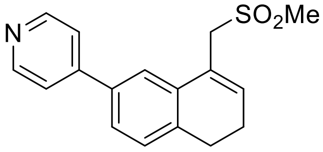


**4-(8-((methylsulfonyl)methyl)-5,6-dihydronaphthalen-2-yl)pyridine (45)**

$^1$H NMR (400 MHz, $CDCl_3$) δ 8.66 (d, J = 3.5 Hz, 2H), 7.68 (s, 1H), 7.53 (d, J = 5.4 Hz, 2H), 7.47 (d, J = 7.7 Hz, 1H), 7.28 (d, J = 8.5 Hz, 1H), 6.37 (t, J = 4.5 Hz, 1H),

4.24 (s, 2H), 2.91 (s, 3H), 2.88 (t, J = 8.1 Hz, 2H), 2.49 – 2.41 (m, 2H). $^{13}C$ NMR (101 MHz, $CDCl_3$) δ 149.99, 148.43, 137.27, 136.44, 135.89, 133.17, 128.67, 126.34, 125.85, 122.40, 121.63, 58.51, 39.80, 27.44, 23.35. HRMS (ESI+): calcd for $C_{17}H_{18}NO_2S^+$ (M+H) 300.1053, found 300.1049.

**6.2. Stern-Volmer fluorescence quenching studies.**

The emission intensity at 428 nm was collected with excited wavelength of 373 nm in MeCN/EtOH using a Shimadzu RF-5301pc spectrofluorophotometer. The concentration of DPA is 1 X $10^{-5}$ M. After degassing the sample with a stream of argon for 30 minutes, plots were constructed according to the Ster-Volmer equation $I_0$ / I=I +kq $t_0$ [4].

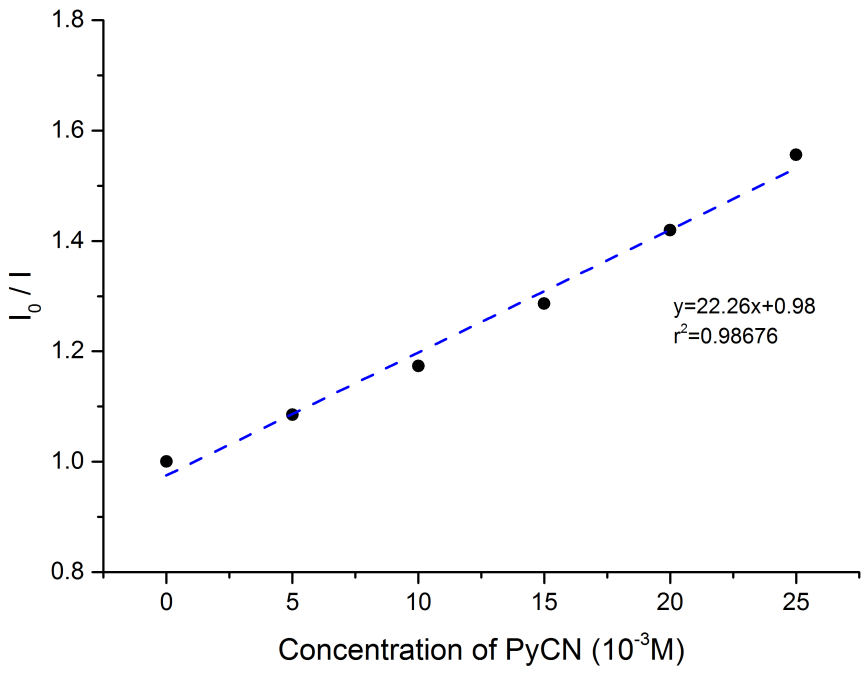


**Figure S1. DPA emission quenching with 4-cyanopyridine**

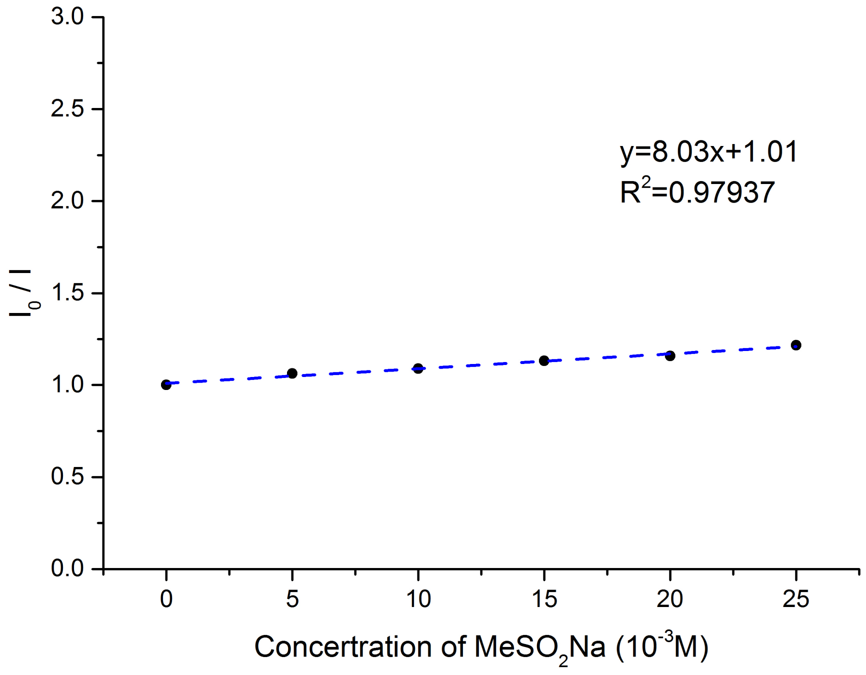


**Figure S2. DPA emission quenching with $MeSO_2Na$**

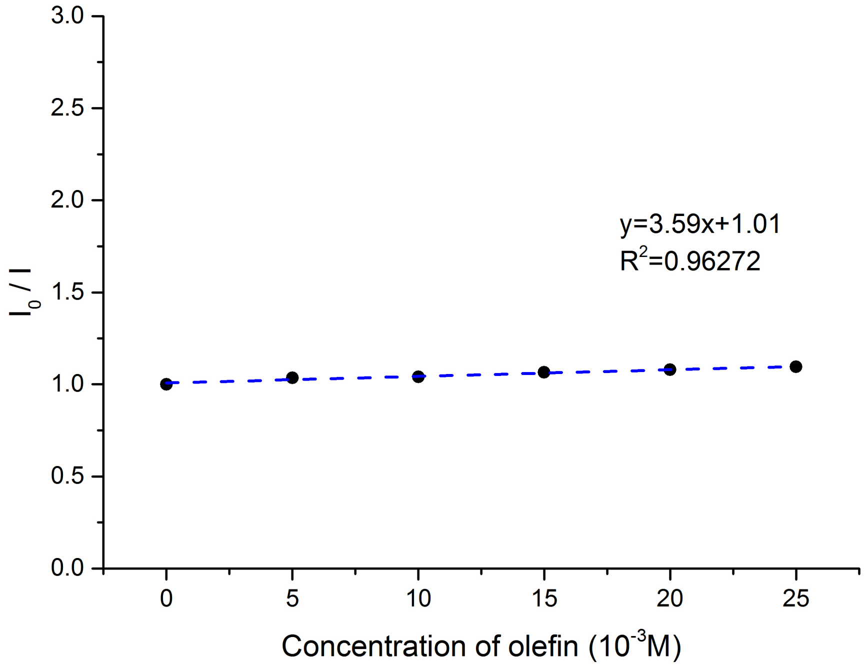


**Figure S3. DPA emission quenching with 4-tert-Butylstyrene**

# 7. NMR Spectra

**3;** $^{1}$H NMR (400 MHz, $CDCl_3$)

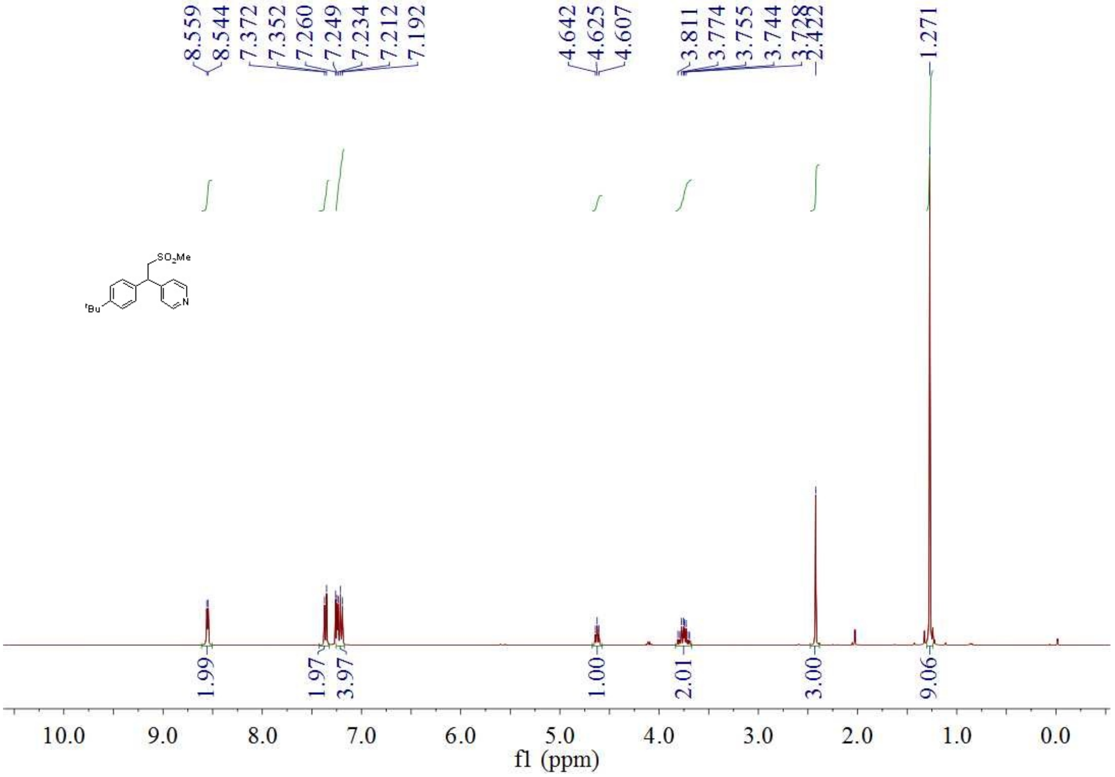


**3;** $^{13}$C NMR (101 MHz, $CDCl_3$)

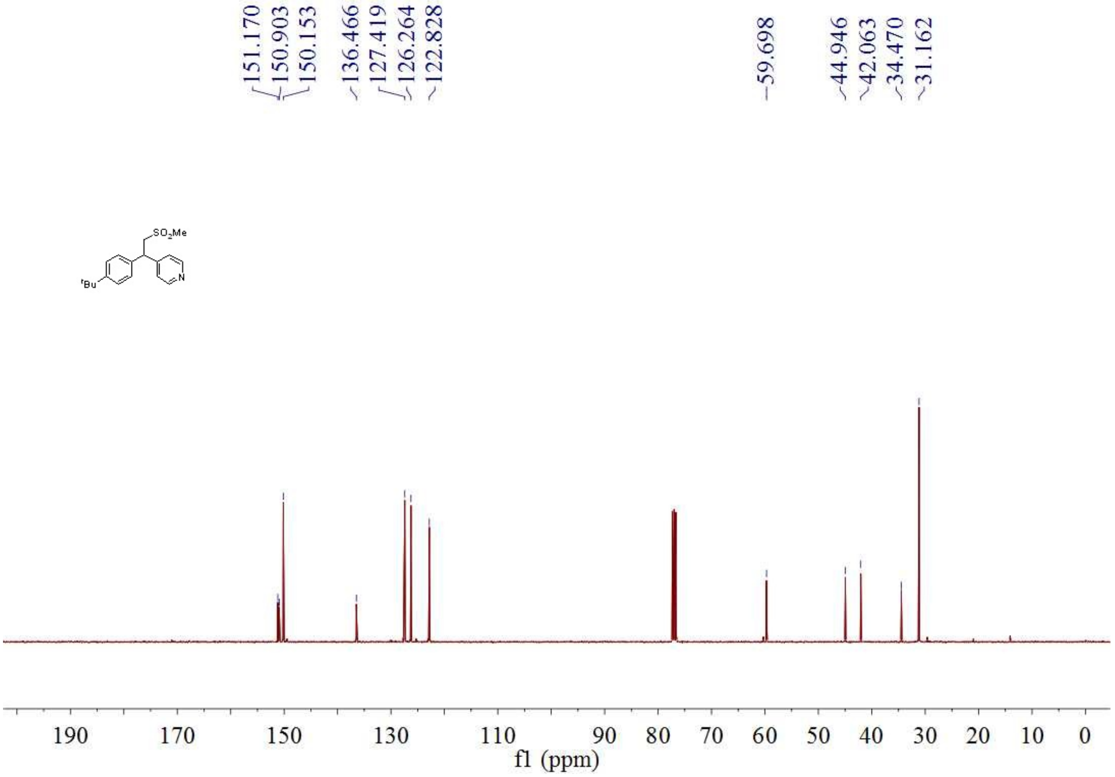

**4;** $^{1}$H NMR (400 MHz, $CDCl_3$)

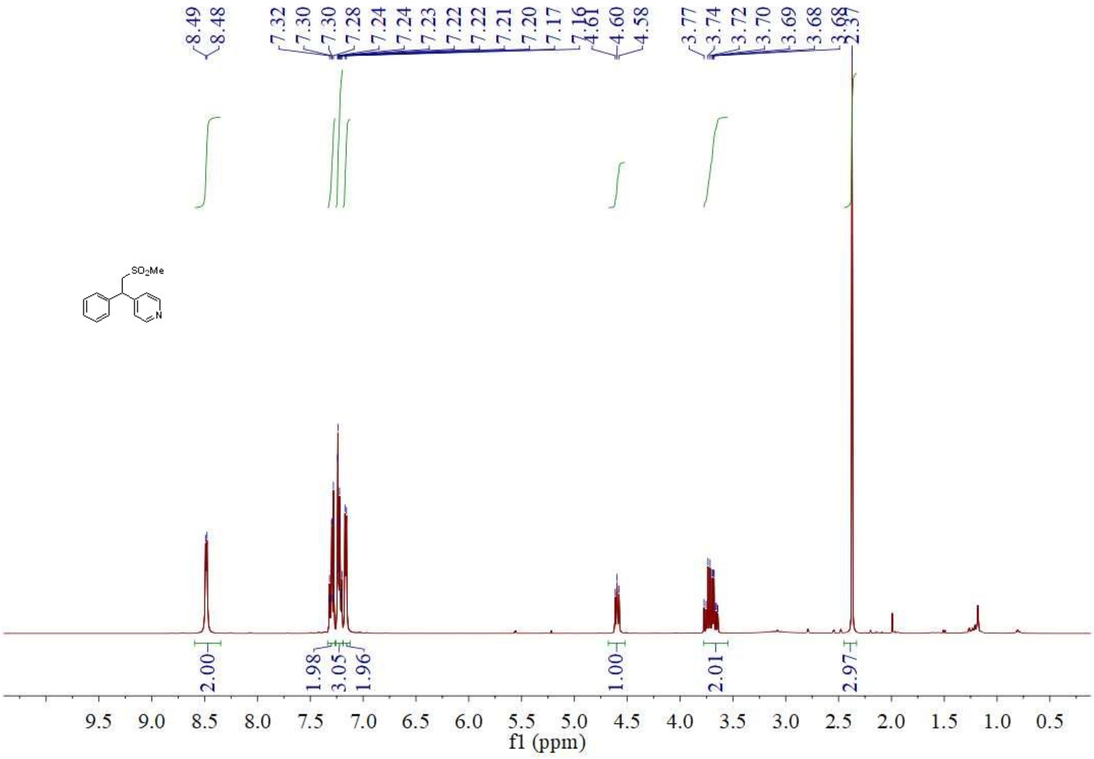


**4;** $^{13}$C NMR (101 MHz, $CDCl_3$)

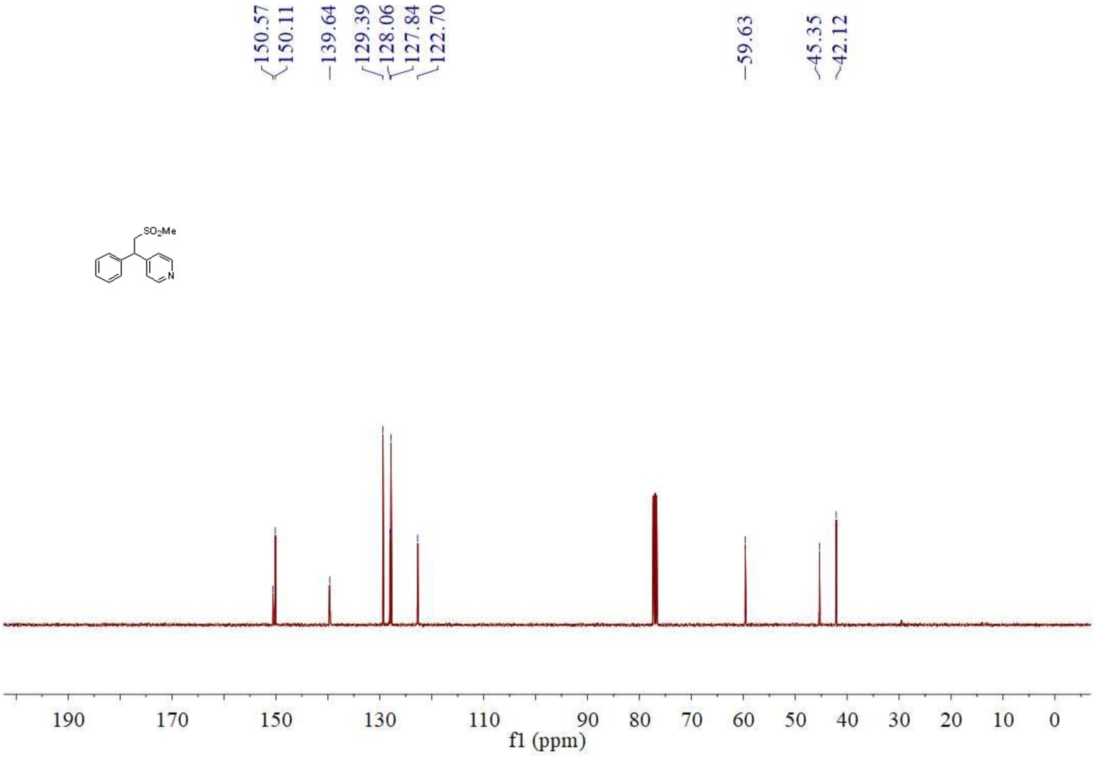

**5;** $^{1}$H NMR (400 MHz, $CDCl_3$)

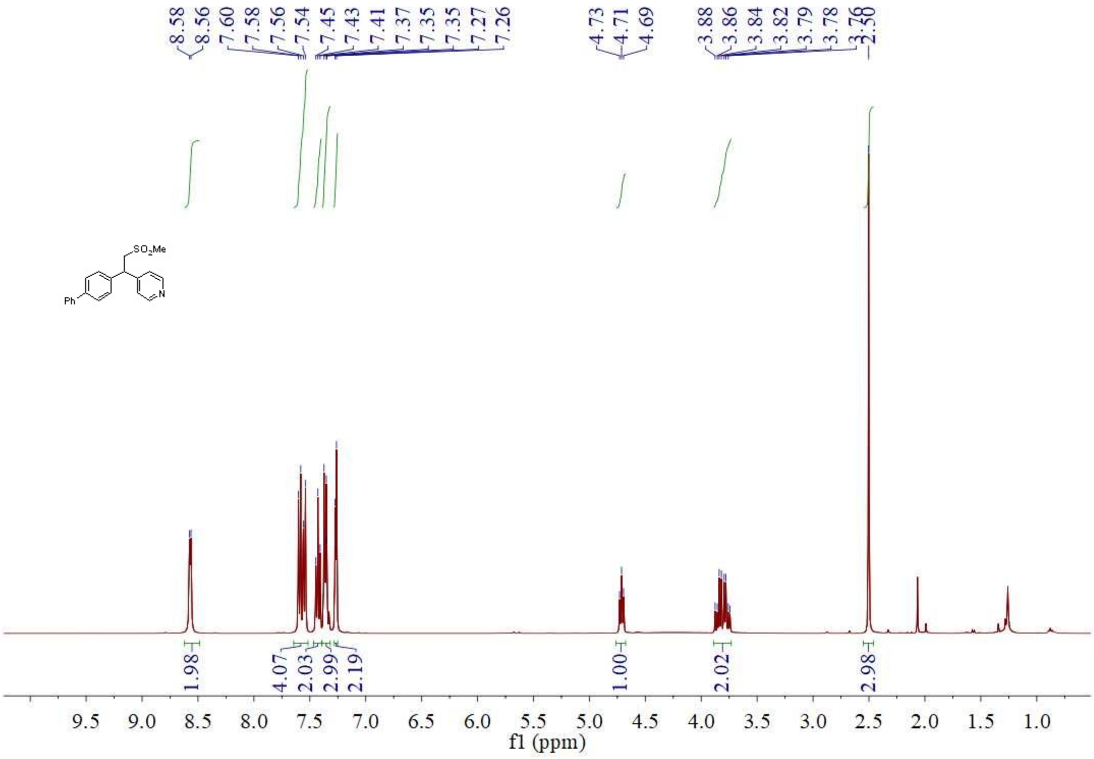

**5;** $^{13}$C NMR (101 MHz, $CDCl_3$)

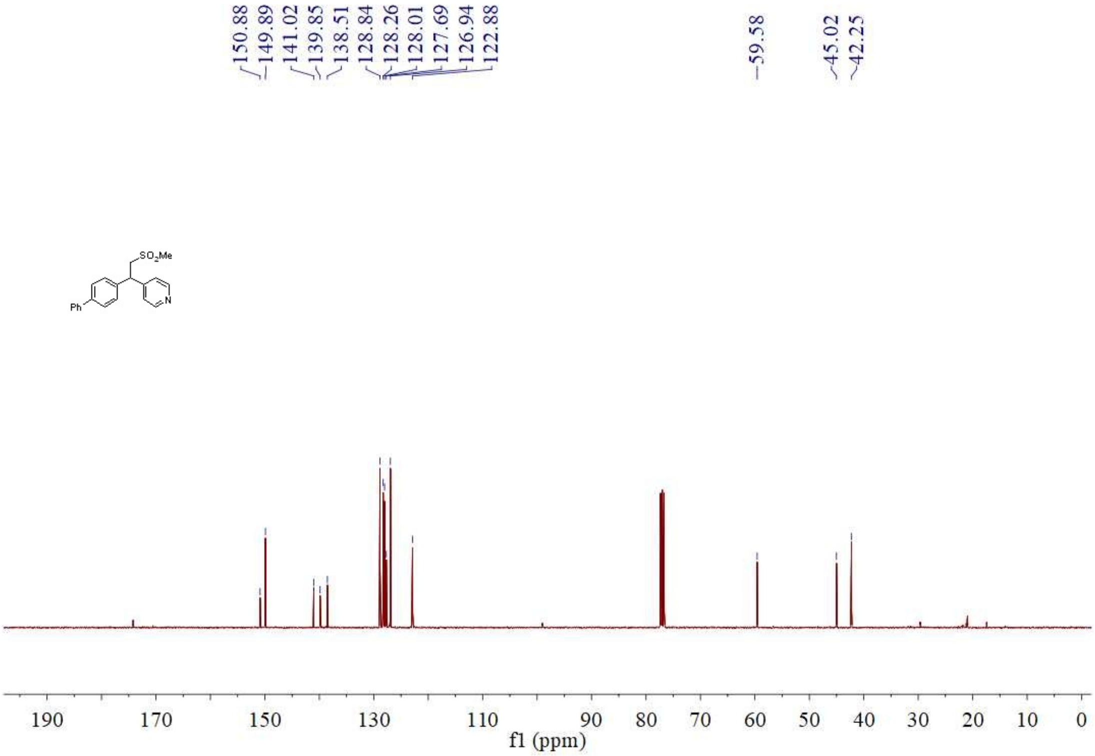

**6;** $^{1}$H NMR (400 MHz, $CDCl_3$)

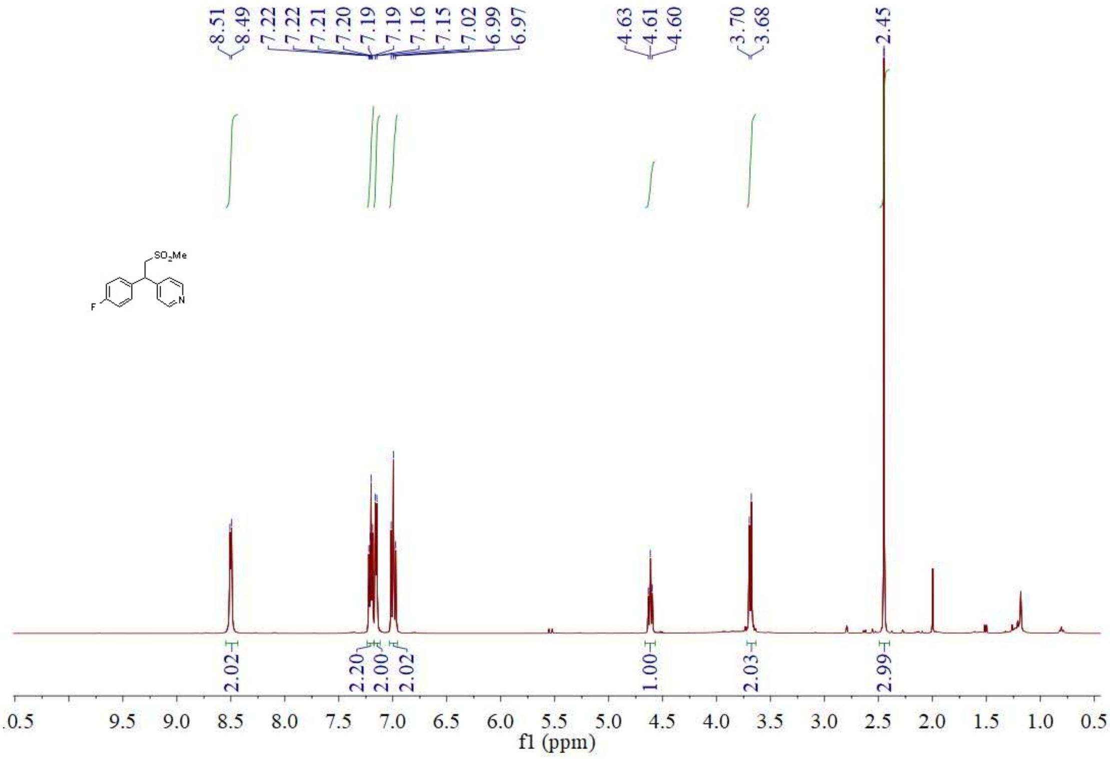


**6;** $^{13}$C NMR (101 MHz, $CDCl_3$)

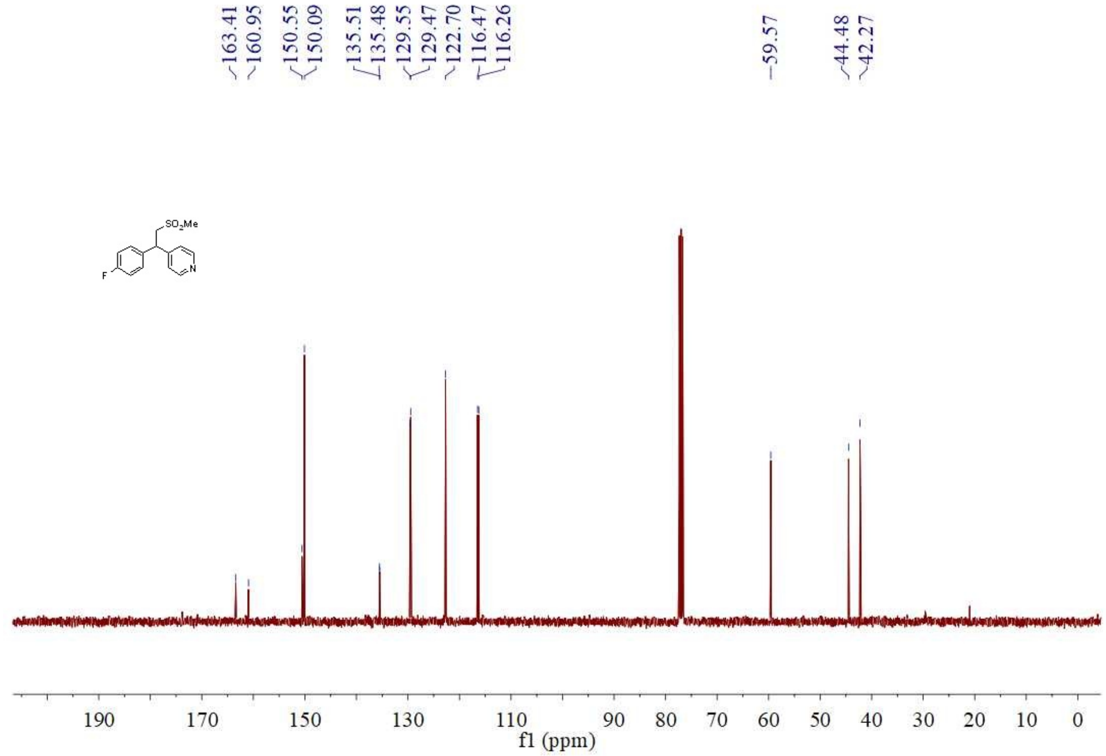

**6;** $^{19}$F NMR (377 MHz, $CDCl_3$)

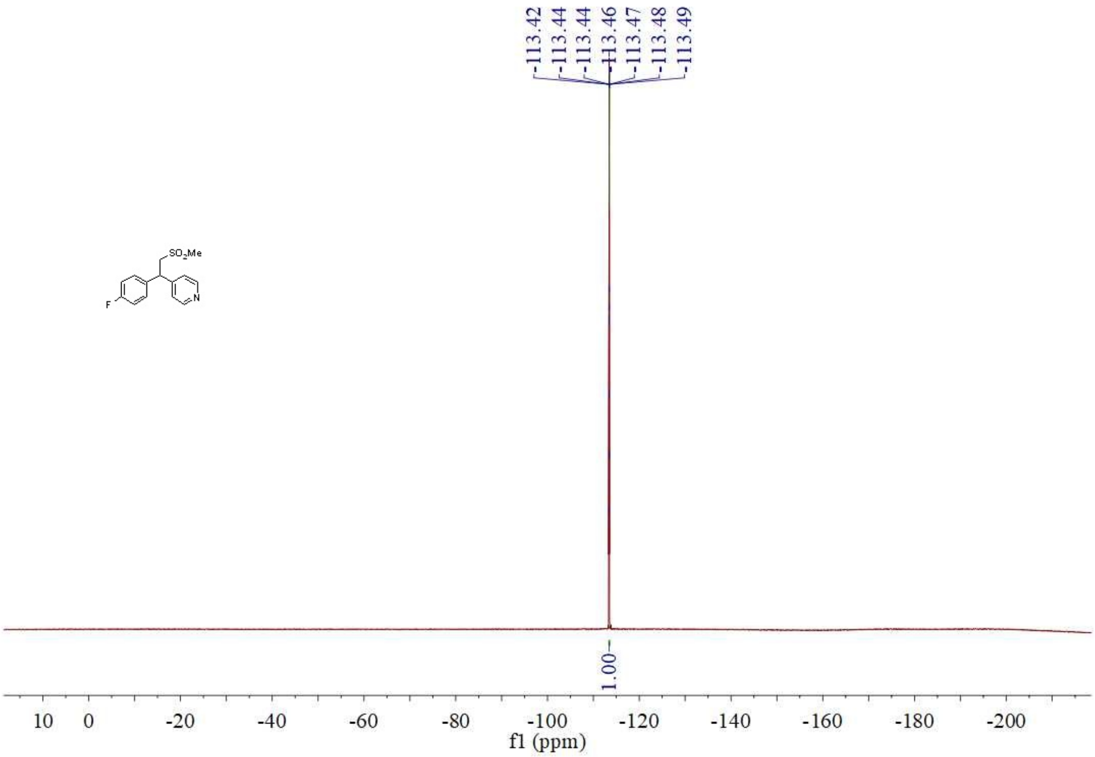


**7;** $^{1}$H NMR (600 MHz, $CDCl_3$)

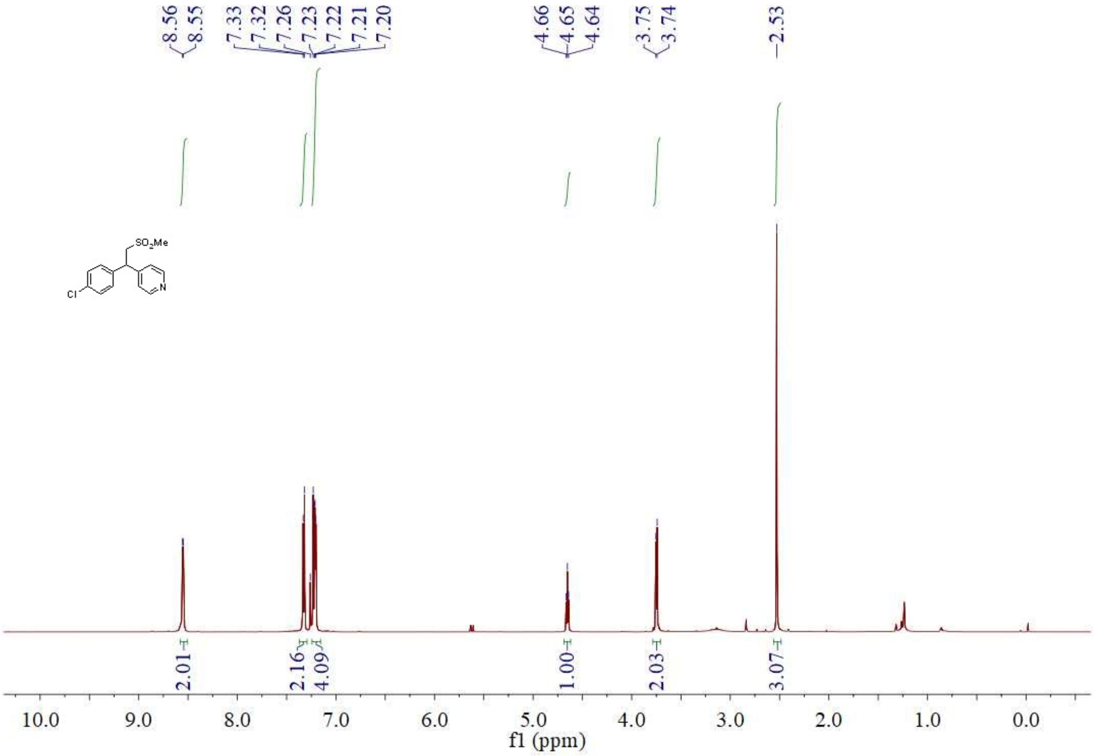

**7**; $^{13}$C NMR (101 MHz, $CDCl_3$)

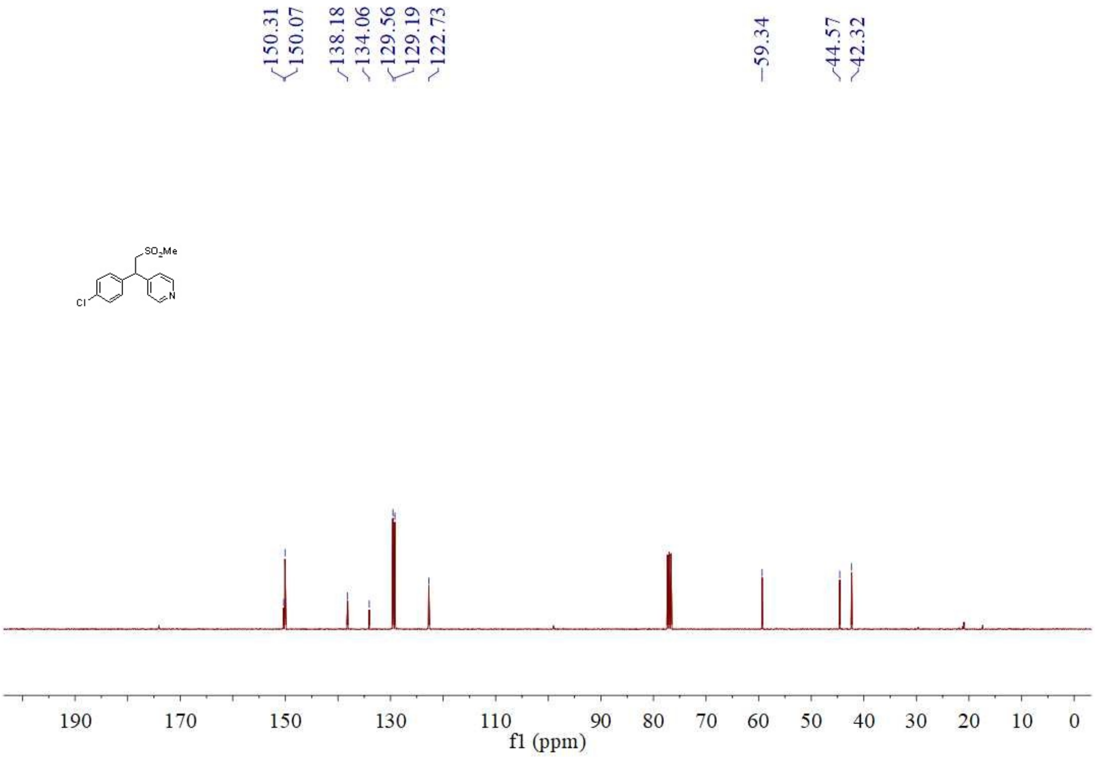


**8**; $^{1}$H NMR (400 MHz, $CDCl_3$)

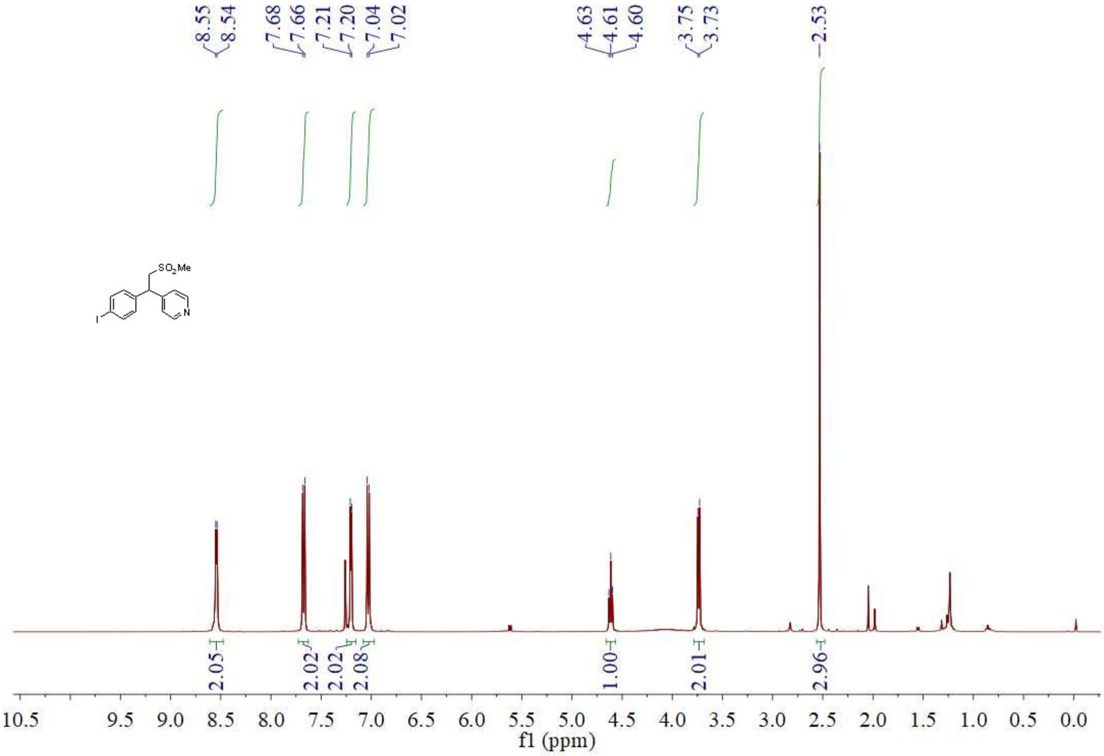

**8;** $^{13}$C NMR (101 MHz, $CDCl_3$)

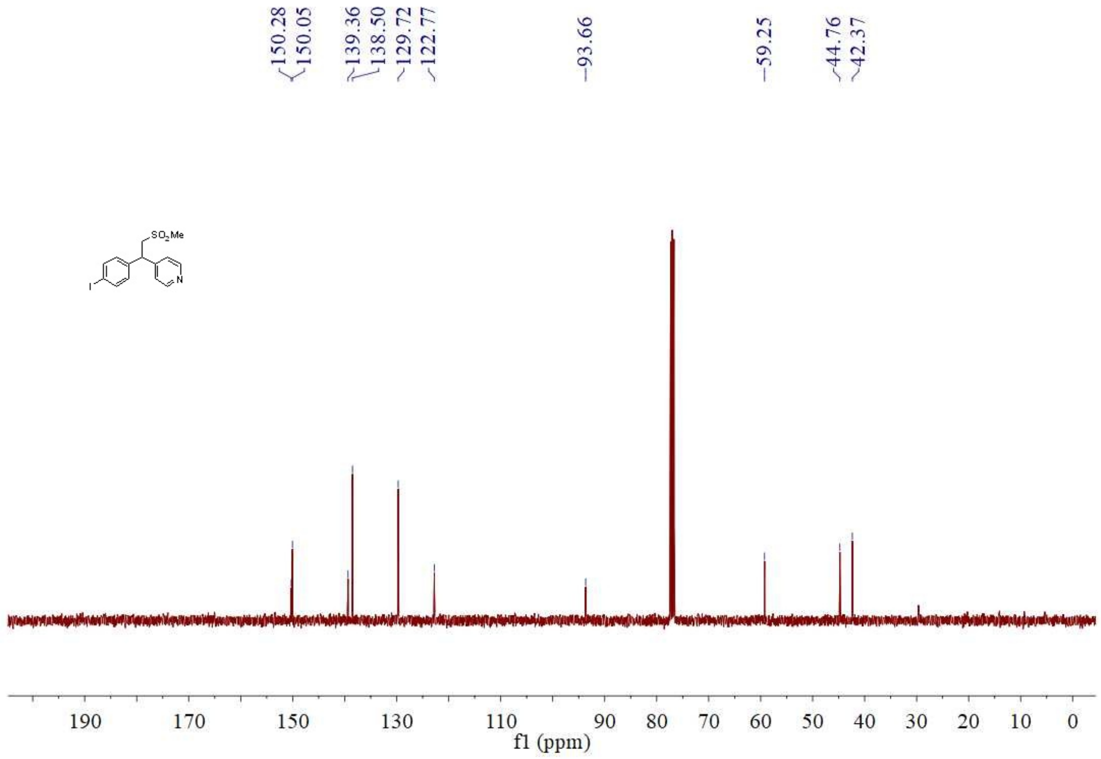


**9;** $^{1}$H NMR (400 MHz, $CDCl_3$)

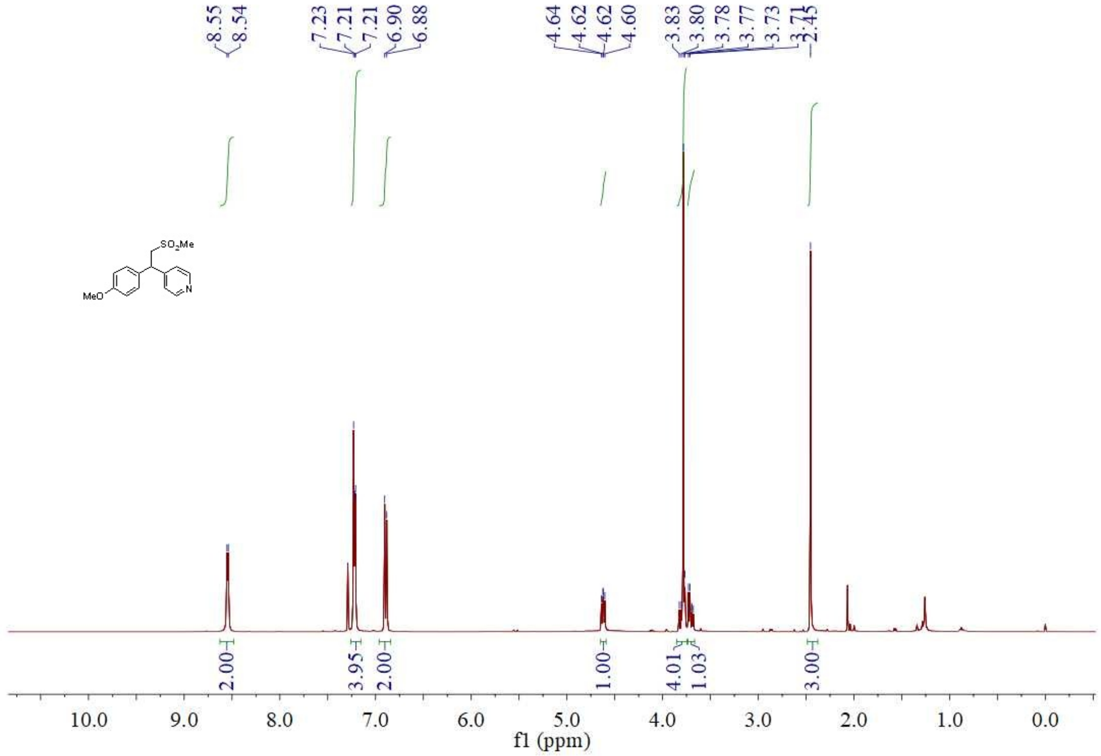

**9;** $^{13}$C NMR (101 MHz, $CDCl_3$)

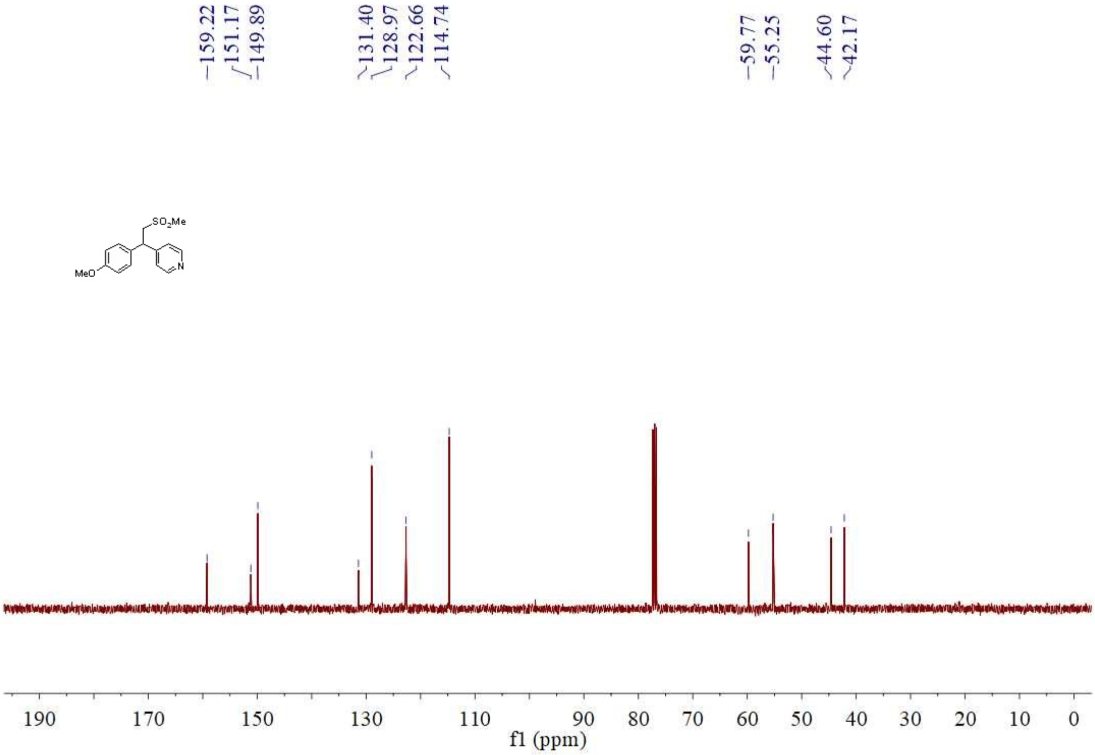


**10;** $^{1}$H NMR (400 MHz, $CDCl_3$)

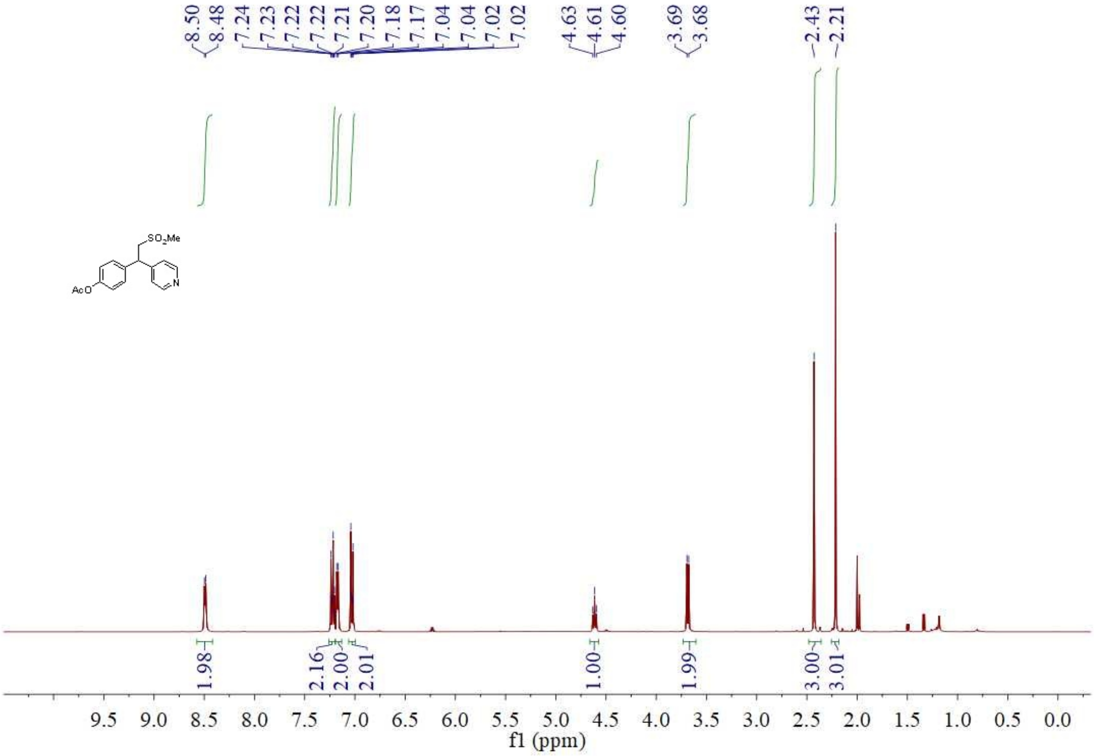

**10;** $^{13}$C NMR (101 MHz, $CDCl_3$)

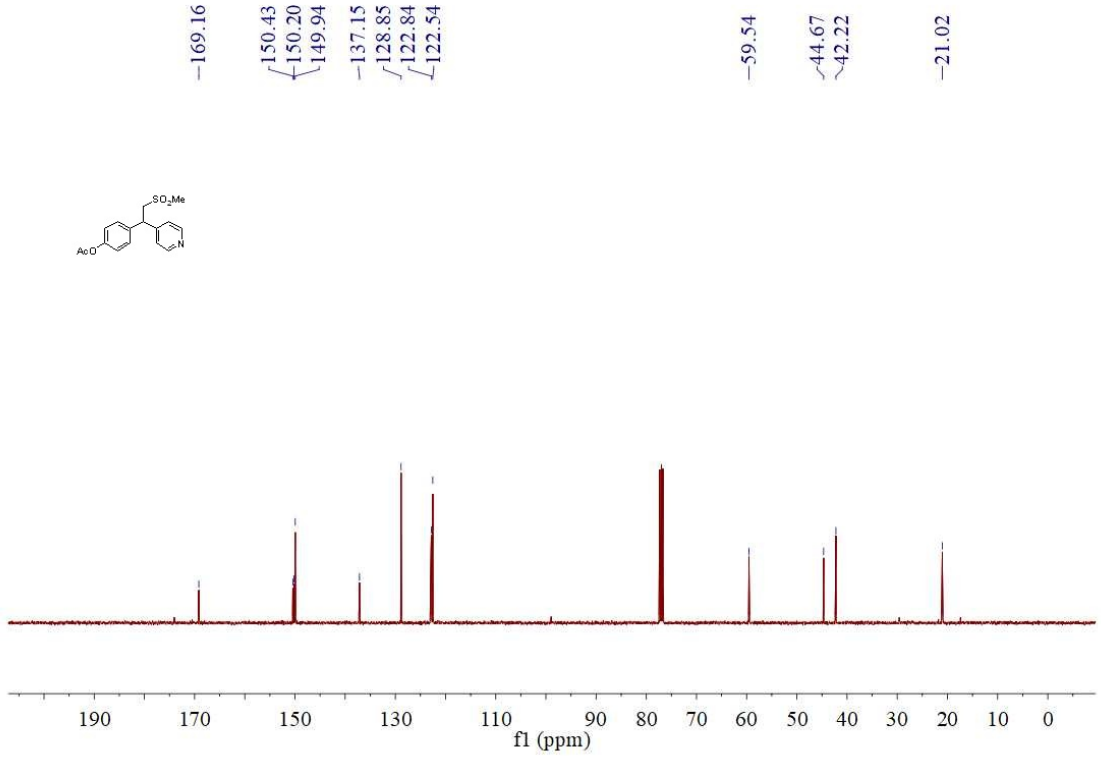


**11;** $^{1}$H NMR (400 MHz, $CDCl_3$)

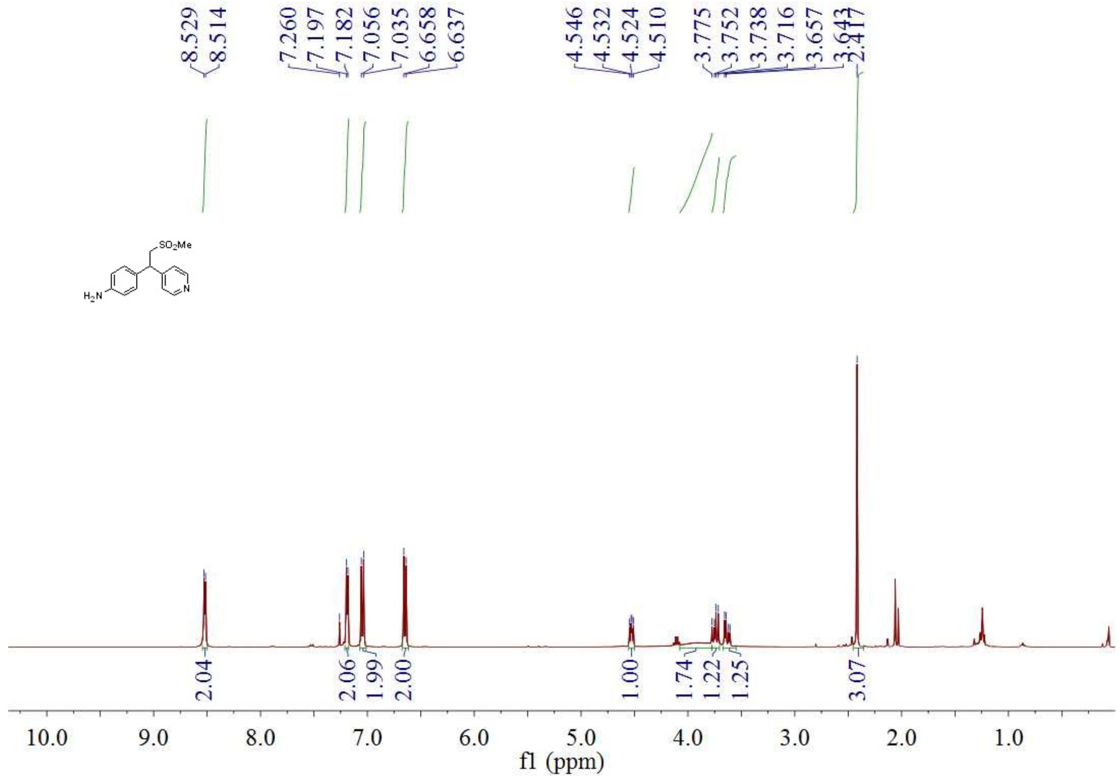

**11;** $^{13}$C NMR (101 MHz, $CDCl_3$)

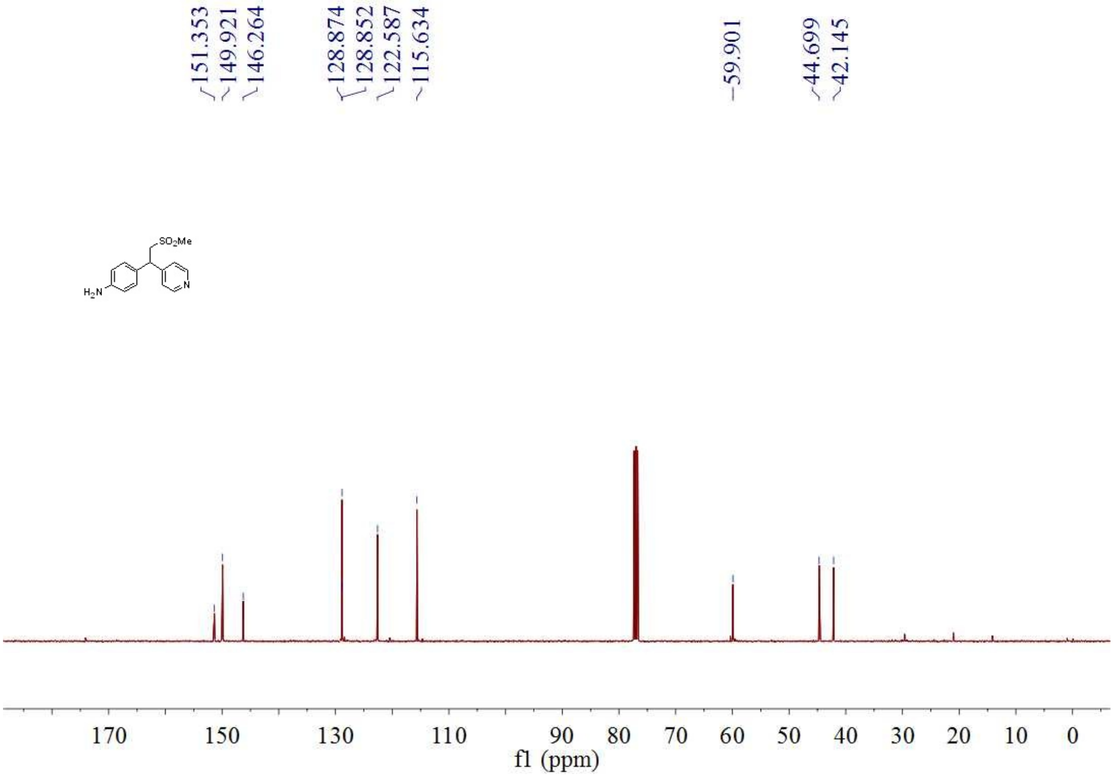


**12;** $^{1}$H NMR (400 MHz, $CDCl_3$)

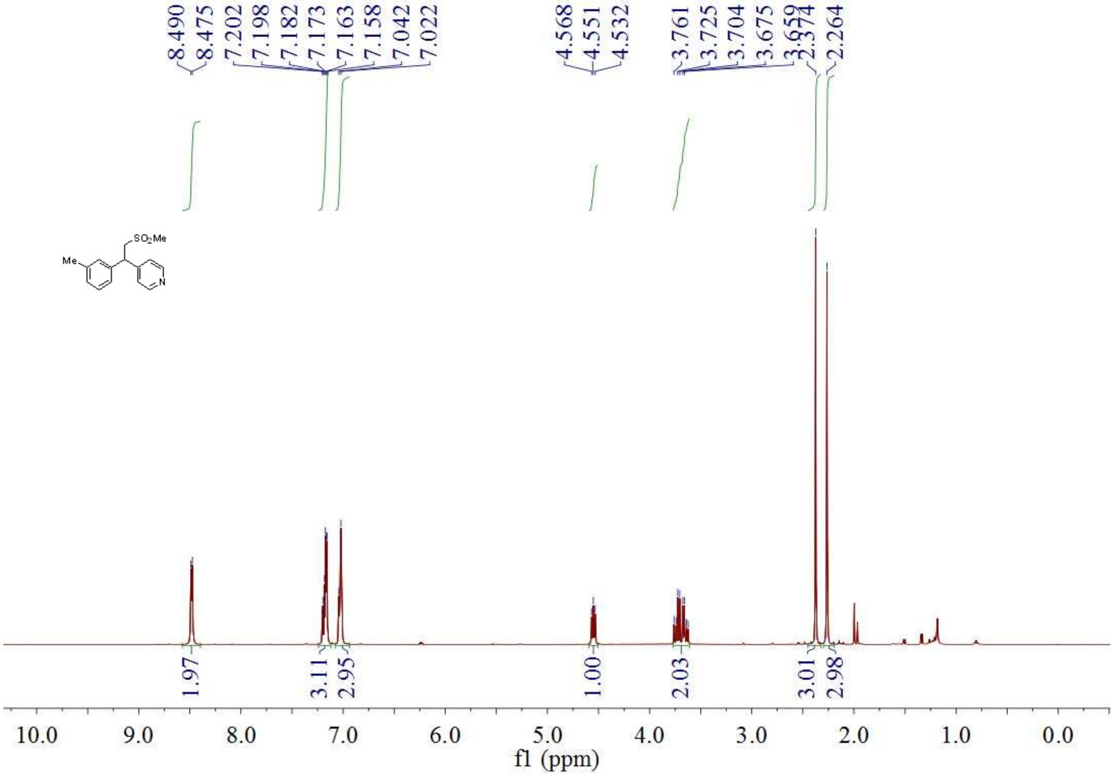

**12;** $^{13}$C NMR (101 MHz, $CDCl_3$)

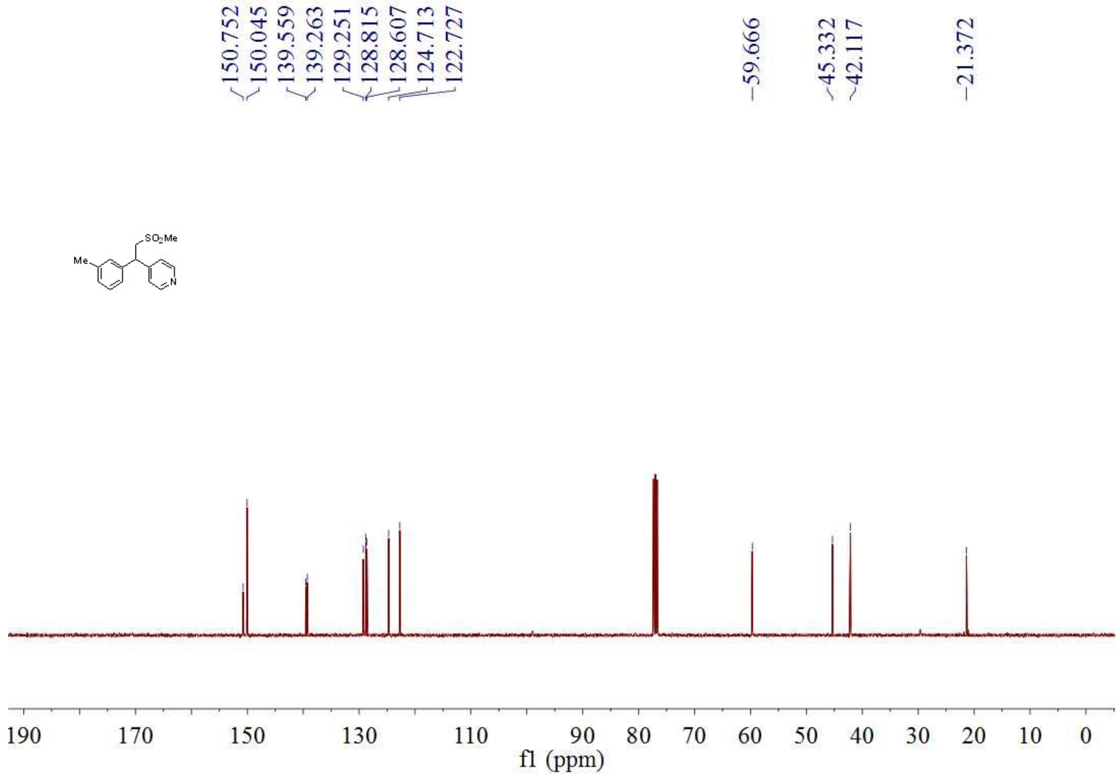


**13;** $^{1}$H NMR (400 MHz, $CDCl_3$)

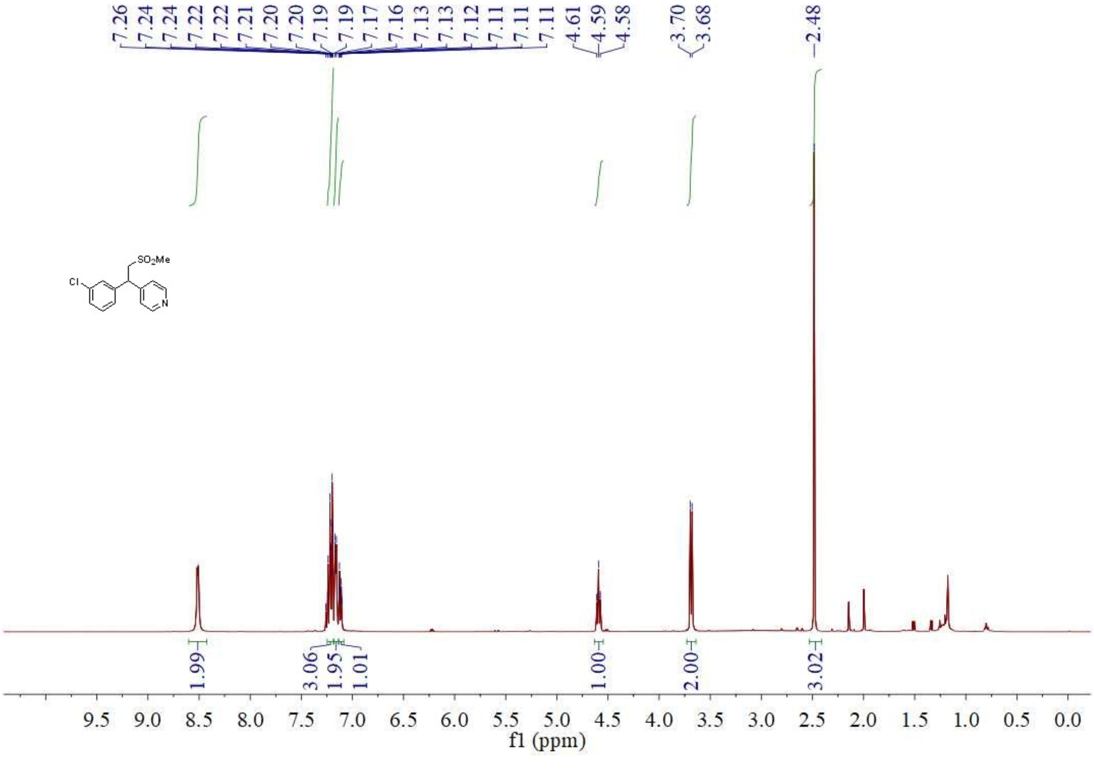

**13;** $^{13}$C NMR (101 MHz, $CDCl_3$)

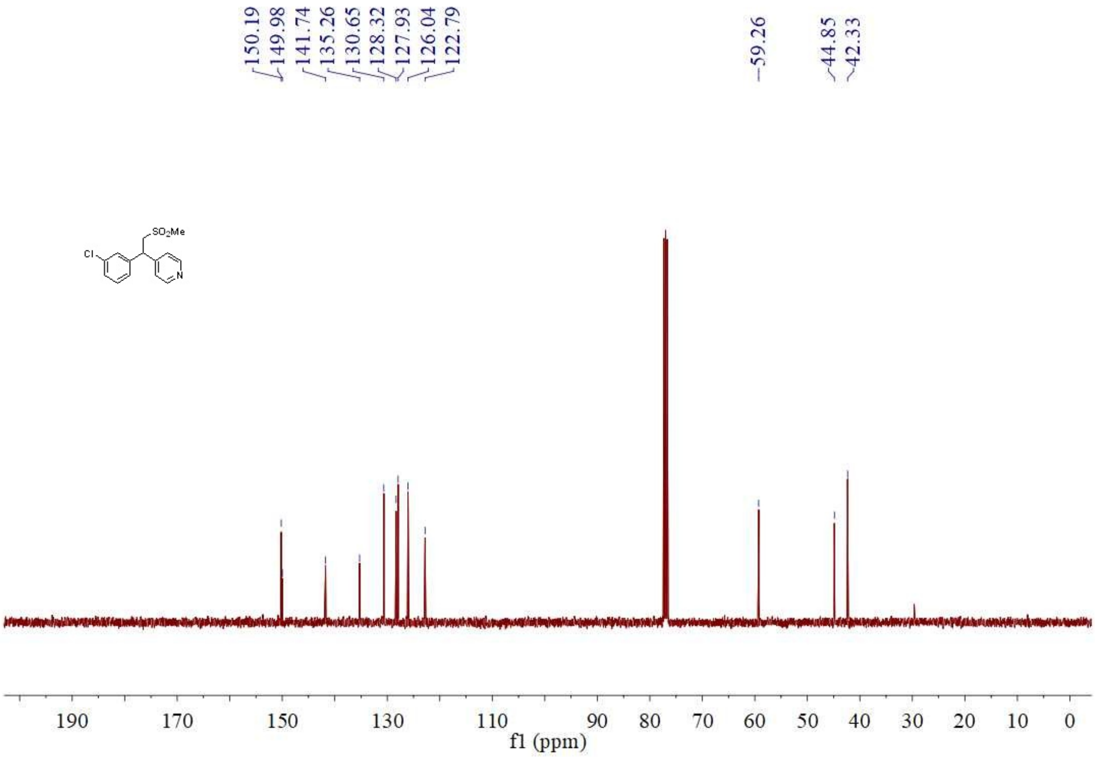


**14;** $^{1}$H NMR (400 MHz, $CDCl_3$)

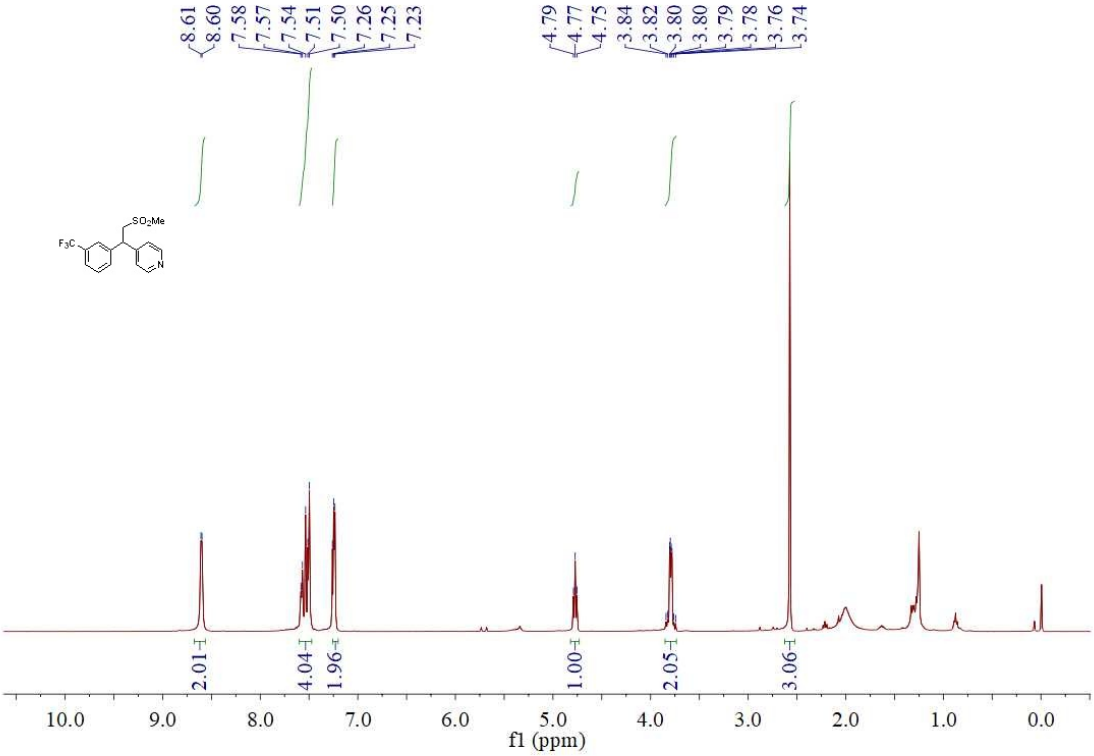

**14;** $^{13}$C NMR (101 MHz, $CDCl_3$)

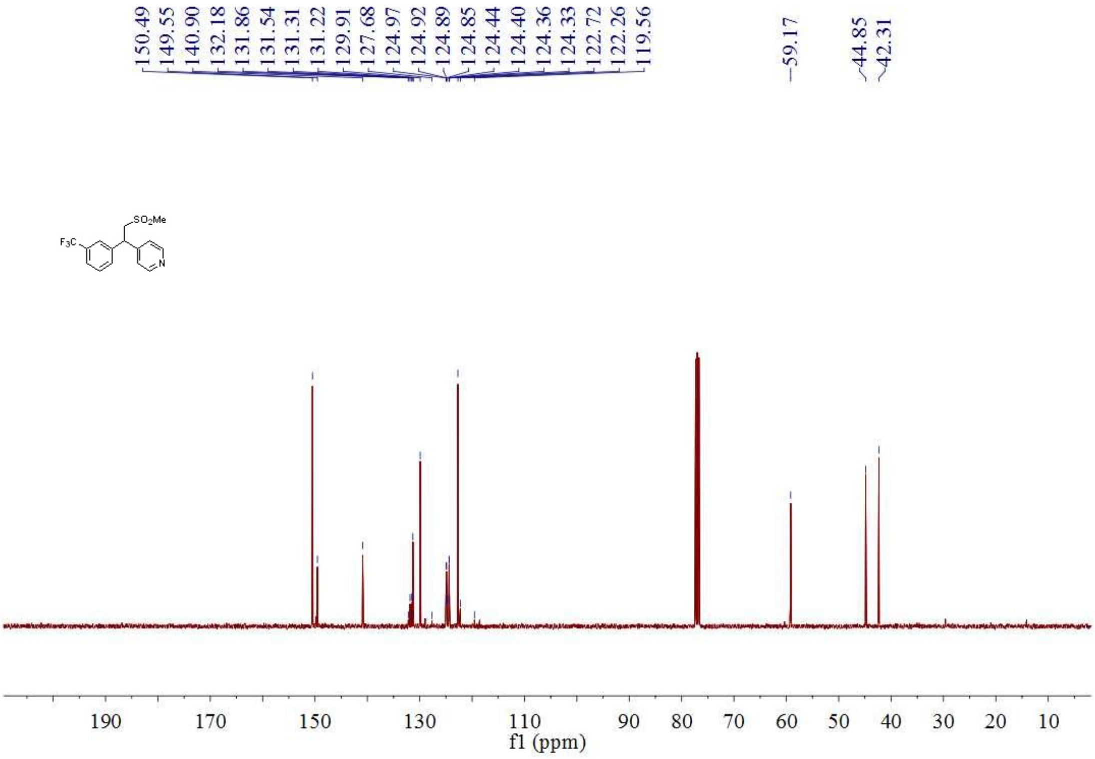


**14;** $^{19}$F NMR (377 MHz, $CDCl_3$)

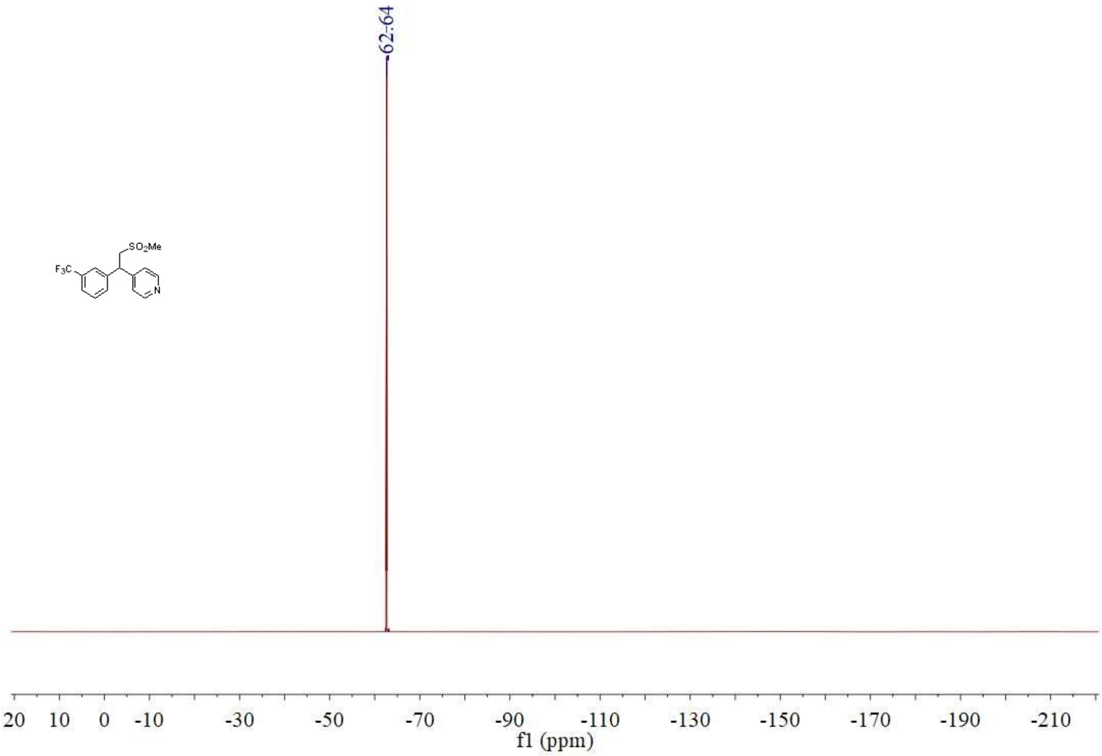

**15;** $^{1}$H NMR (600 MHz, $CDCl_3$)

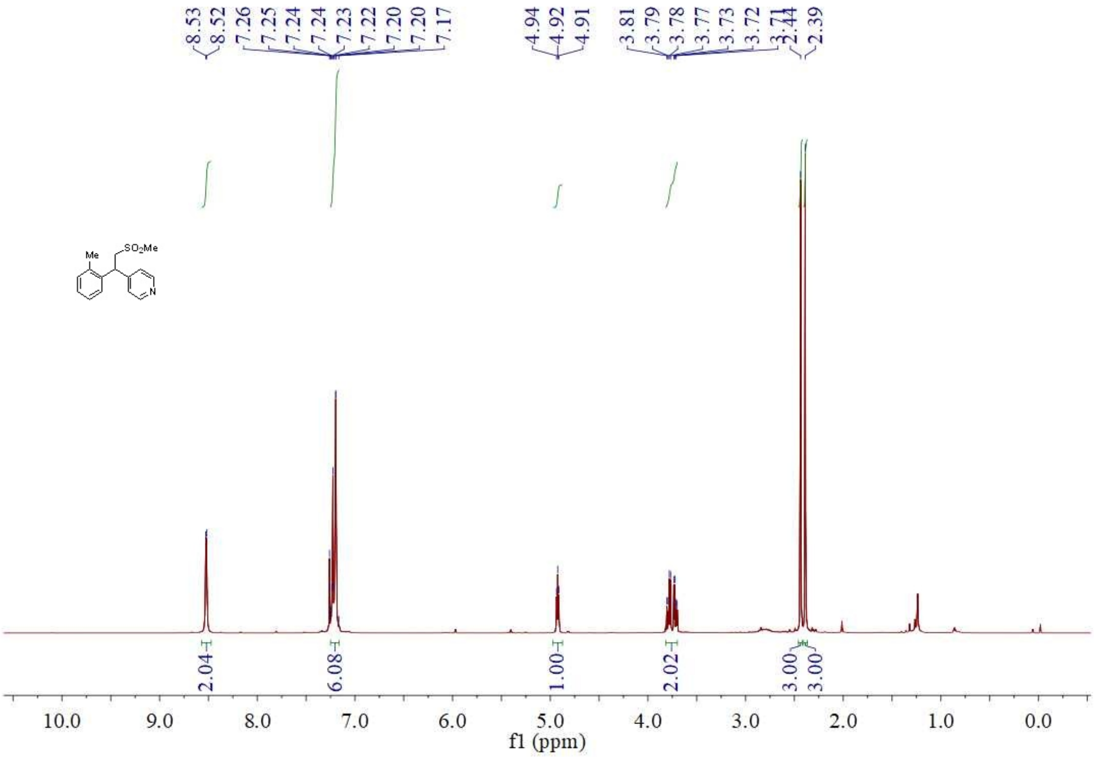


**15;** $^{13}$C NMR (101 MHz, $CDCl_3$)

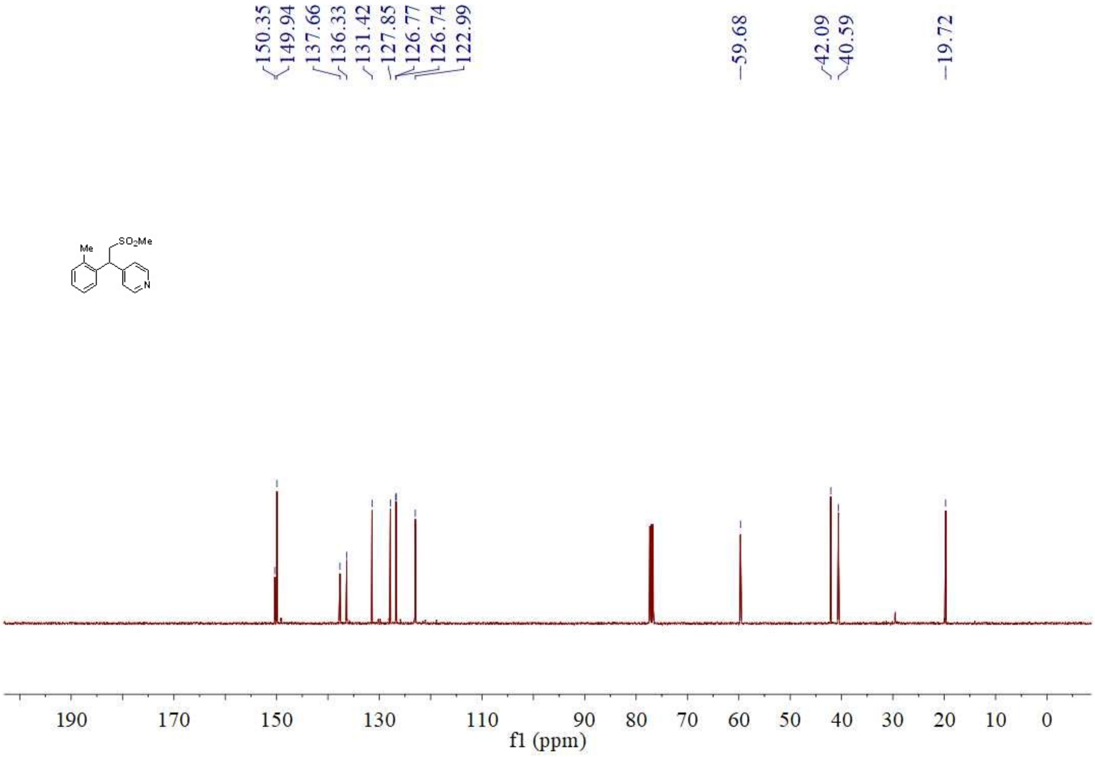

**16;** $^{1}$H NMR (400 MHz, $CDCl_3$)

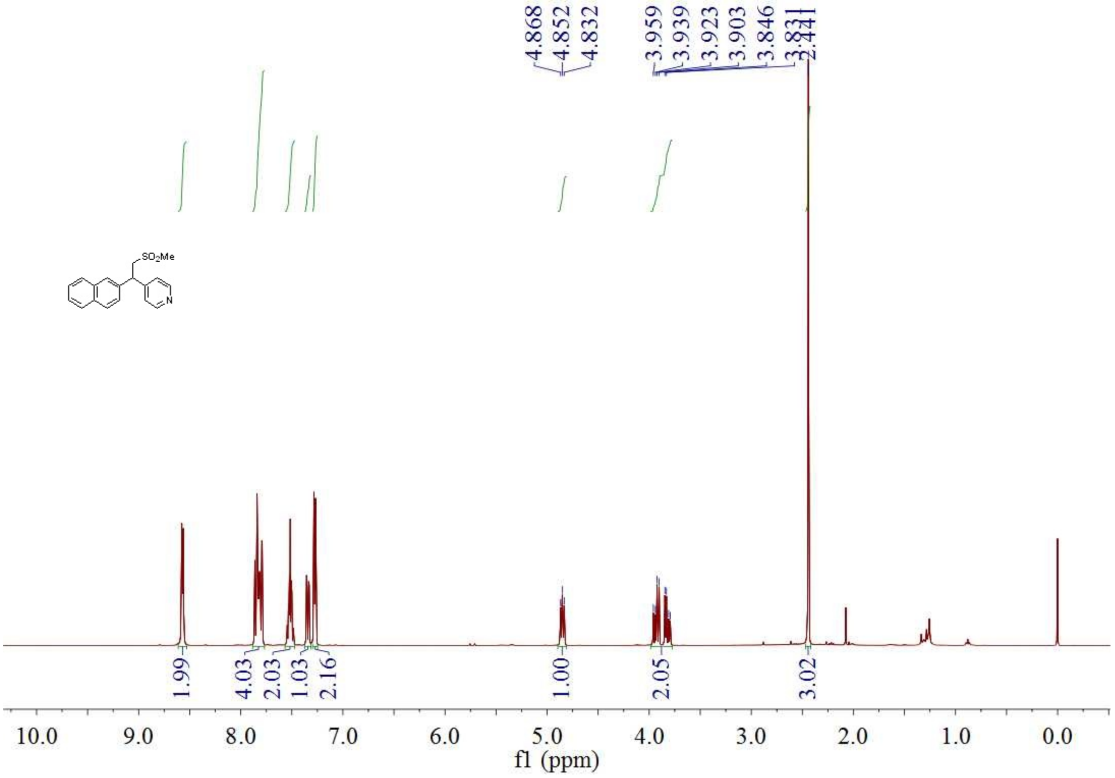


**16;** $^{13}$C NMR (101 MHz, $CDCl_3$)

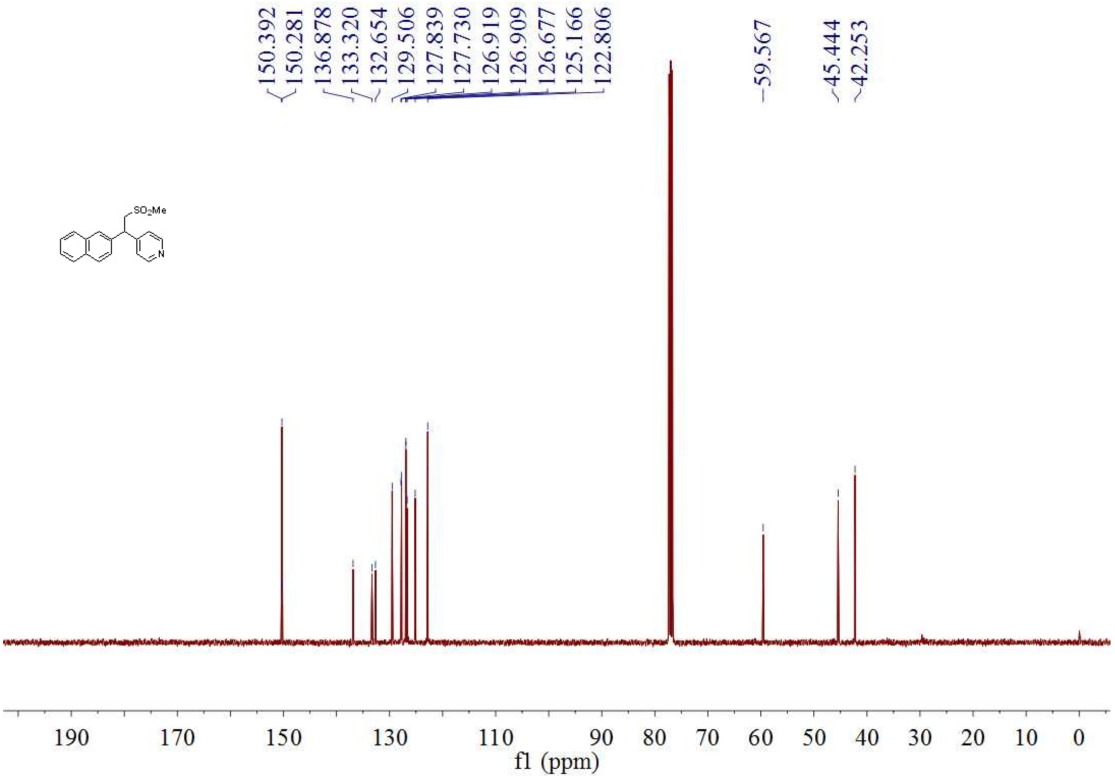

**17;** $^{1}$H NMR (400 MHz, $CDCl_3$)

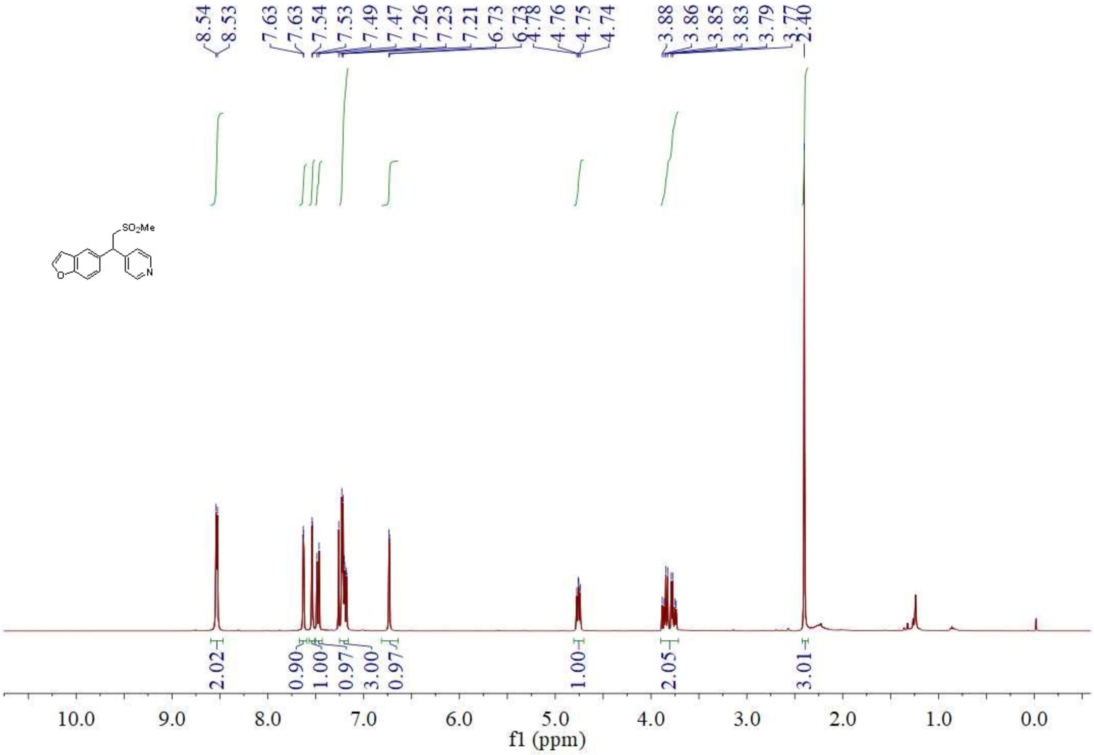


**17;** $^{13}$C NMR (101 MHz, $CDCl_3$)

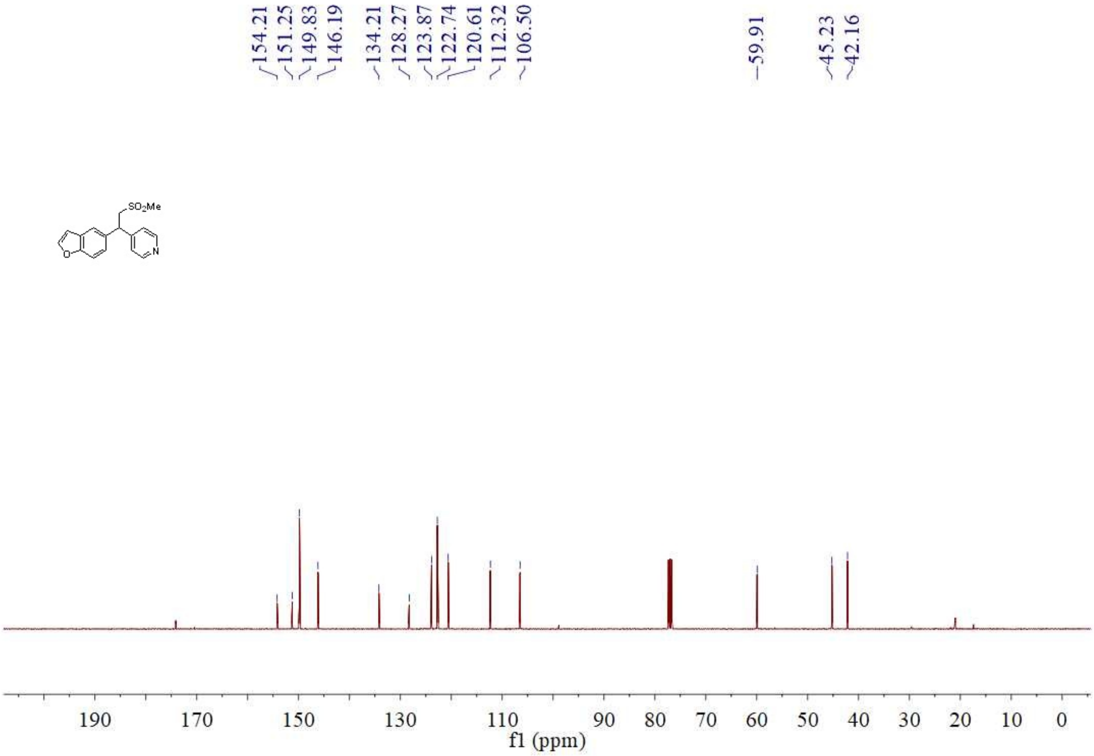

**18;** $^{1}$H NMR (400 MHz, $CDCl_3$)

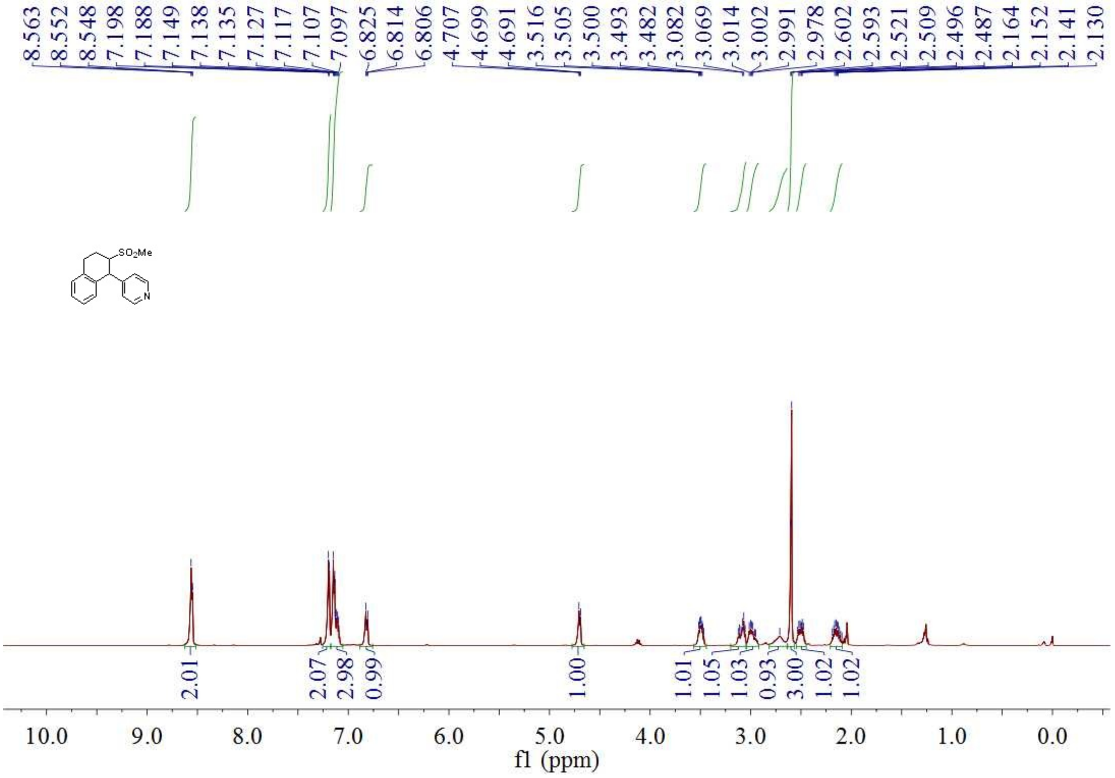


**18;** $^{13}$C NMR (101 MHz, $CDCl_3$)

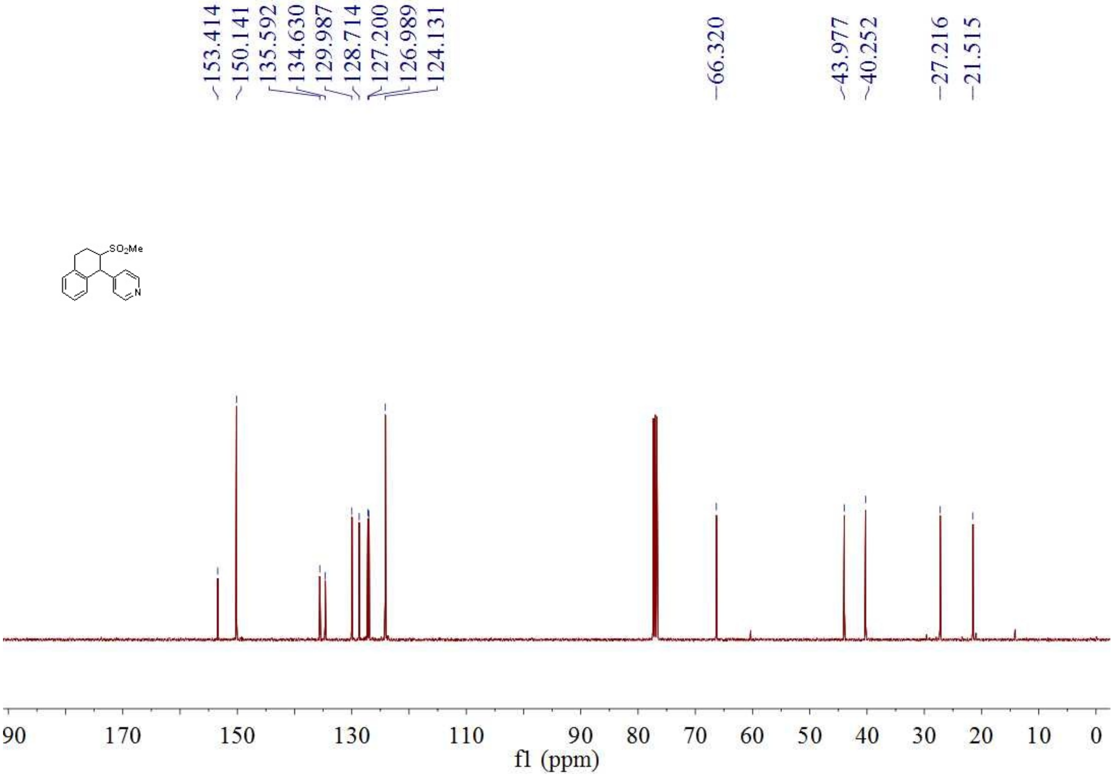

**19;** $^{1}$H NMR (600 MHz, $CDCl_3$)

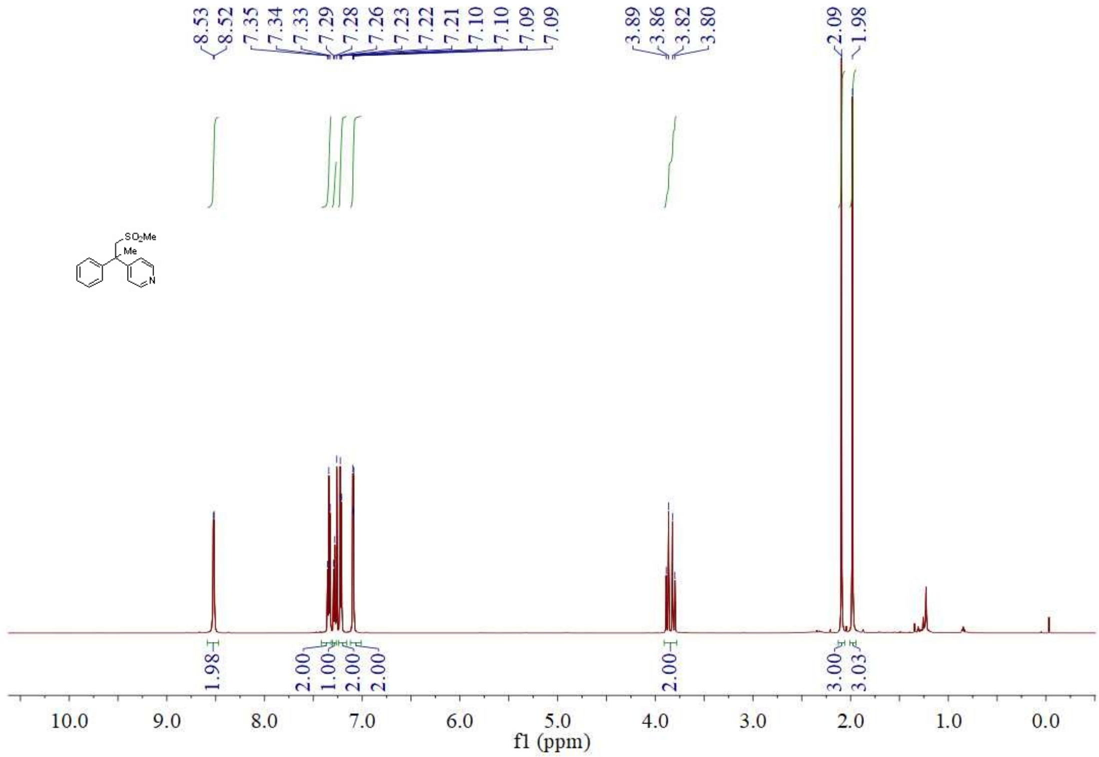


**19;** $^{13}$C NMR (101 MHz, $CDCl_3$)

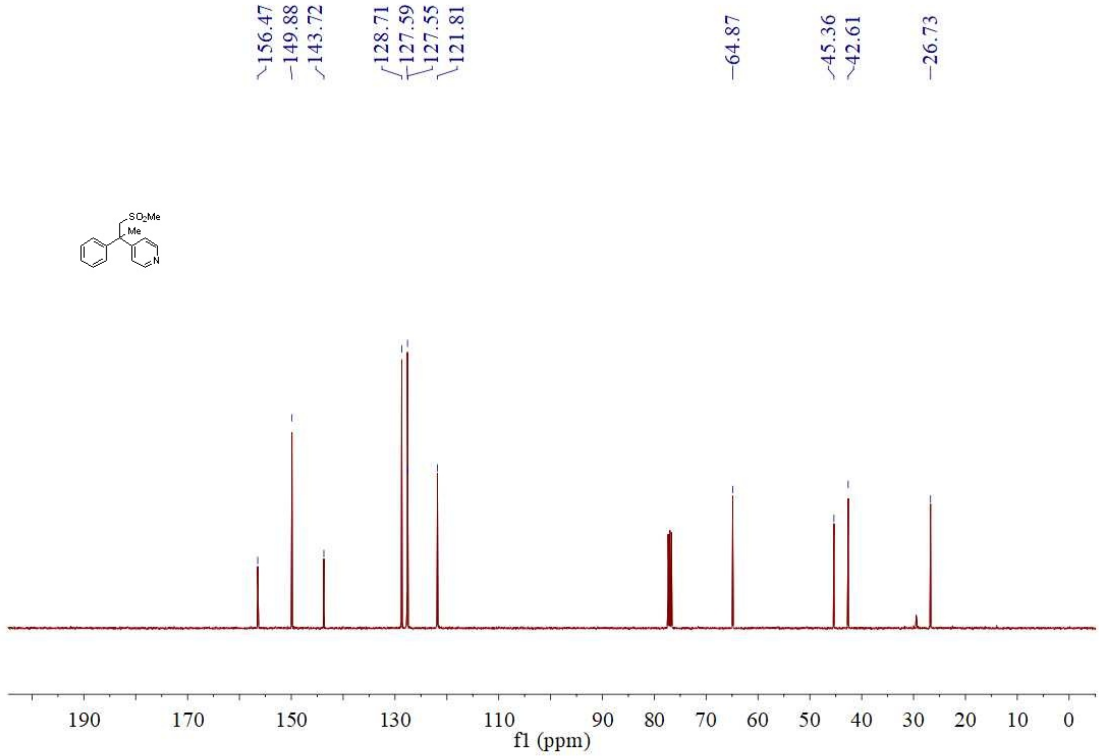

**20;** $^1$H NMR (600 MHz, $CDCl_3$)

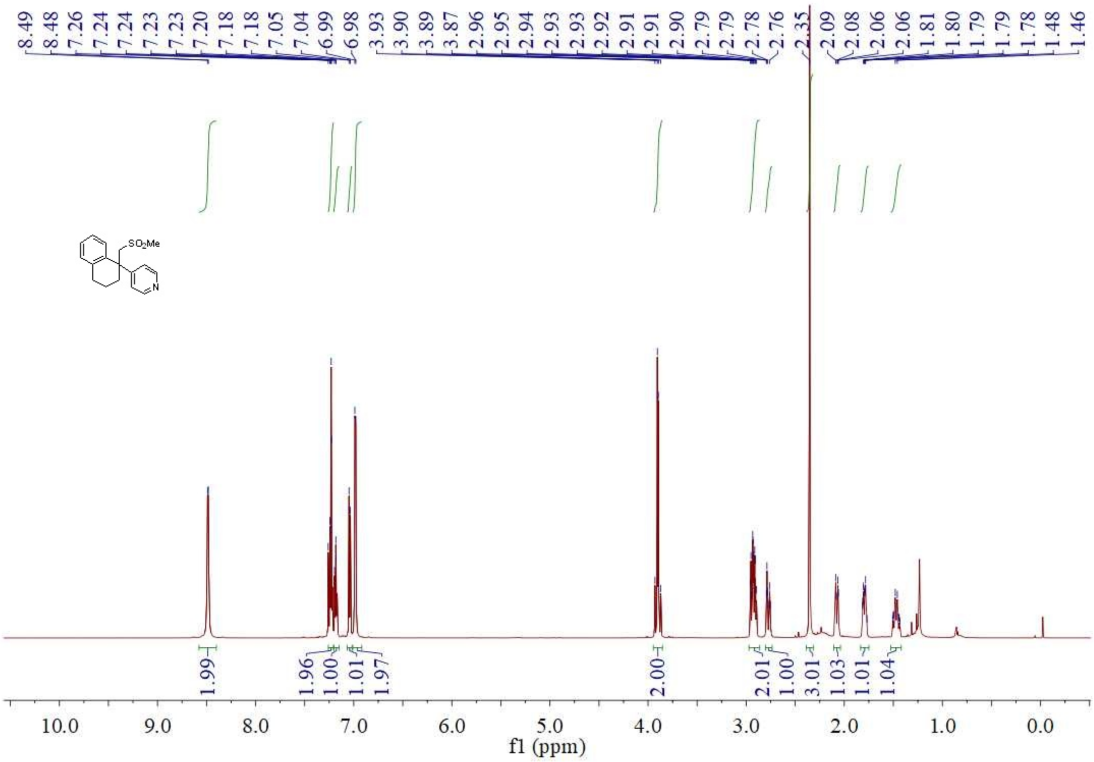


**20;** $^{13}$C NMR (101 MHz, $CDCl_3$)

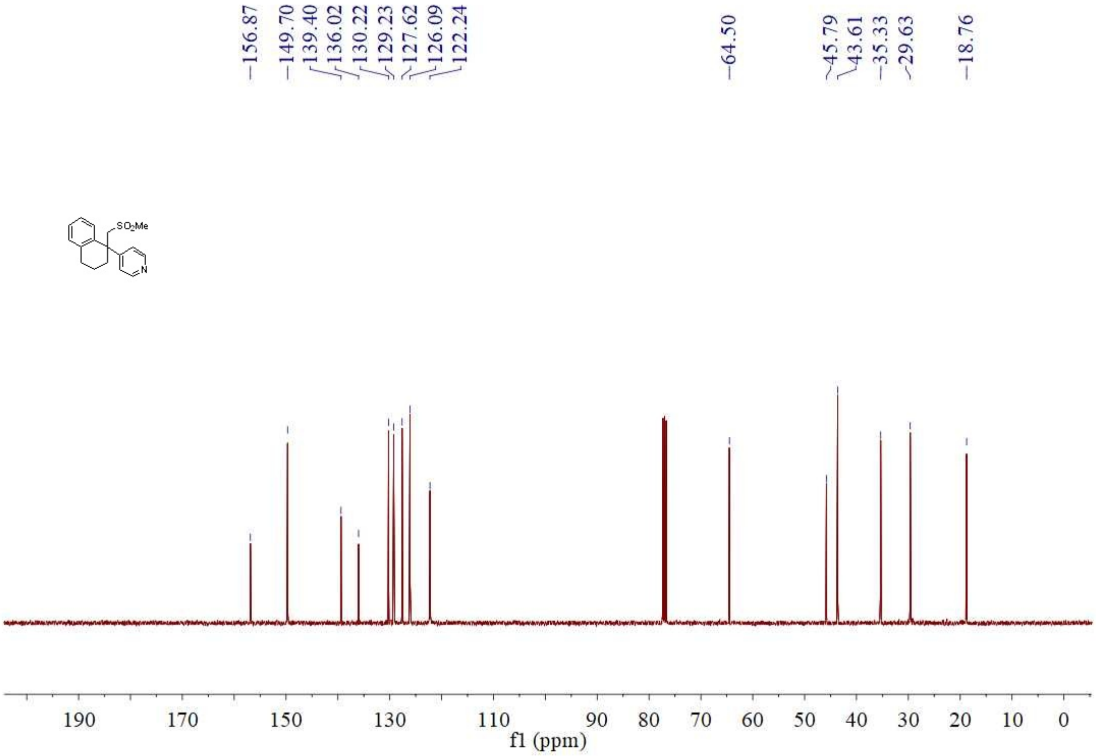

**21;** $^{1}$H NMR (600 MHz, $CDCl_3$)

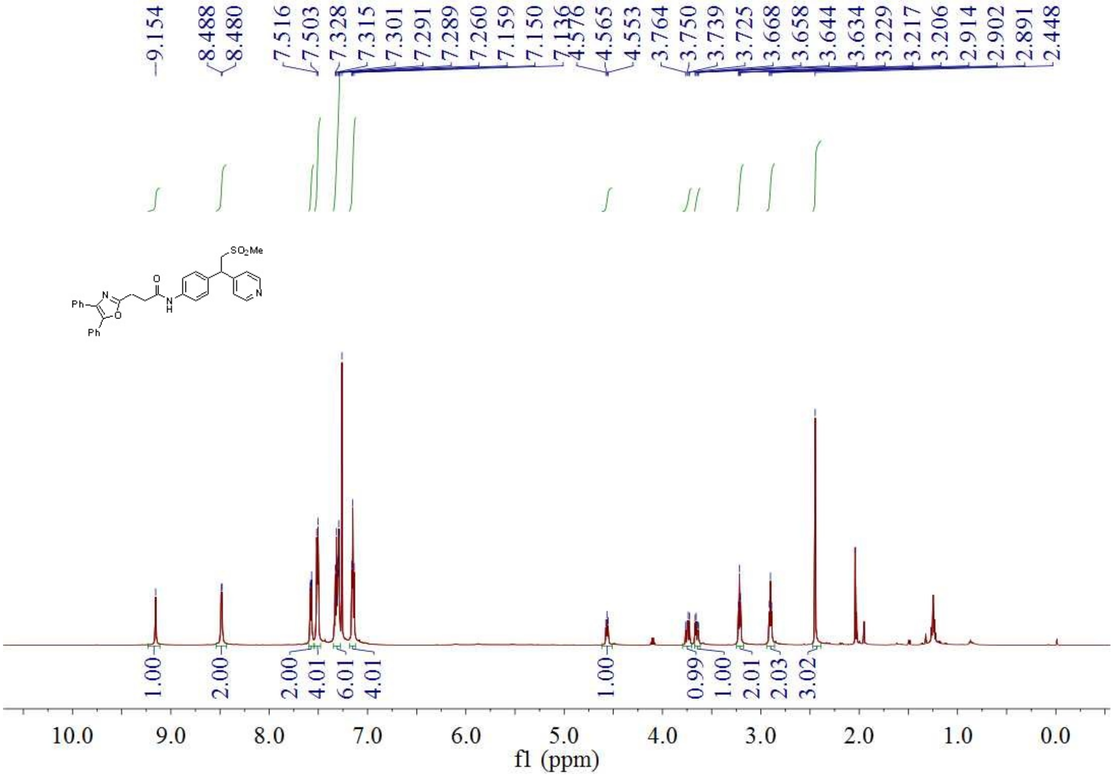


**21;** $^{13}$C NMR (101 MHz, $CDCl_3$)

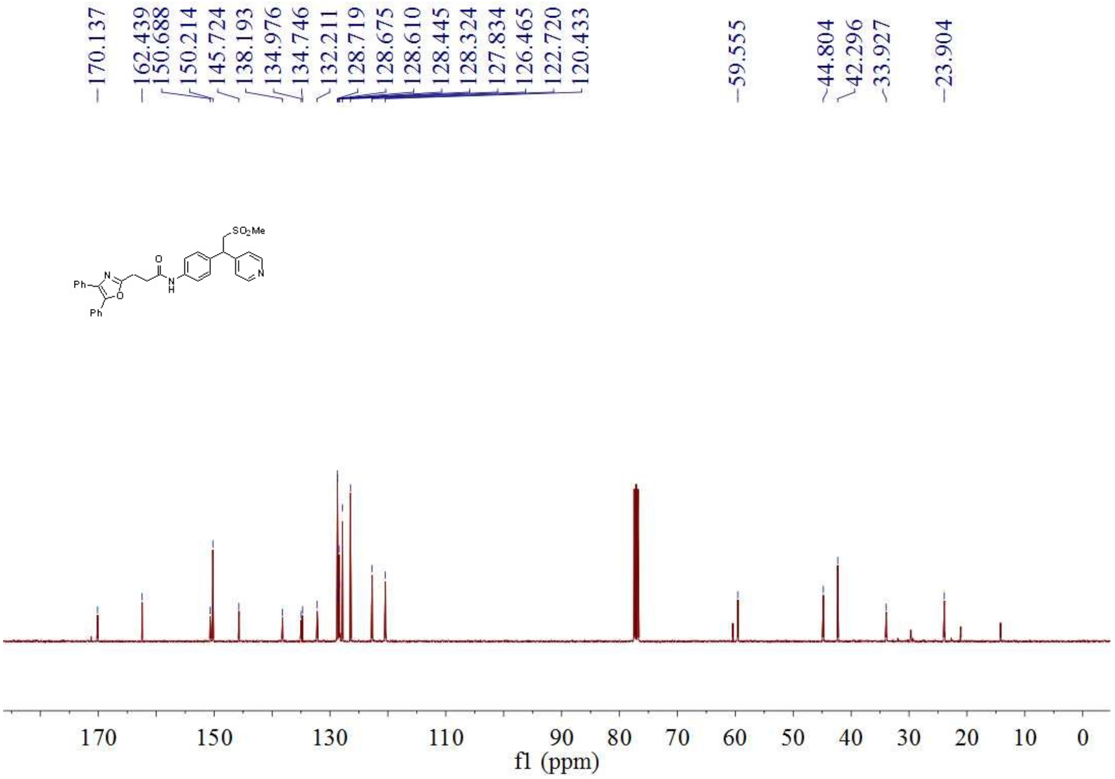

**22;** $^{1}$H NMR (400 MHz, $CDCl_3$)

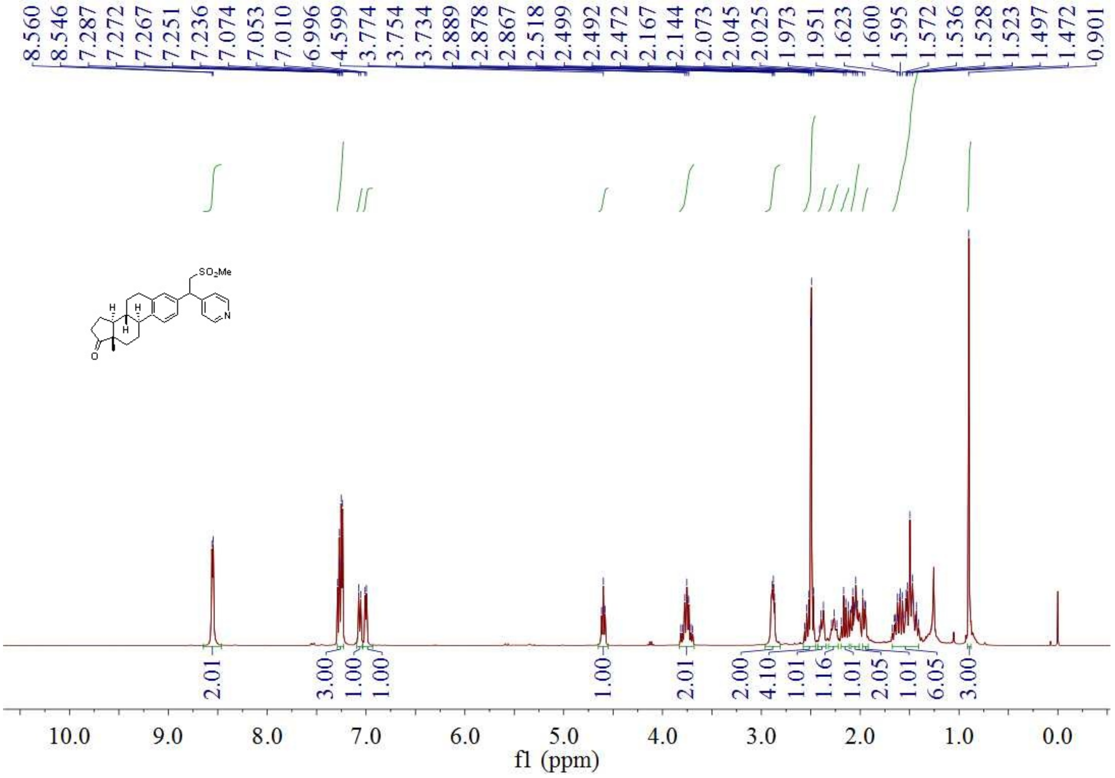


**22;** $^{13}$C NMR (101 MHz, $CDCl_3$)

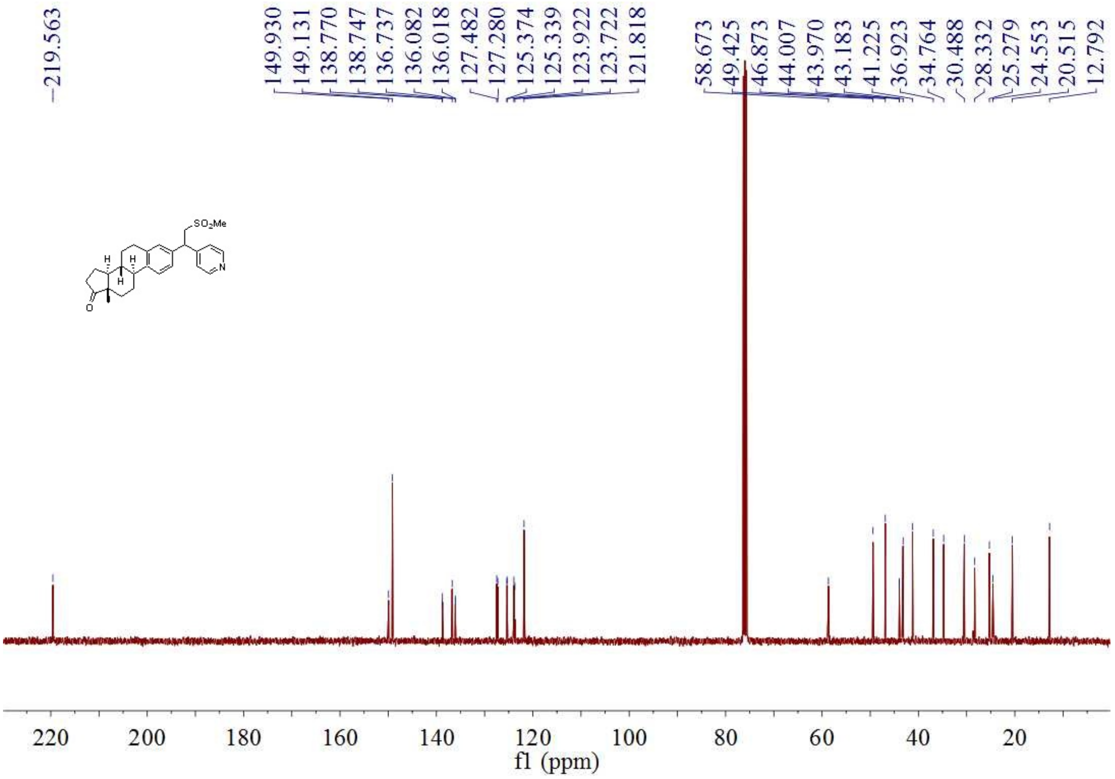

**23;** $^{1}$H NMR (400 MHz, DMSO-$d_6$)

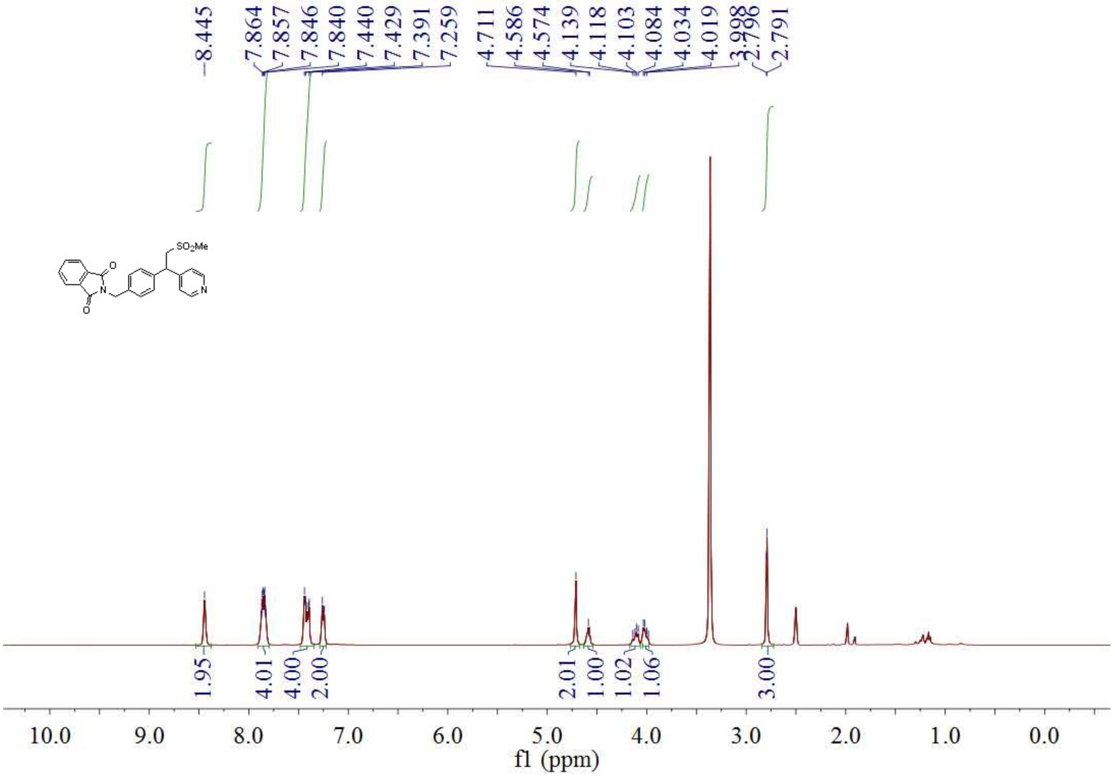


**23;** $^{13}$C NMR (101 MHz, DMSO-$d_6$)

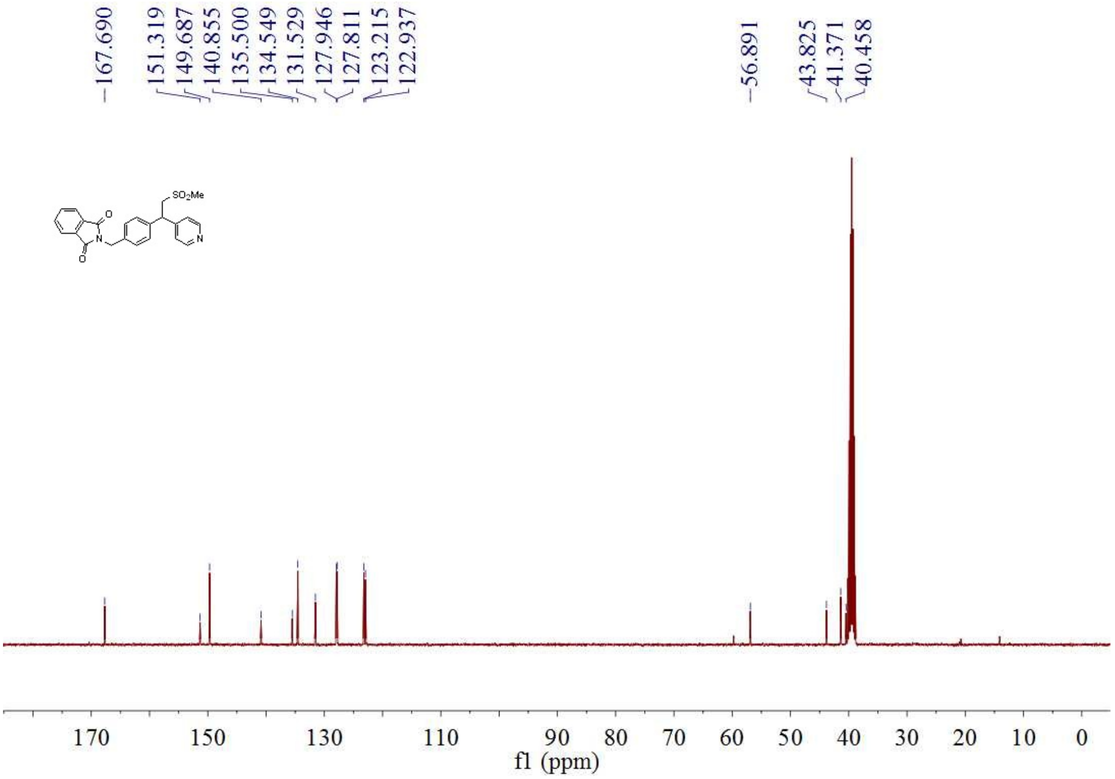

**24;** $^{1}$H NMR (400 MHz, $CDCl_3$)

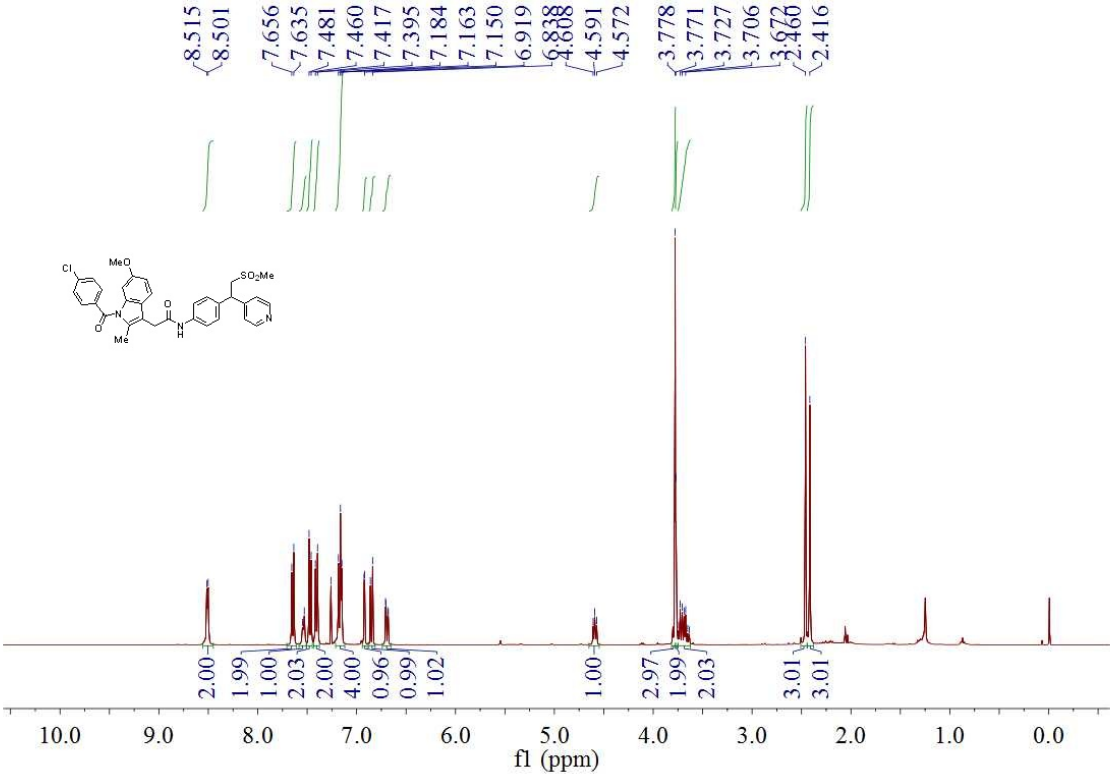


**24;** $^{13}$C NMR (101 MHz, $CDCl_3$)

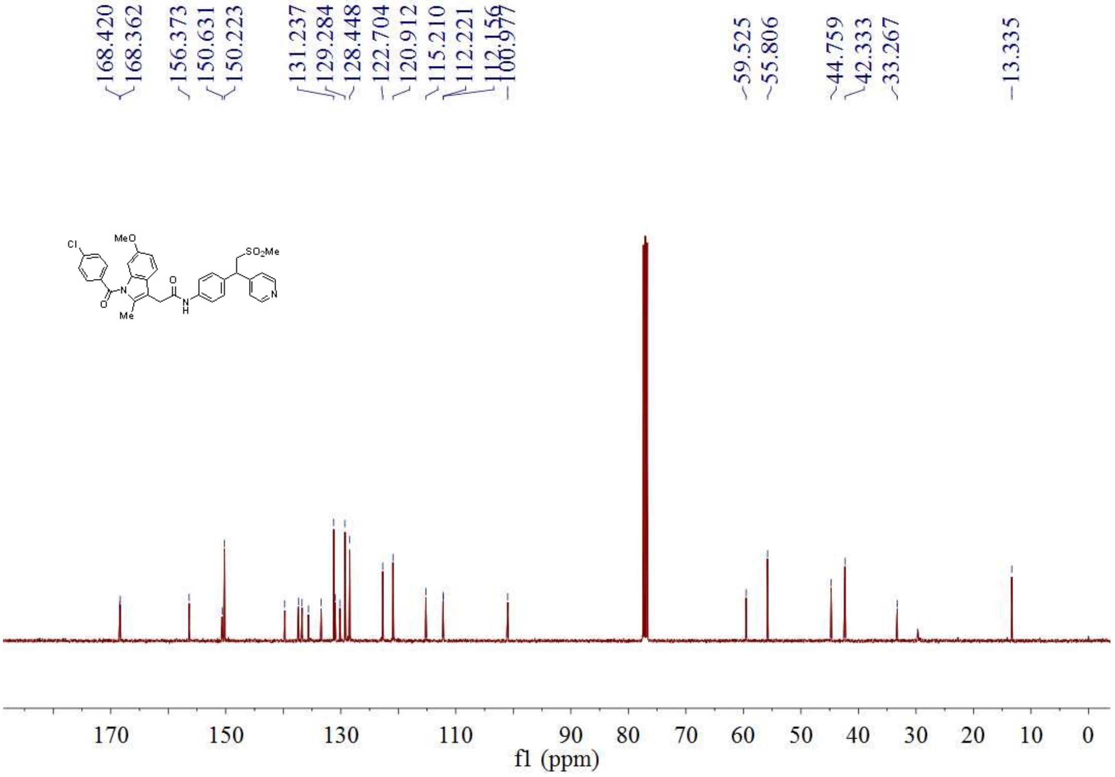

**25;** $^{1}$H NMR (600 MHz, $CDCl_3$)

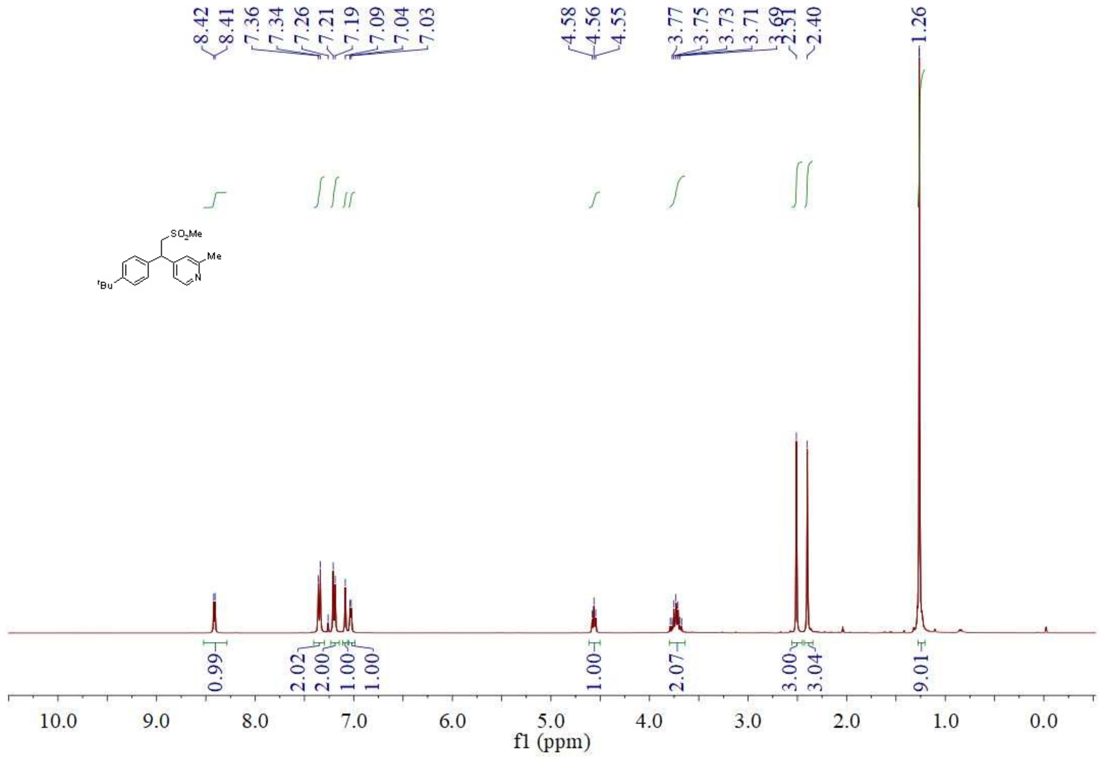


**25;** $^{13}$C NMR (101 MHz, $CDCl_3$)

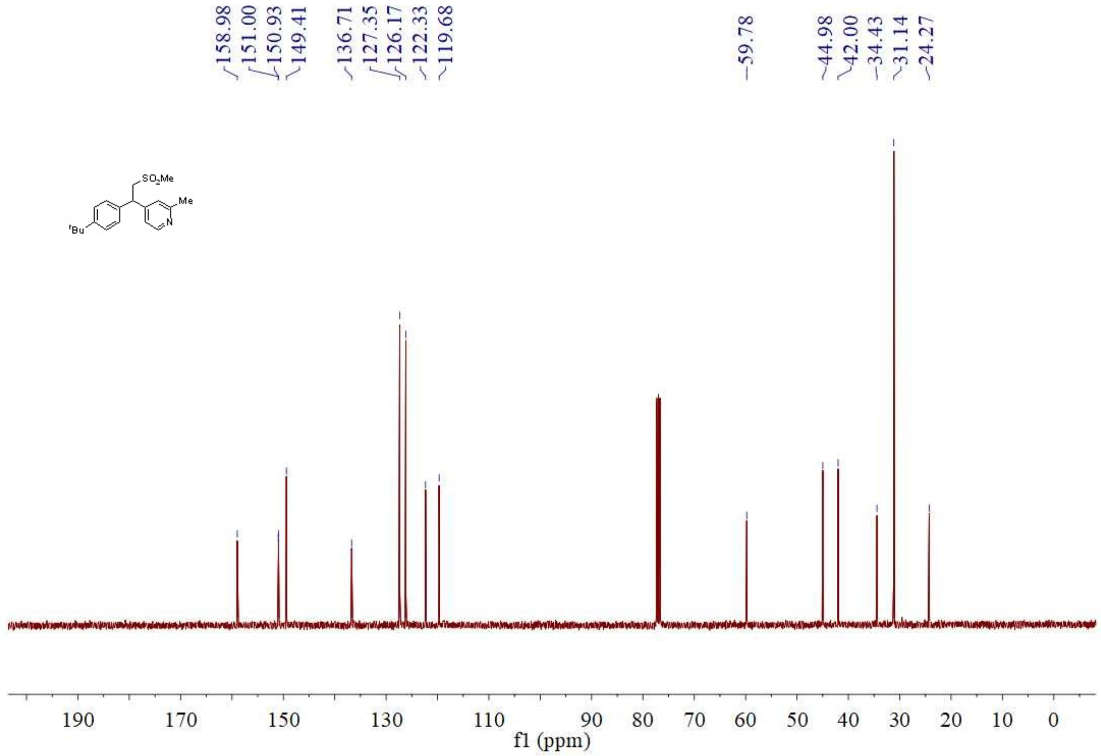

**26;** $^{1}$H NMR (400 MHz, CDCl$_3$)

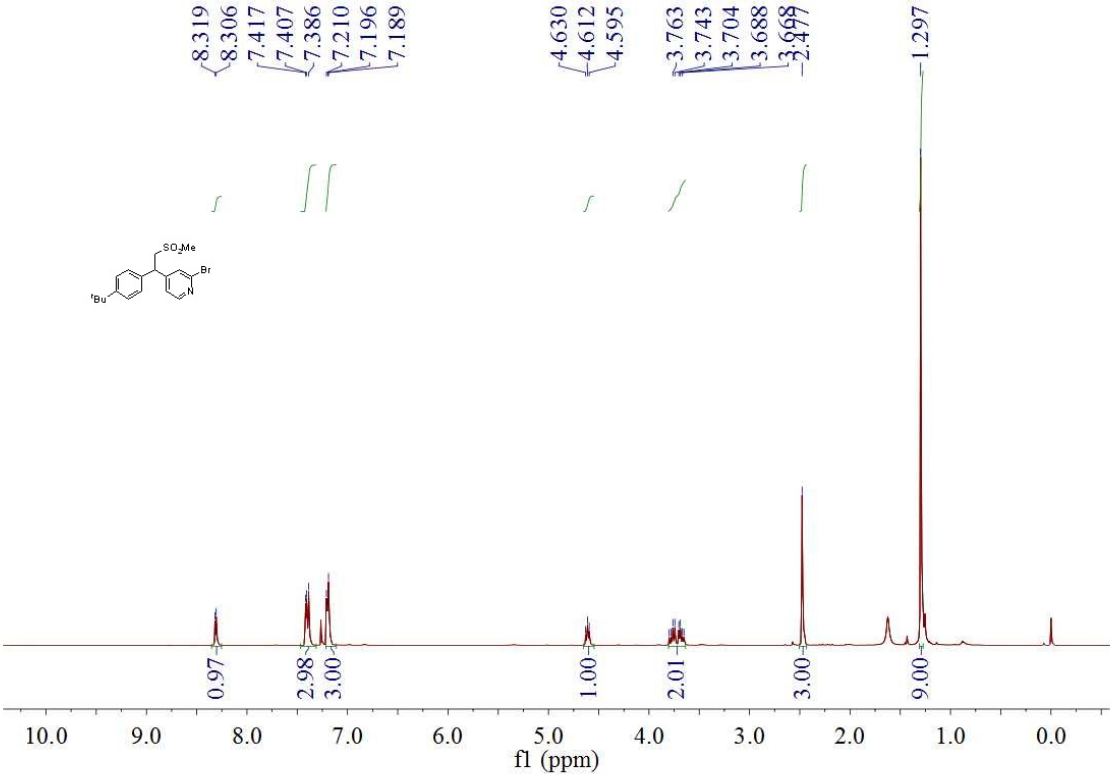


**26;** $^{13}$C NMR (101 MHz, CDCl$_3$)

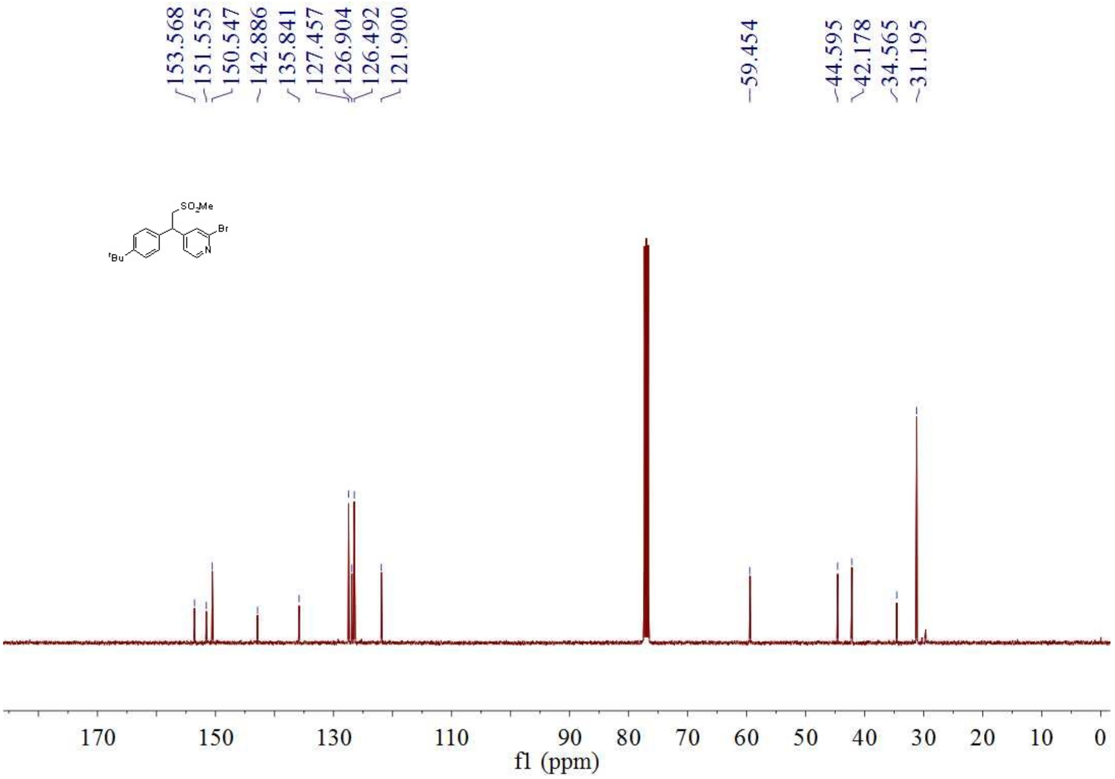

**27;** $^{1}$H NMR (400 MHz, $CDCl_3$)

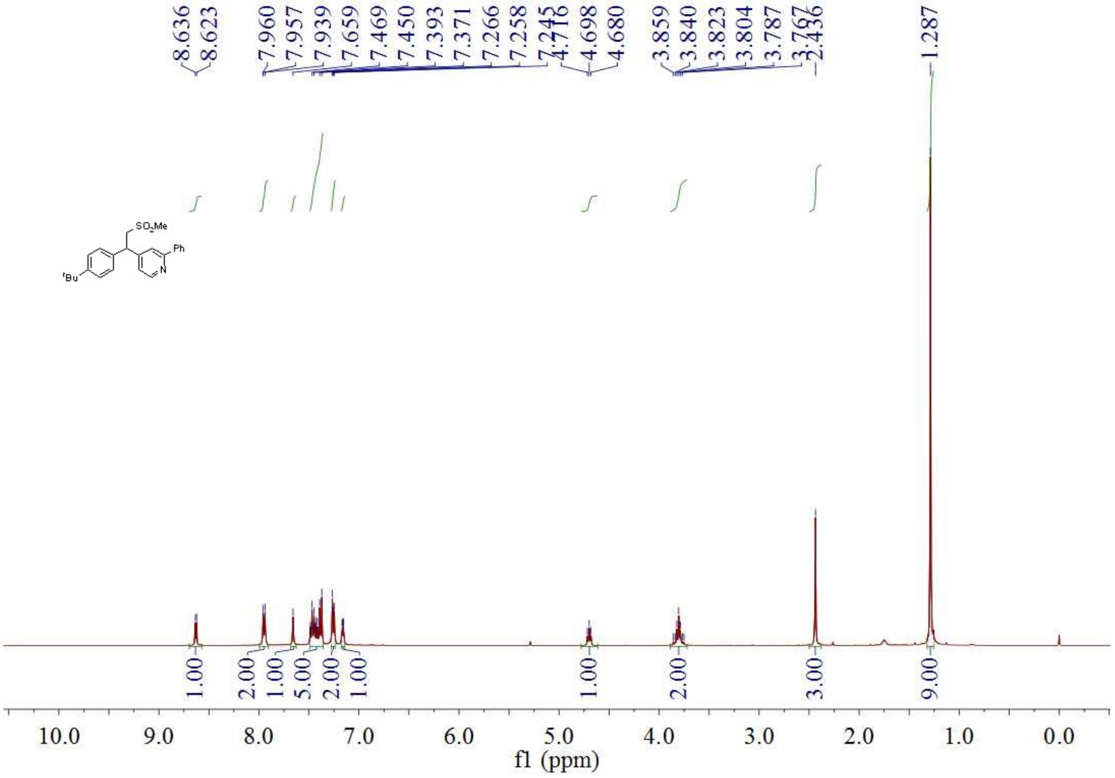


**27;** $^{13}$C NMR (101 MHz, $CDCl_3$)

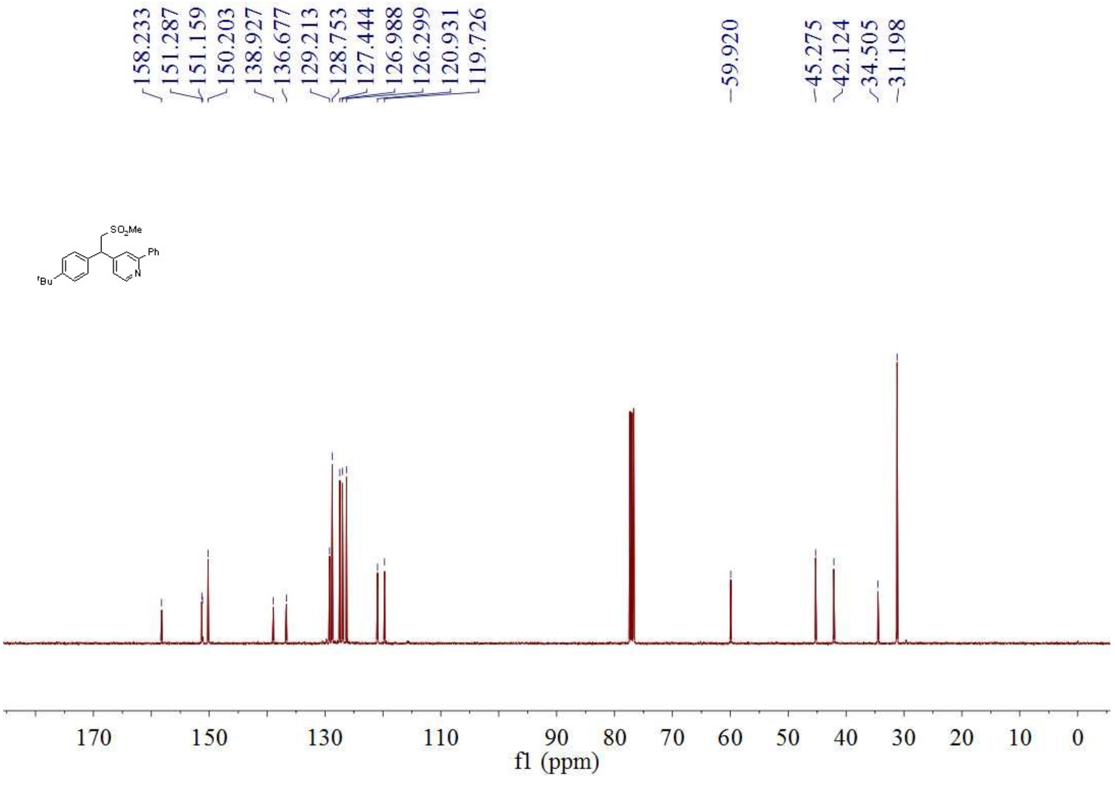

**28;** $^{1}$H NMR (600 MHz, $CDCl_3$)

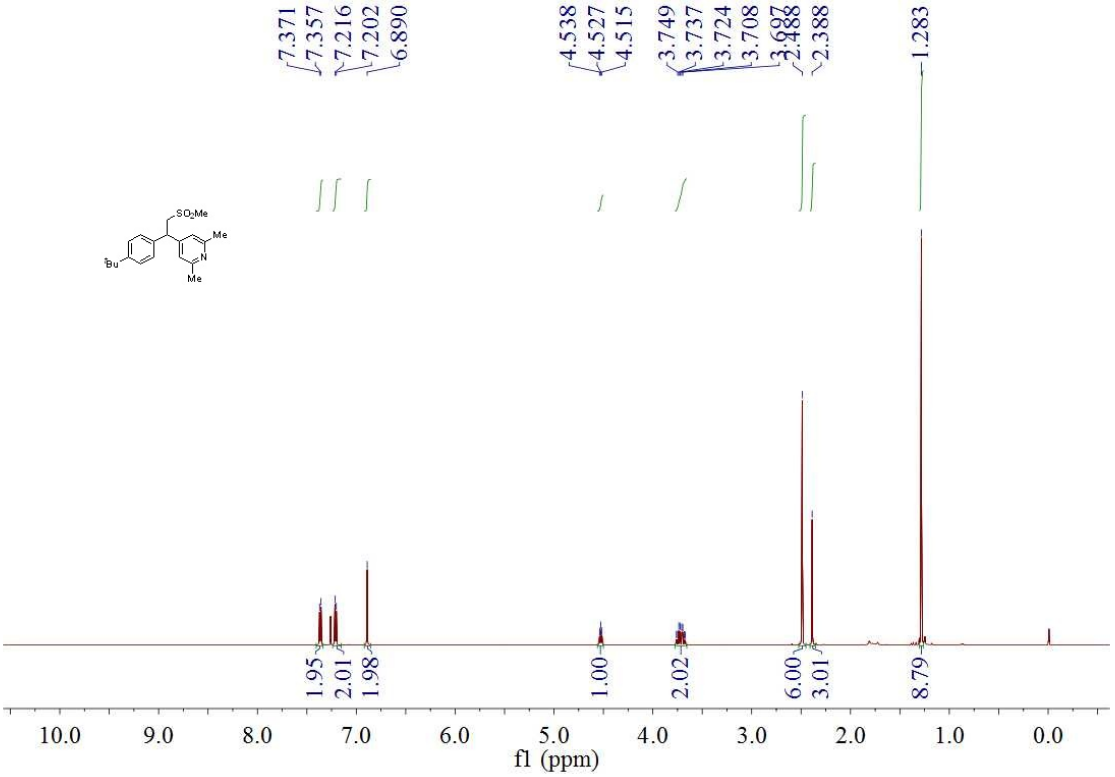


**28;** $^{13}$C NMR (101 MHz, $CDCl_3$)

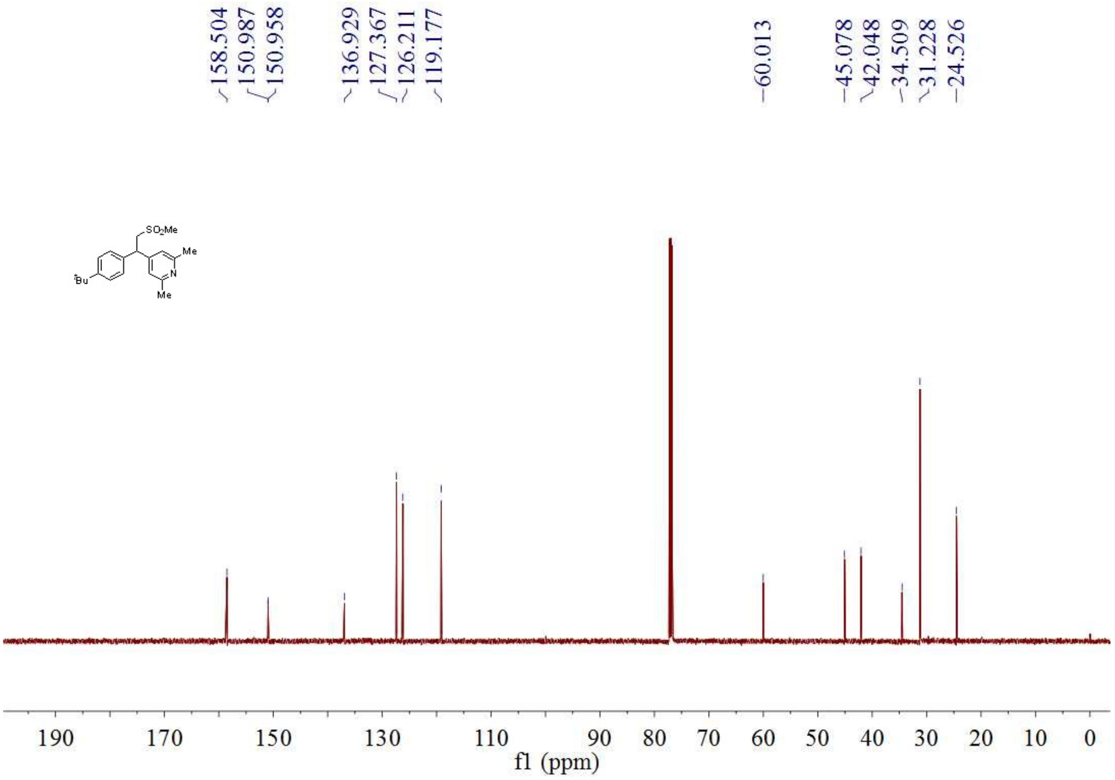

**29;** $^{1}$H NMR (600 MHz, $CDCl_3$)

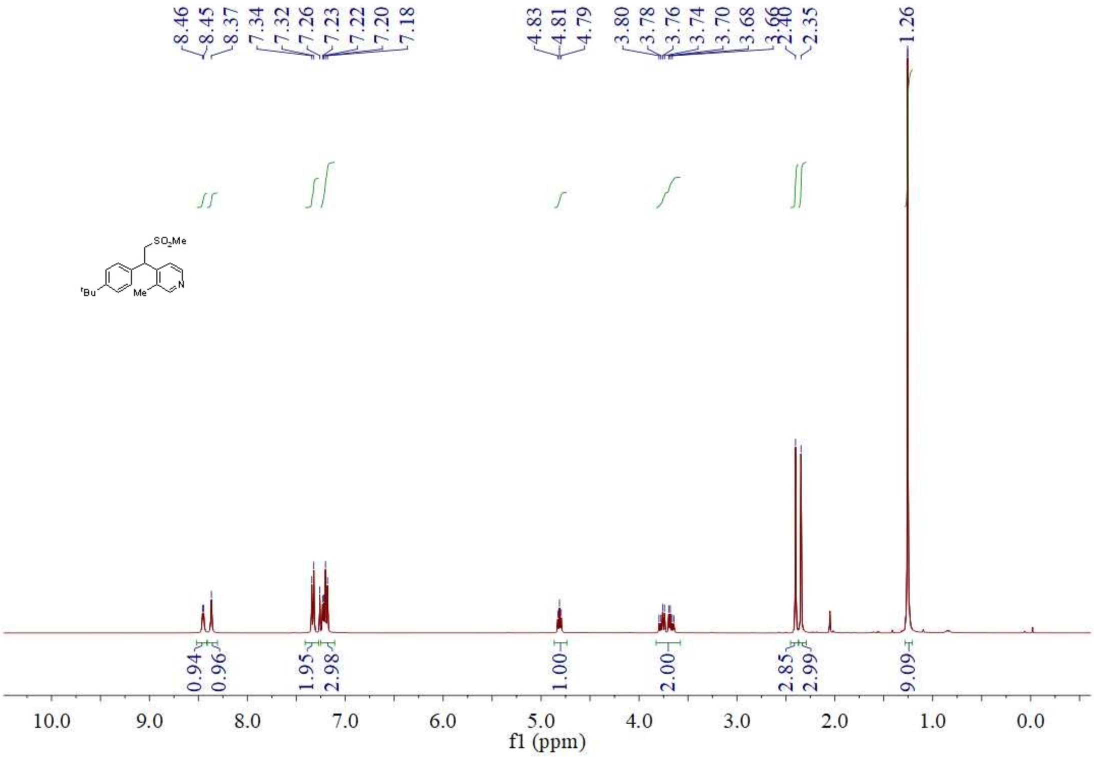


**29;** $^{13}$C NMR (101 MHz, $CDCl_3$)

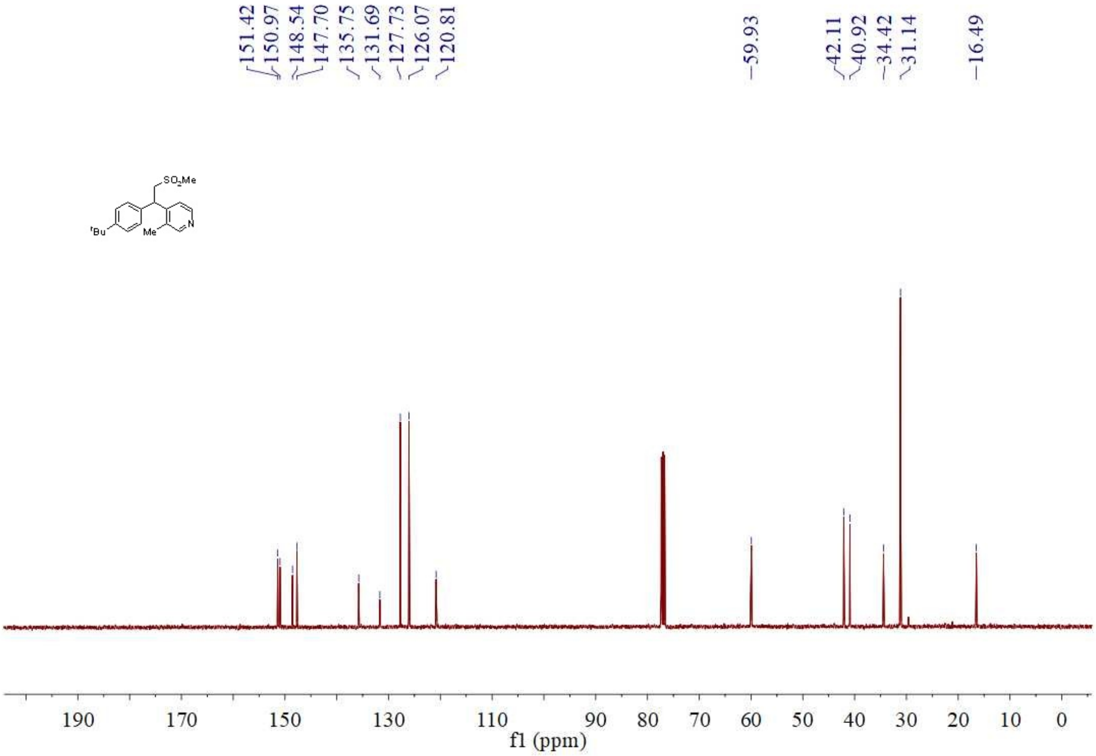

**30;** $^{1}$H NMR (600 MHz, $CDCl_3$)

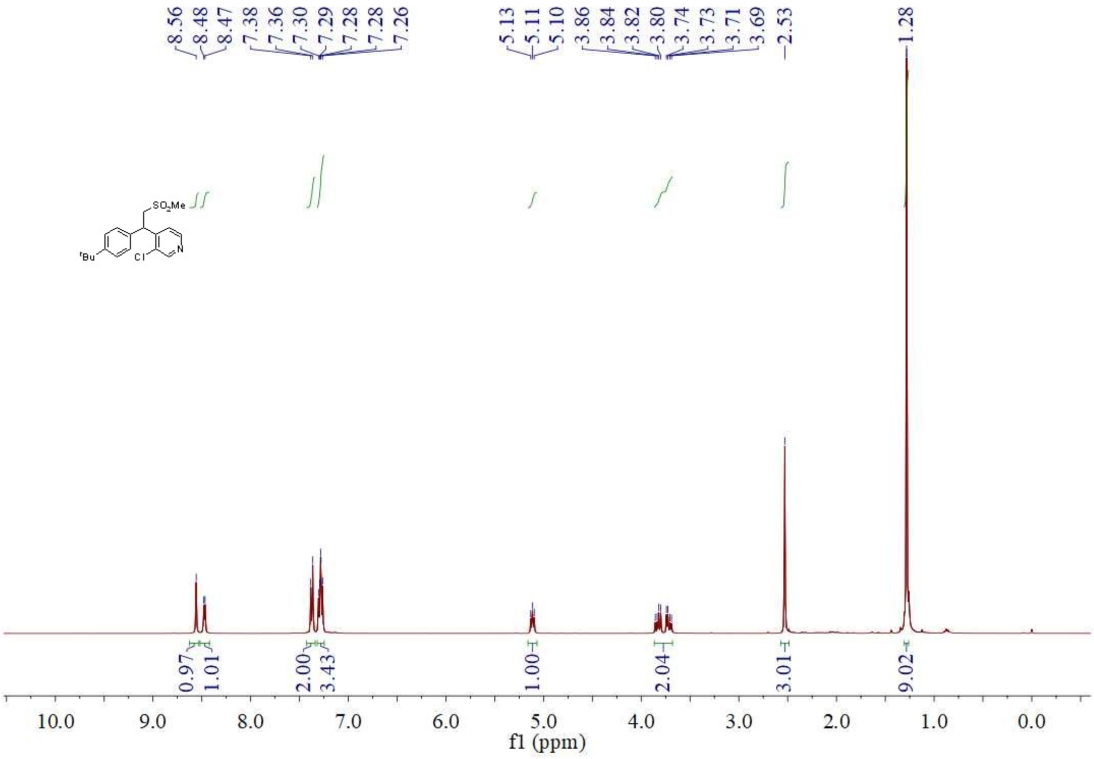

**30;** $^{13}$C NMR (101 MHz, $CDCl_3$)

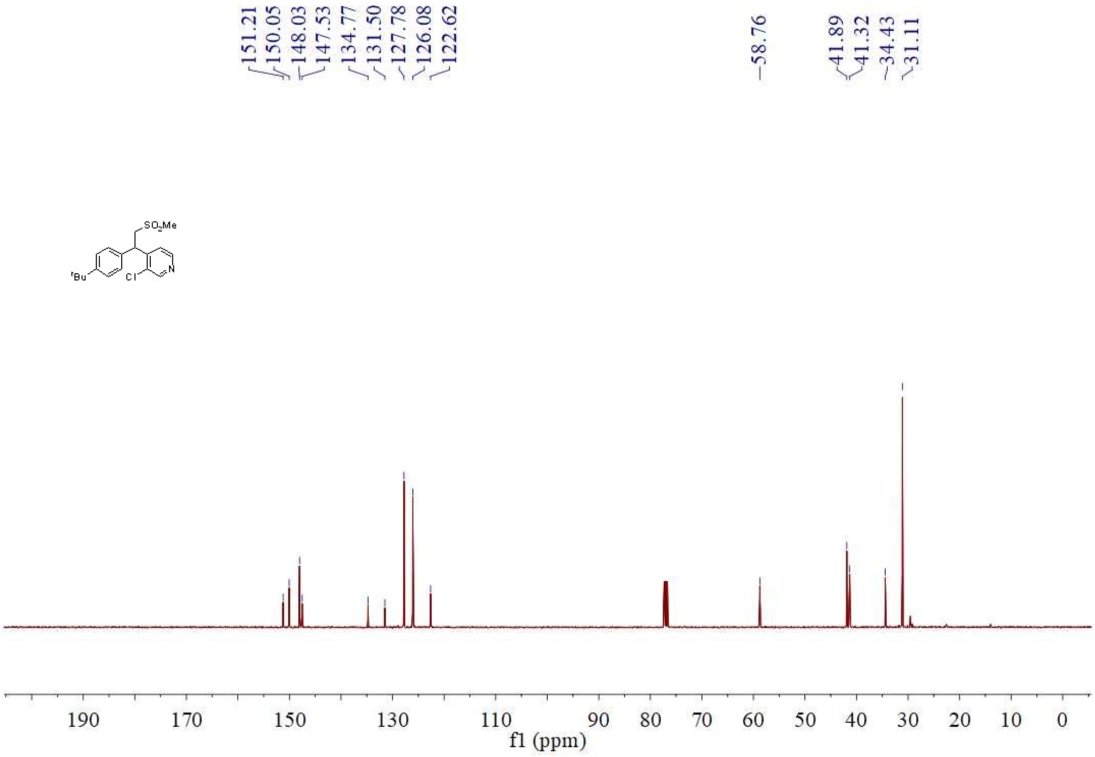

**31;** $^{1}$H NMR (600 MHz, $CDCl_3$)

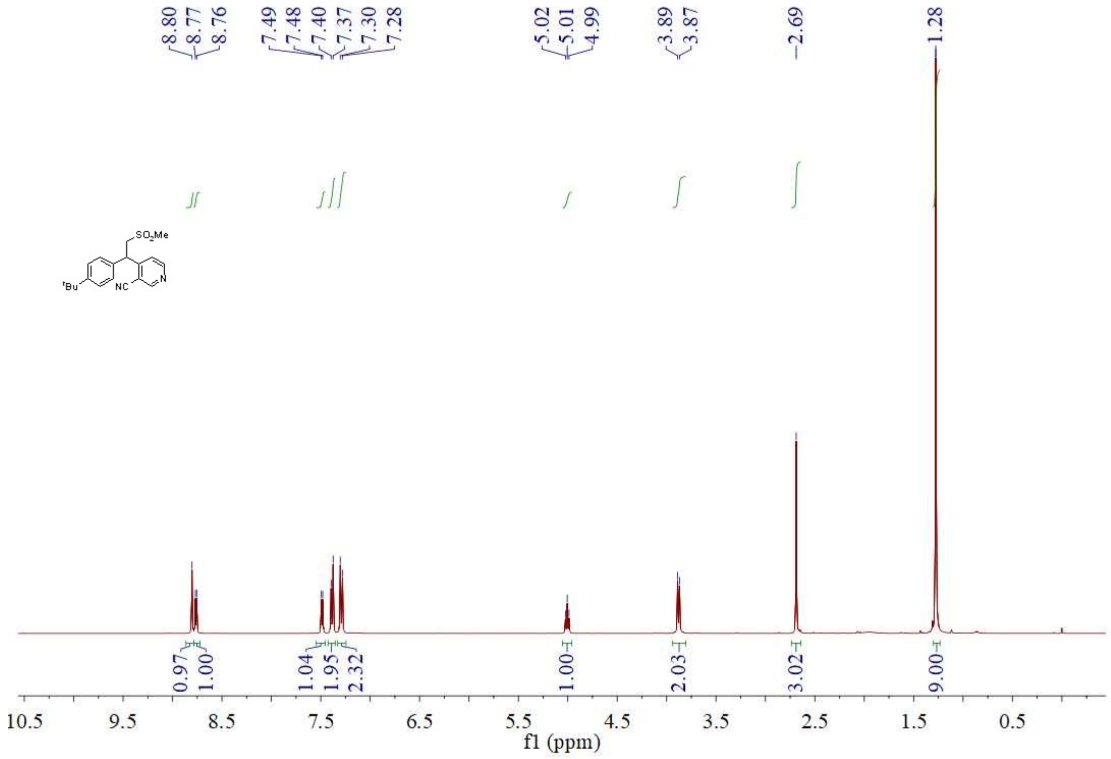


**31;** $^{13}$C NMR (101 MHz, $CDCl_3$)

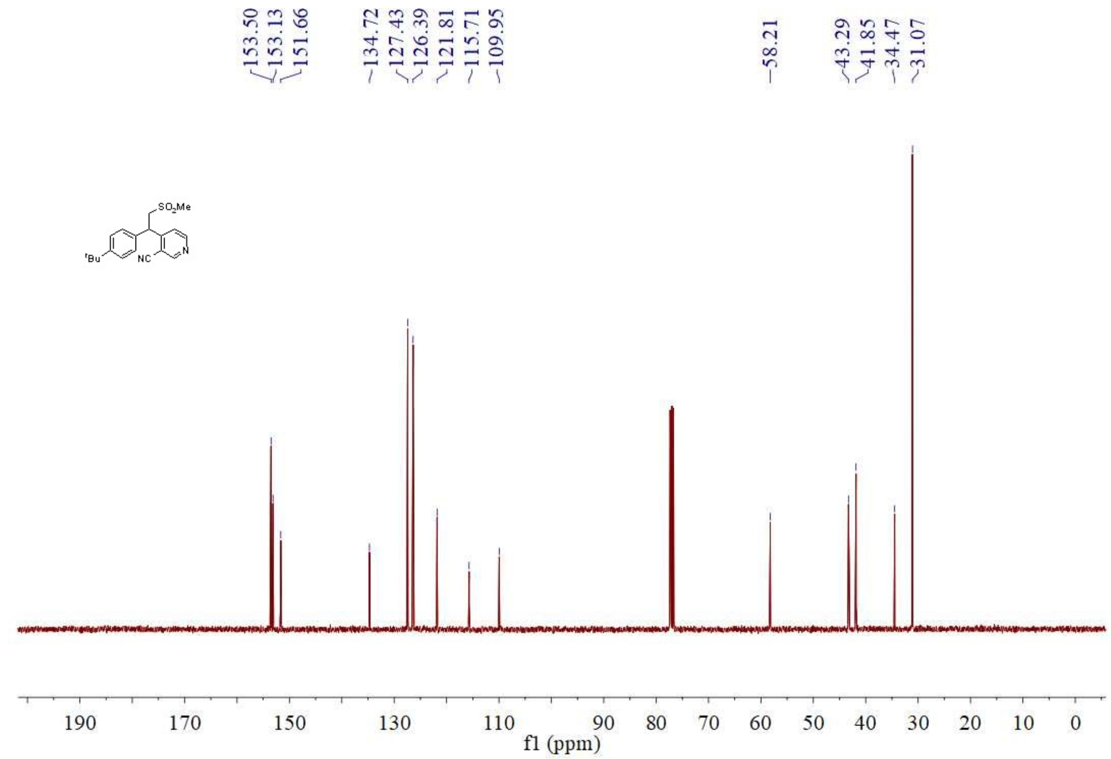

**32;** $^1$H NMR (400 MHz, $CDCl_3$)

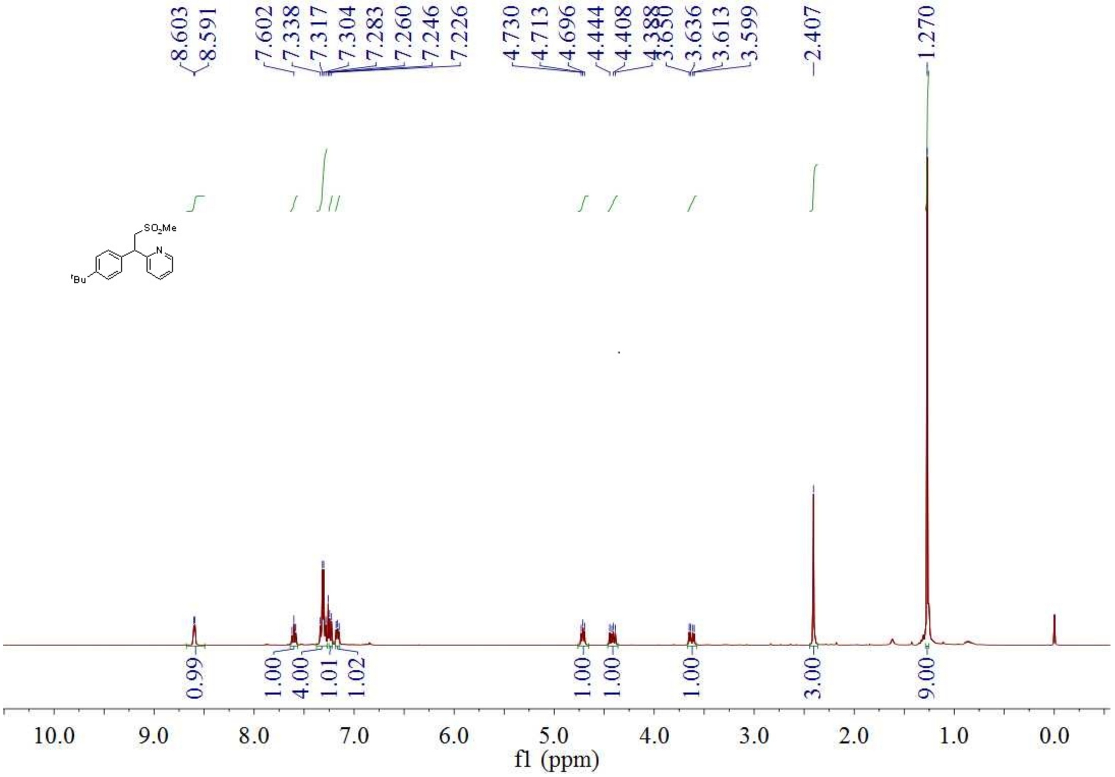

**32;** $^{13}$C NMR (101 MHz, $CDCl_3$)

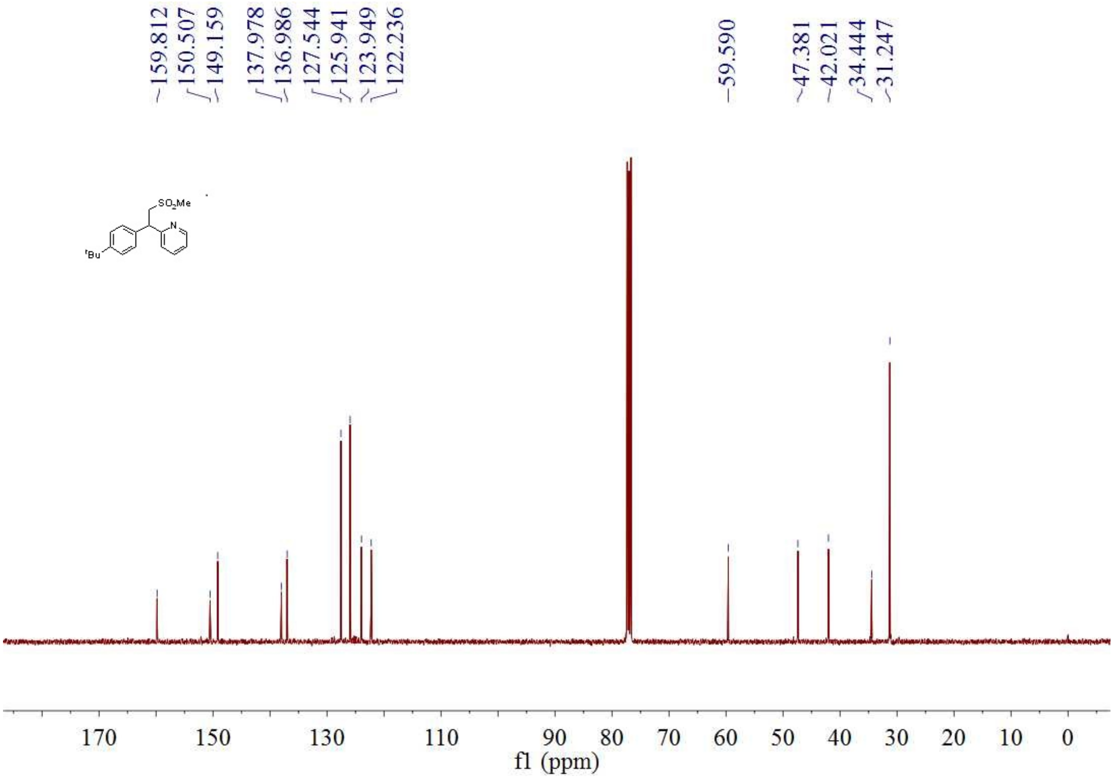

**33;** $^{1}$H NMR (400 MHz, $CDCl_3$)

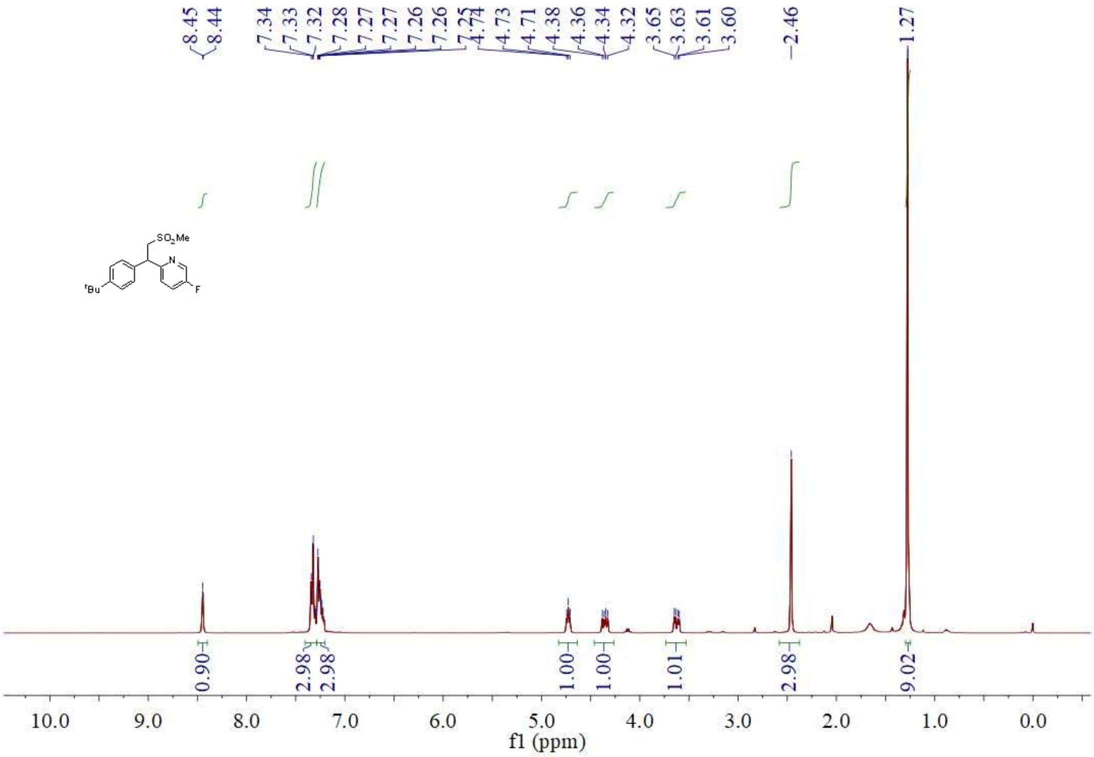


**33;** $^{13}$C NMR (101 MHz, $CDCl_3$)

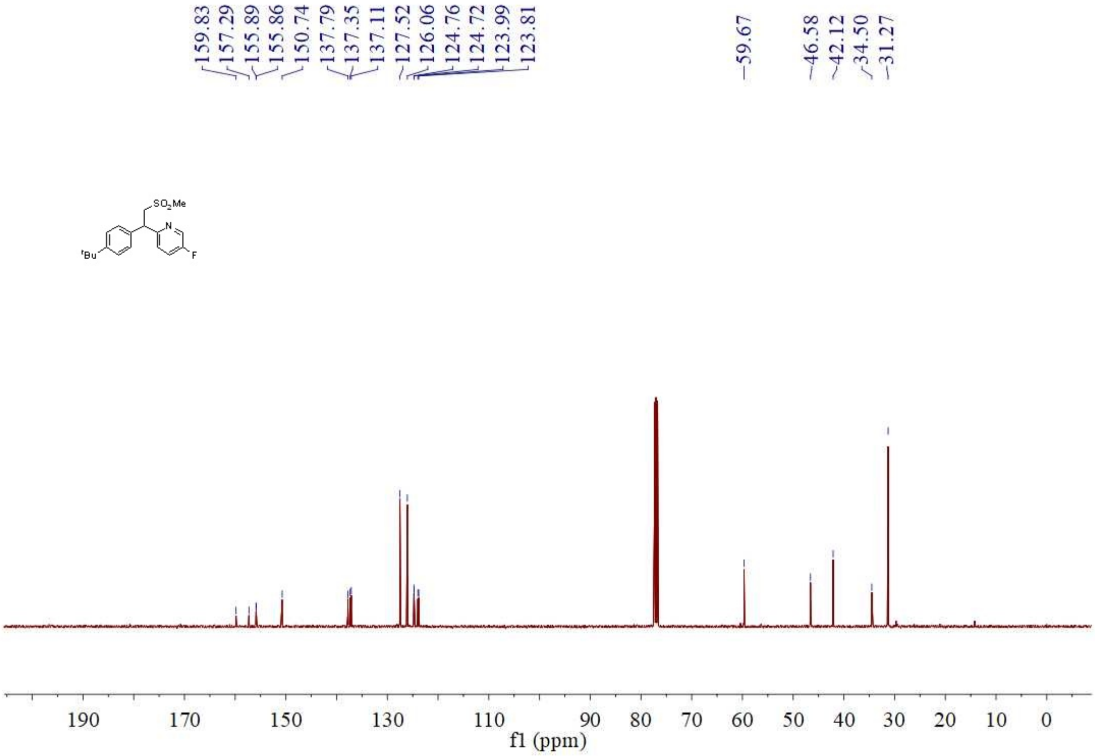

**33;** $^{19}$F NMR (377 MHz, $CDCl_3$)

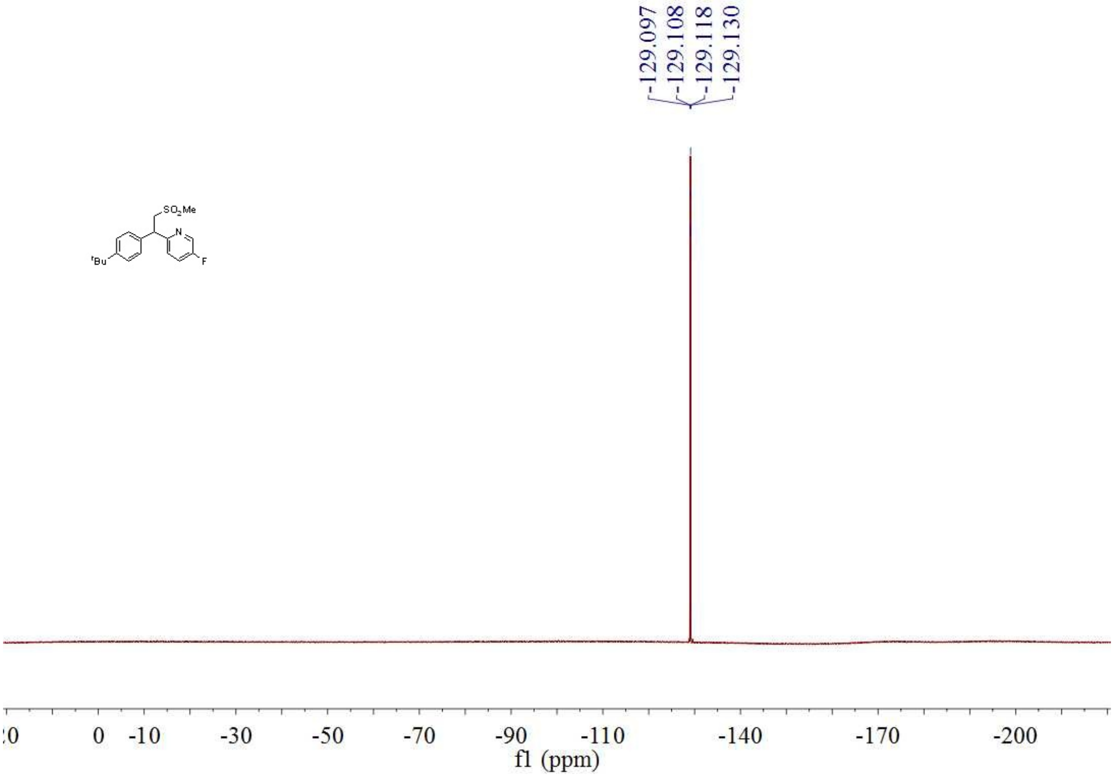


**34;** $^{1}$H NMR (400 MHz, $CDCl_3$)

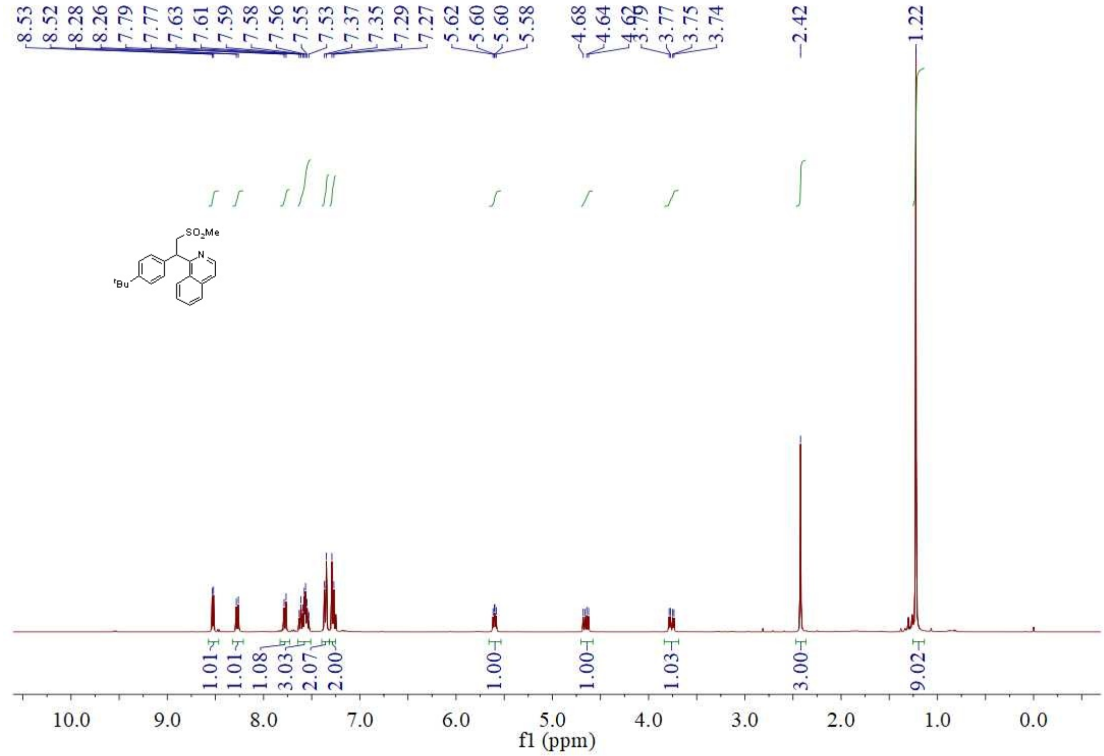

**34;** $^{13}$C NMR (101 MHz, $CDCl_3$)

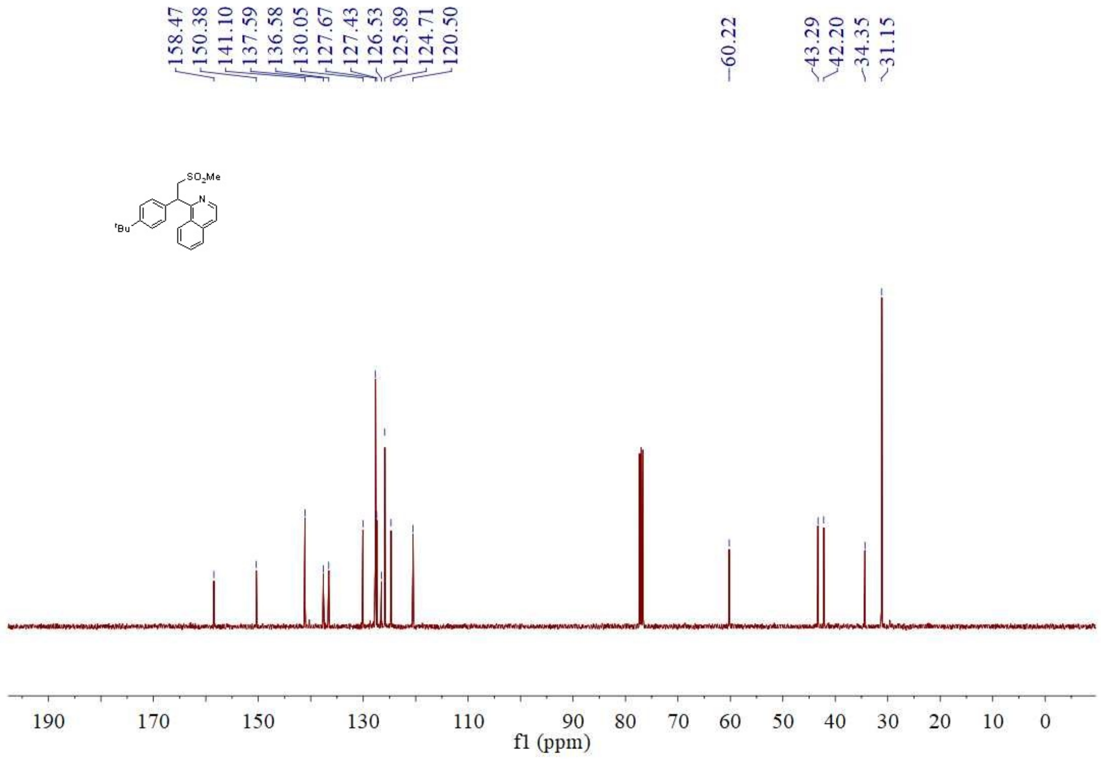


**35;** $^{1}$H NMR (400 MHz, $CDCl_3$)

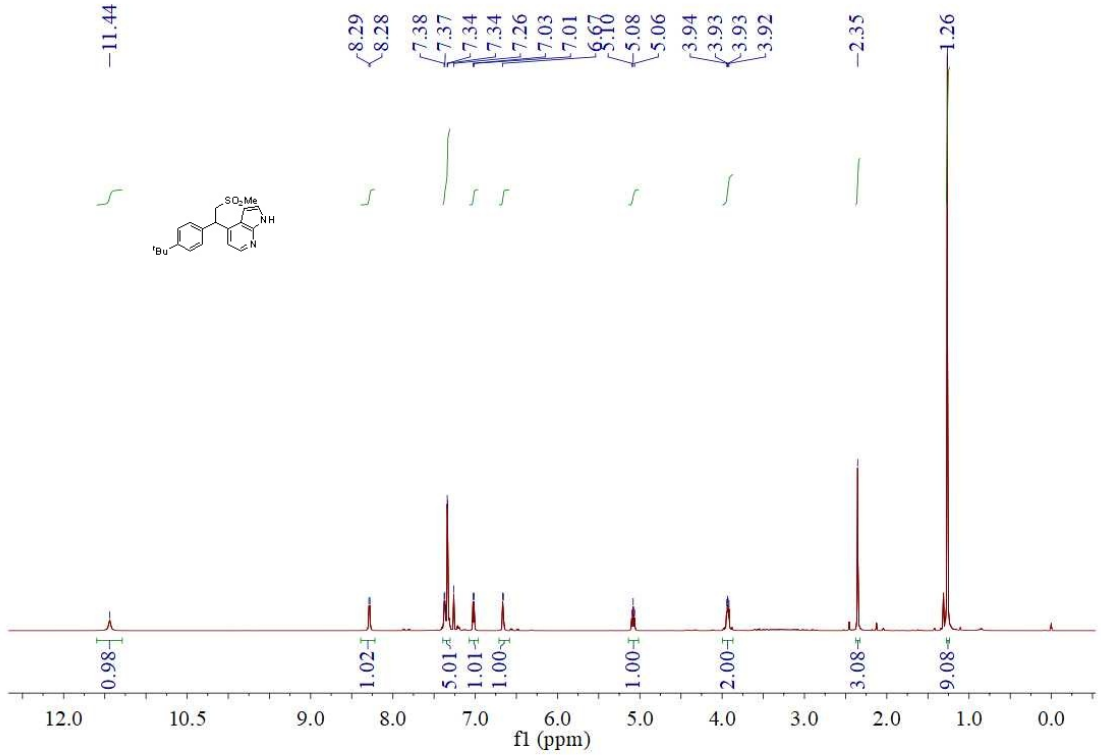

**35;** $^{13}$C NMR (101 MHz, $CDCl_3$)

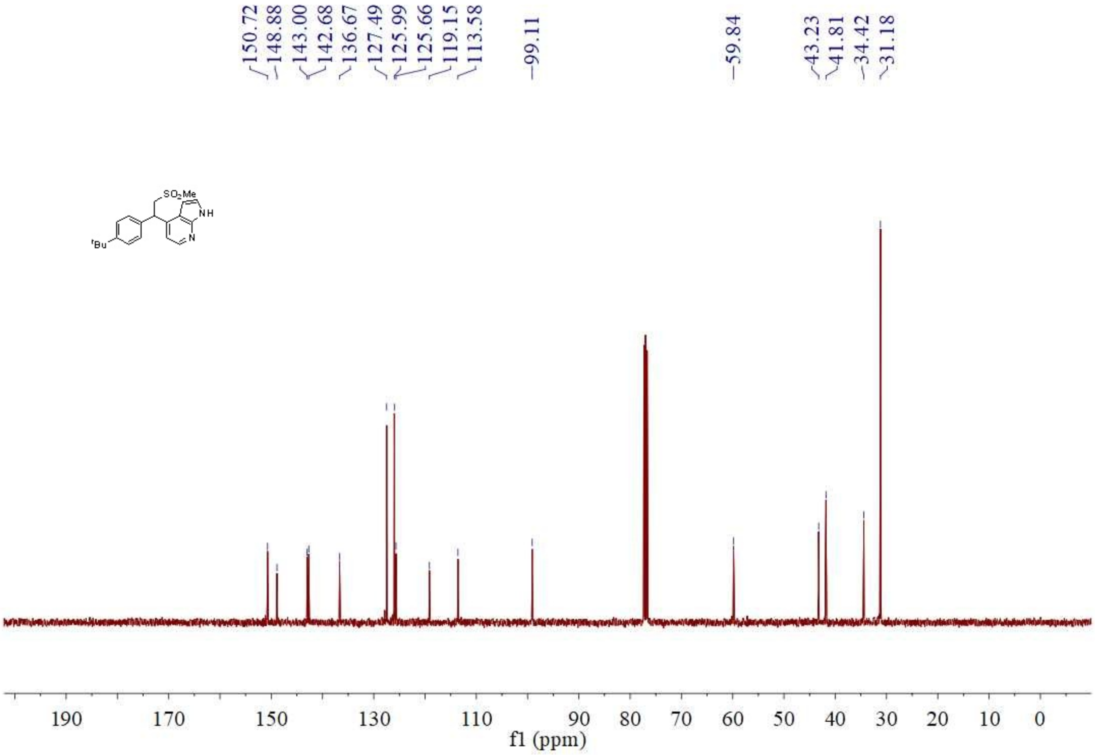


**36;** $^{1}$H NMR (600 MHz, $CDCl_3$)

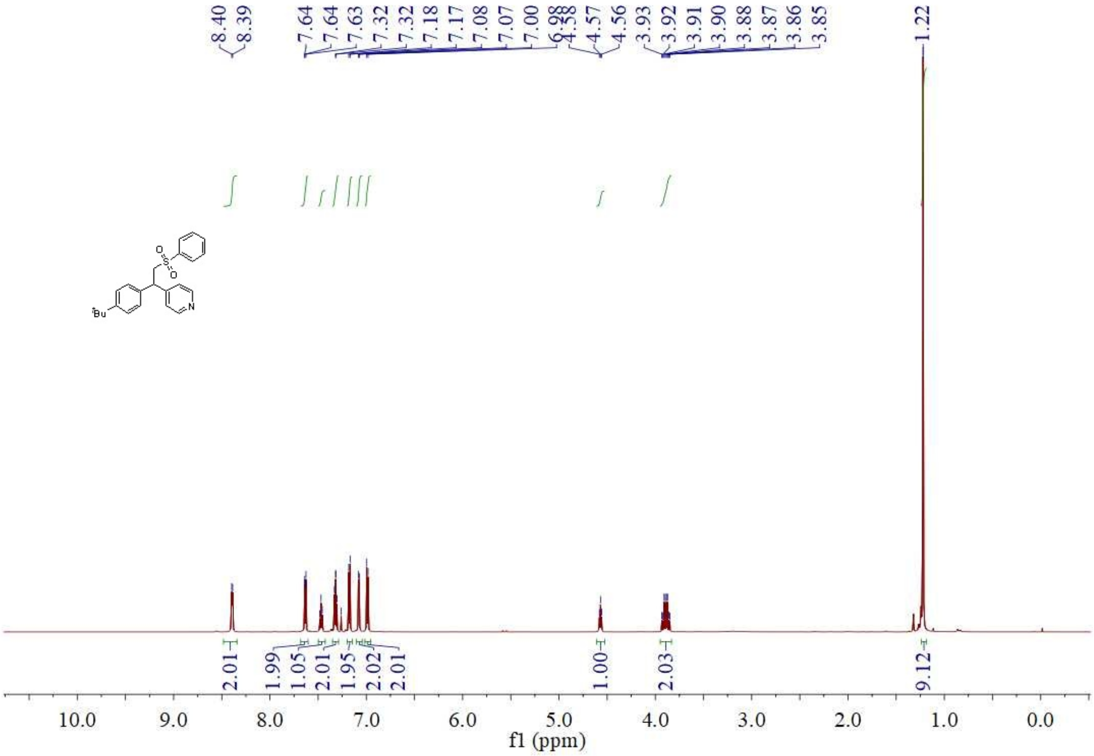

**36;** $^{13}$C NMR (101 MHz, $CDCl_3$)

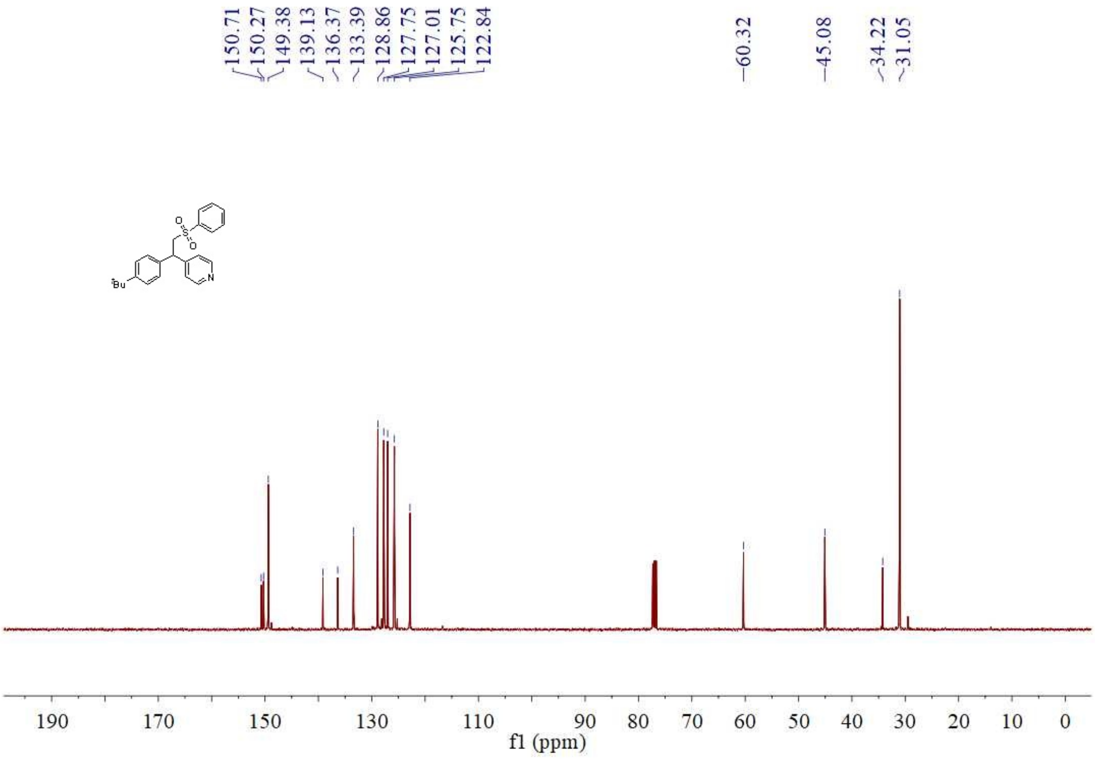


**37;** $^{1}$H NMR (600 MHz, $CDCl_3$)

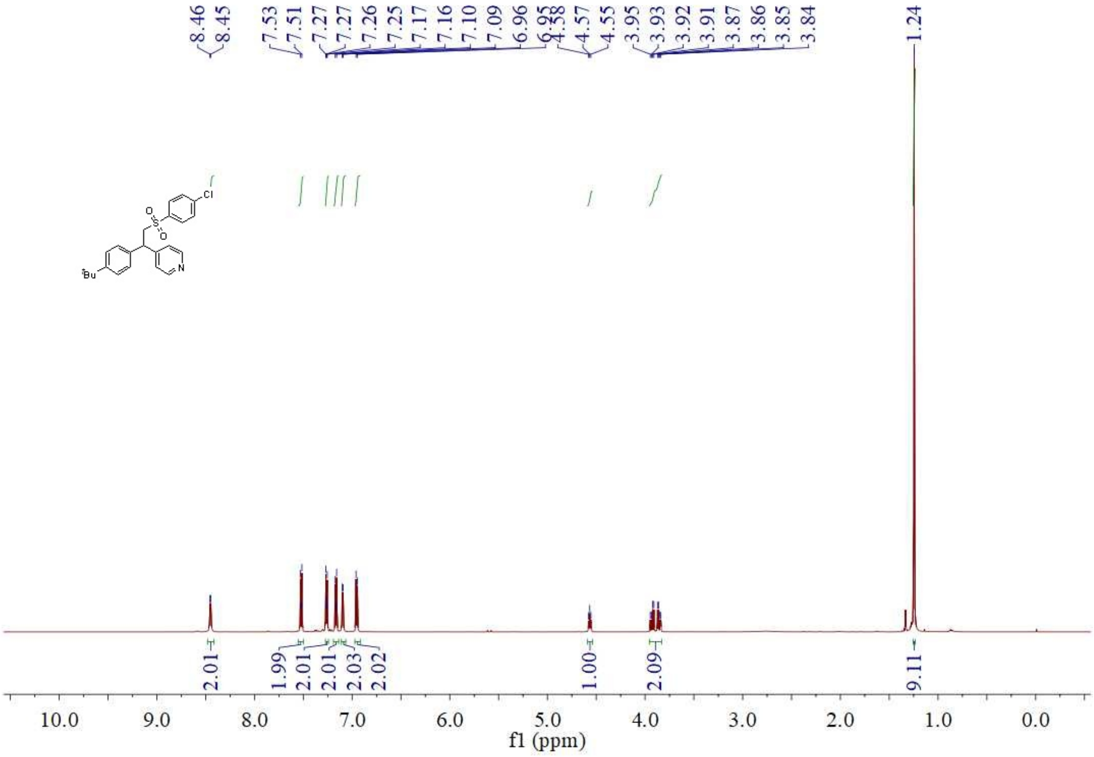

**37;** $^{13}$C NMR (101 MHz, $CDCl_3$)

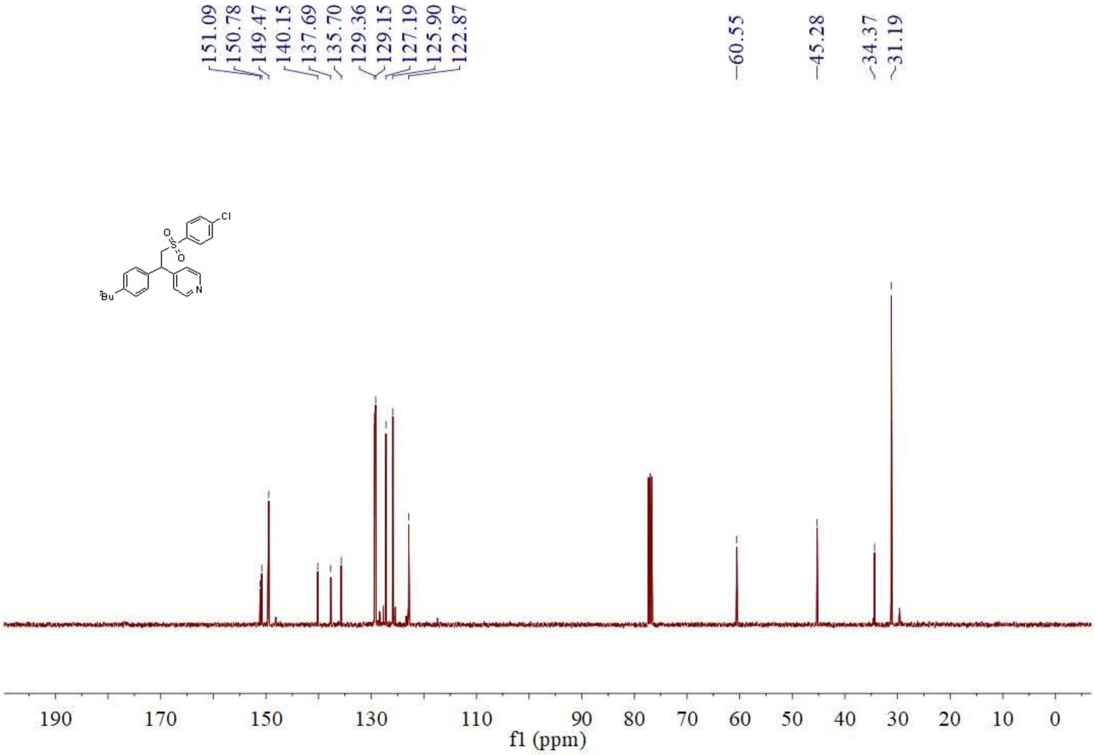


**38;** $^{1}$H NMR (600 MHz, $CDCl_3$)

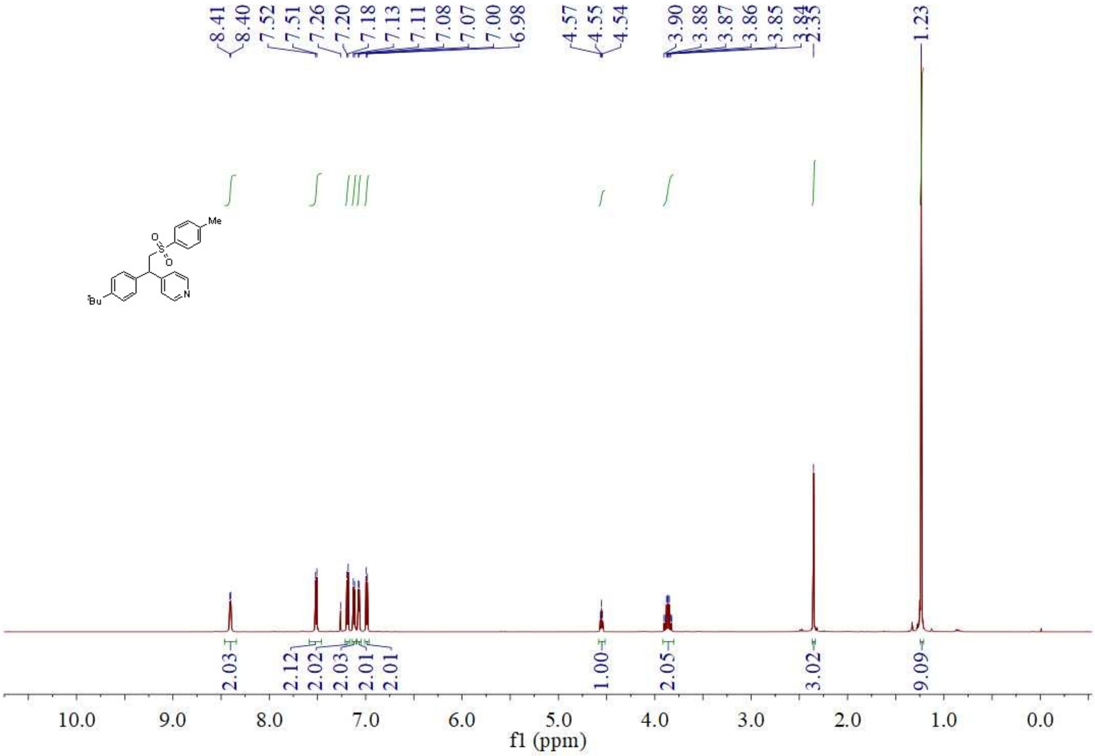

**38;** $^{13}$C NMR (101 MHz, $CDCl_3$)

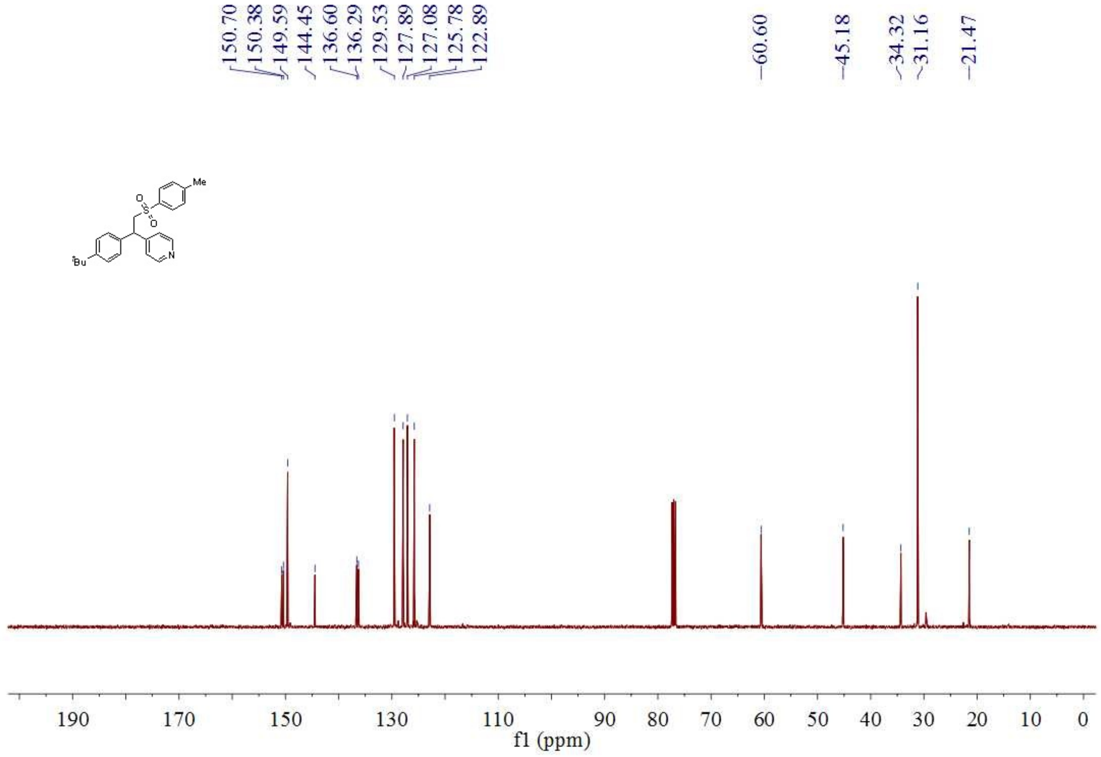


**39;** $^{1}$H NMR (400 MHz, $CDCl_3$)

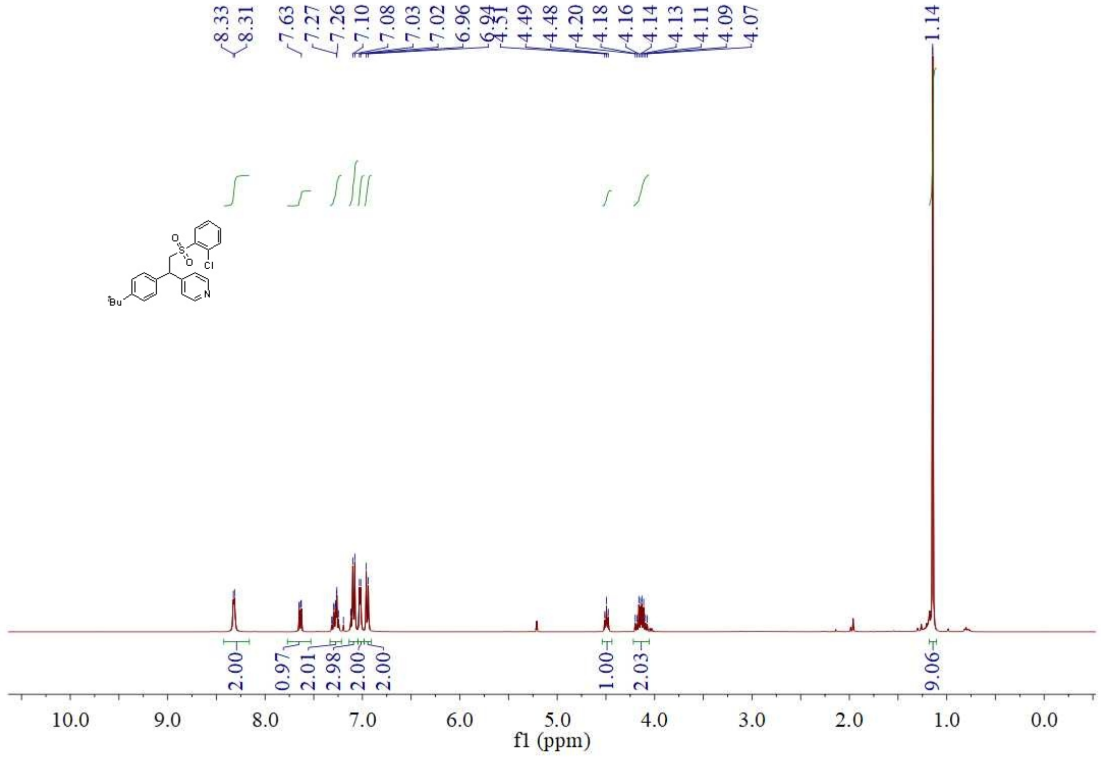

**39;** $^{13}$C NMR (101 MHz, $CDCl_3$)

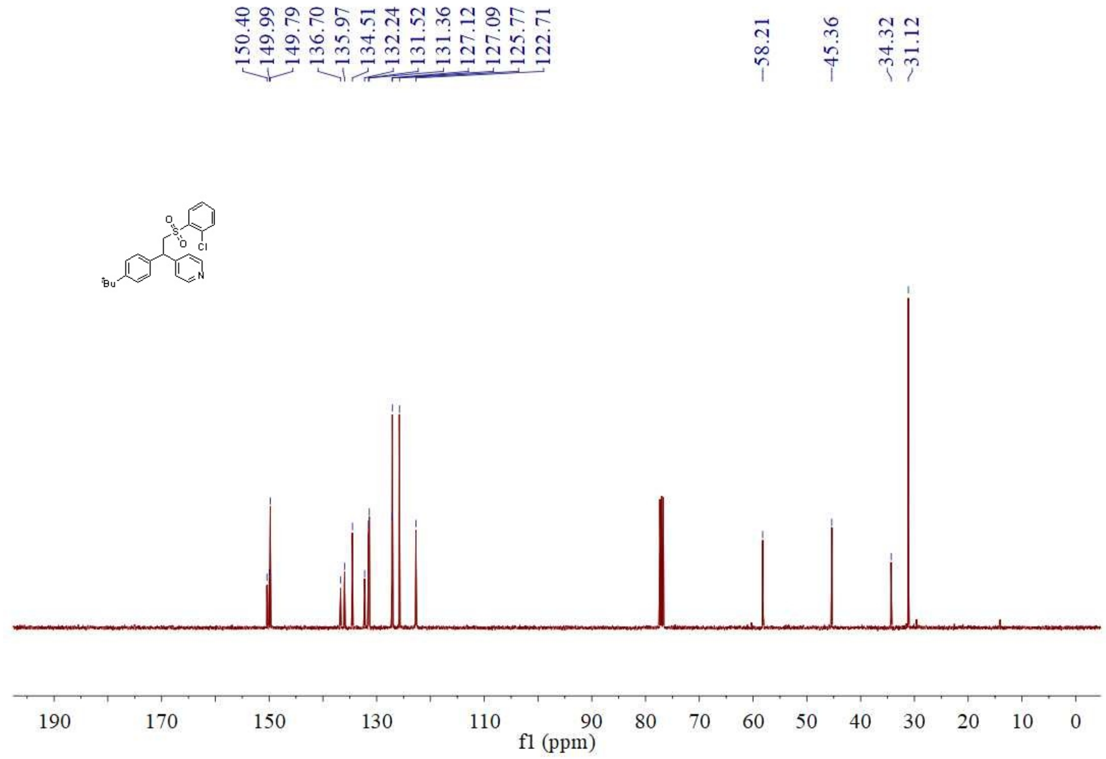


**40;** $^{1}$H NMR (400 MHz, $CDCl_3$)

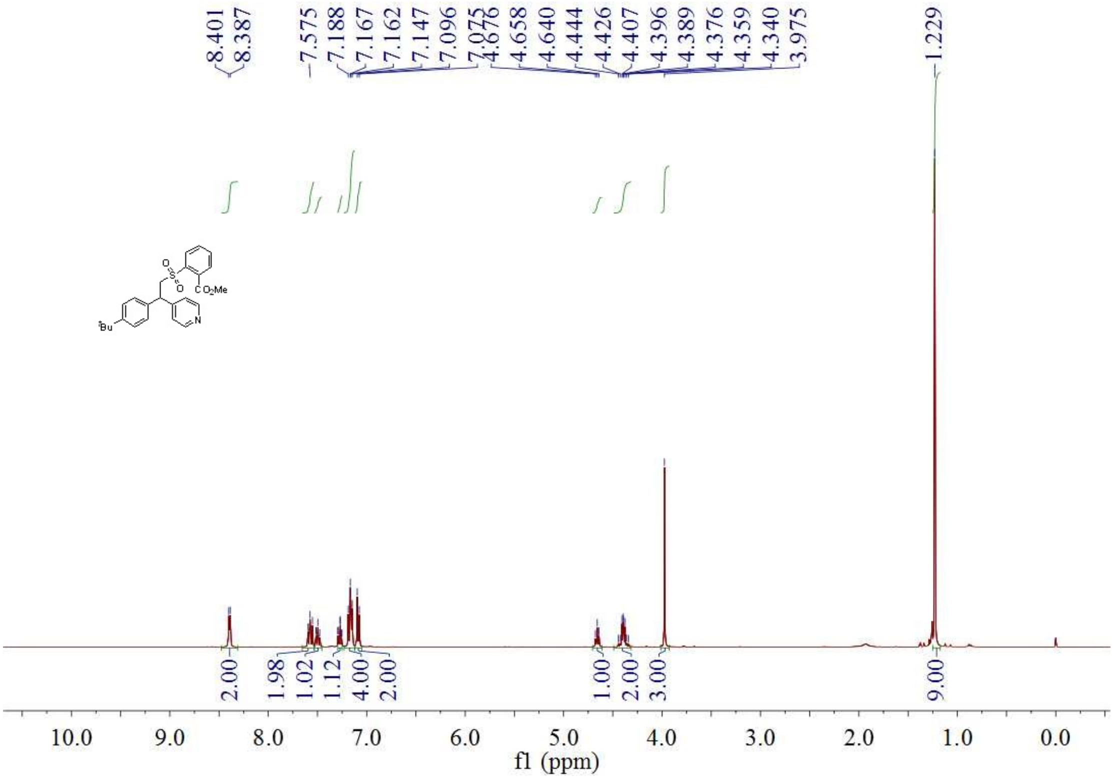

**40;** $^{13}$C NMR (101 MHz, $CDCl_3$)

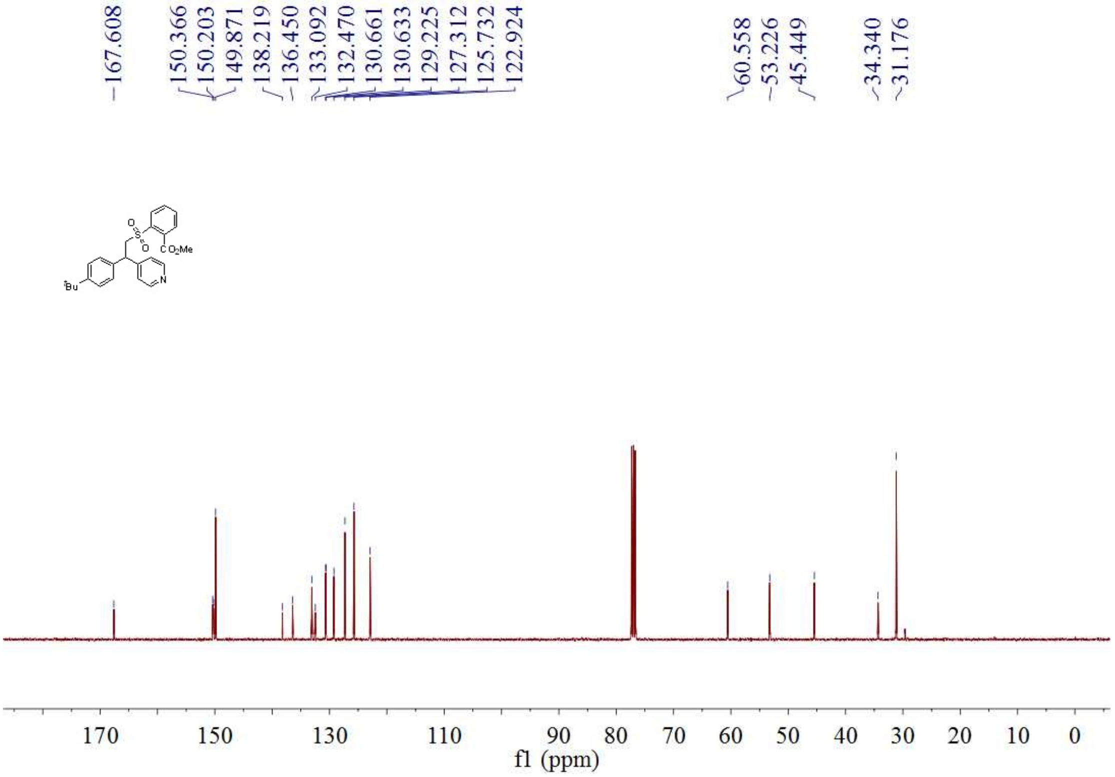


**41;** $^{1}$H NMR (400 MHz, $CDCl_3$)

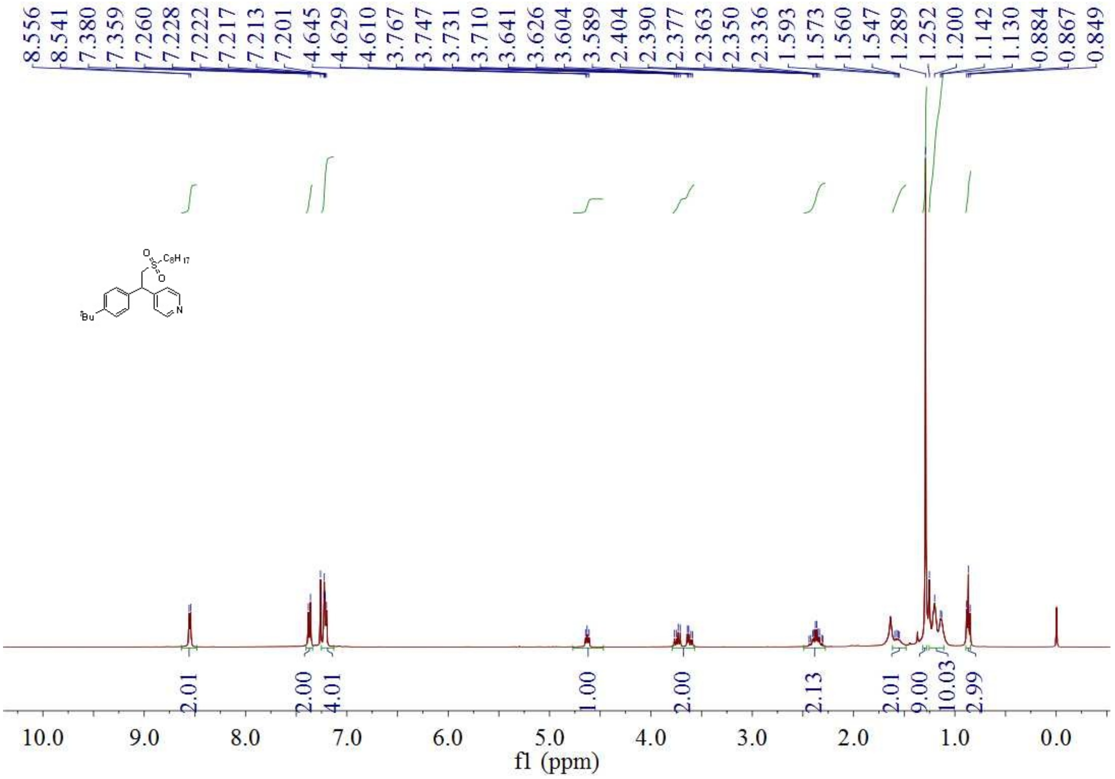

**41;** $^{13}$C NMR (101 MHz, $CDCl_3$)

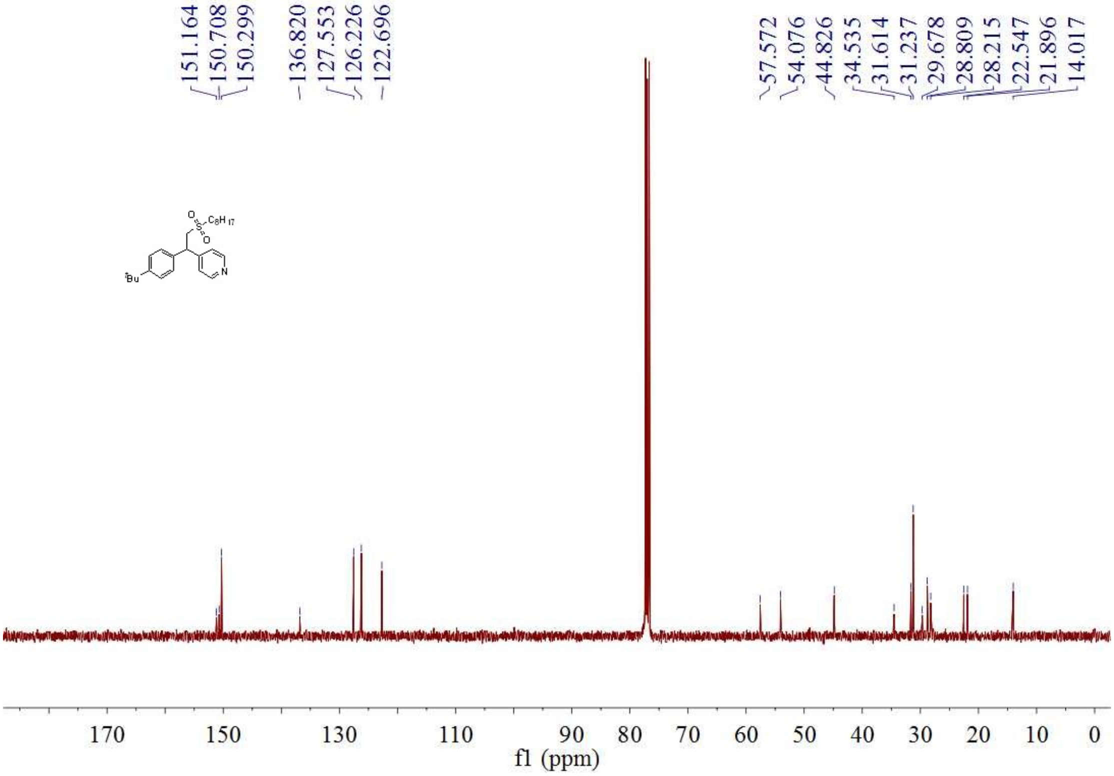


**42;** $^{1}$H NMR (600 MHz, $CDCl_3$)

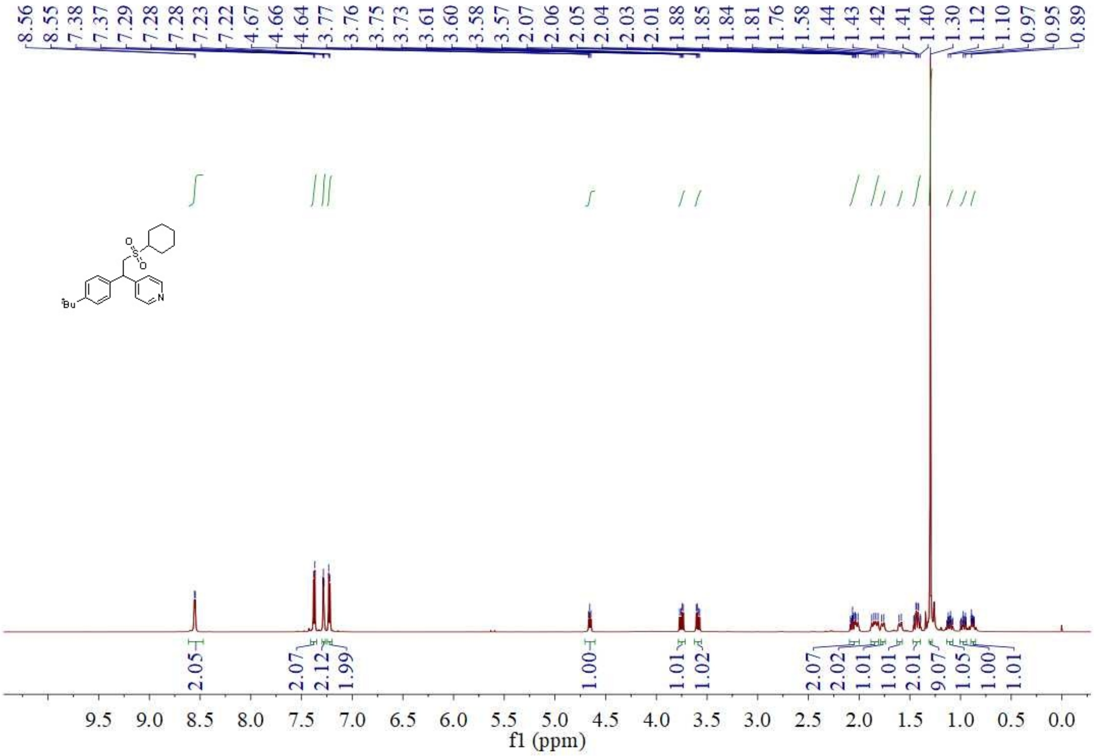

**42;** $^{13}$C NMR (101 MHz, $CDCl_3$)

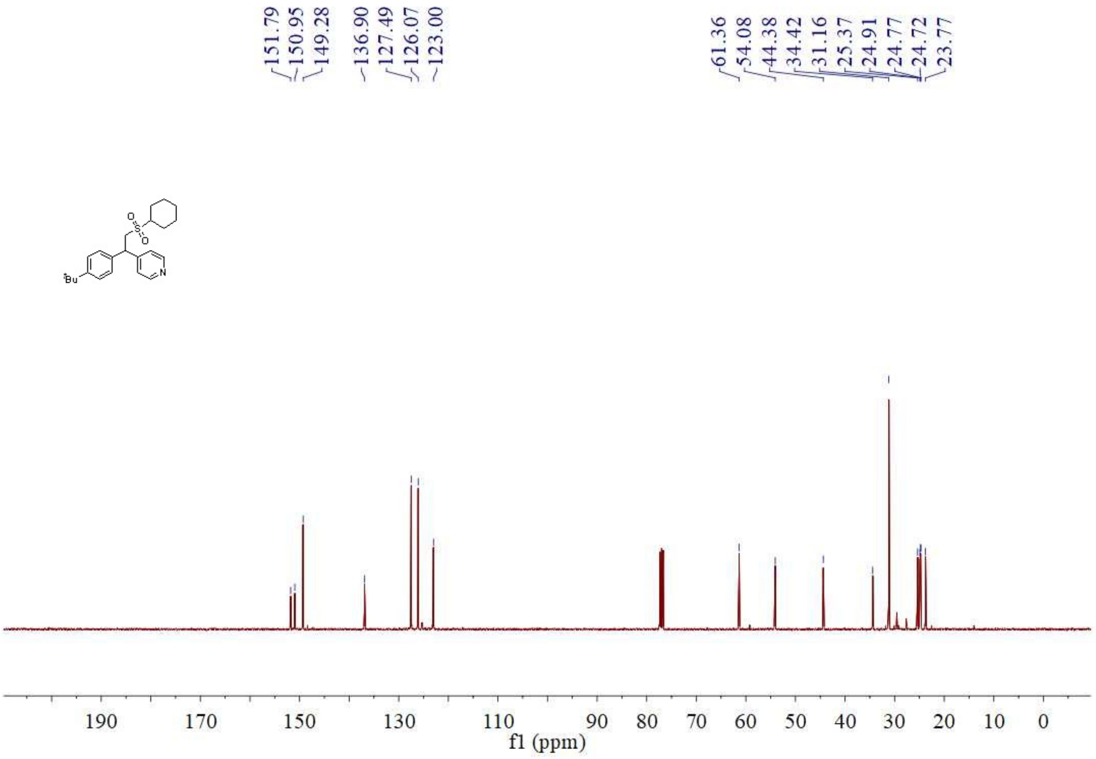


**43;** $^{1}$H NMR (400 MHz, $CDCl_3$)

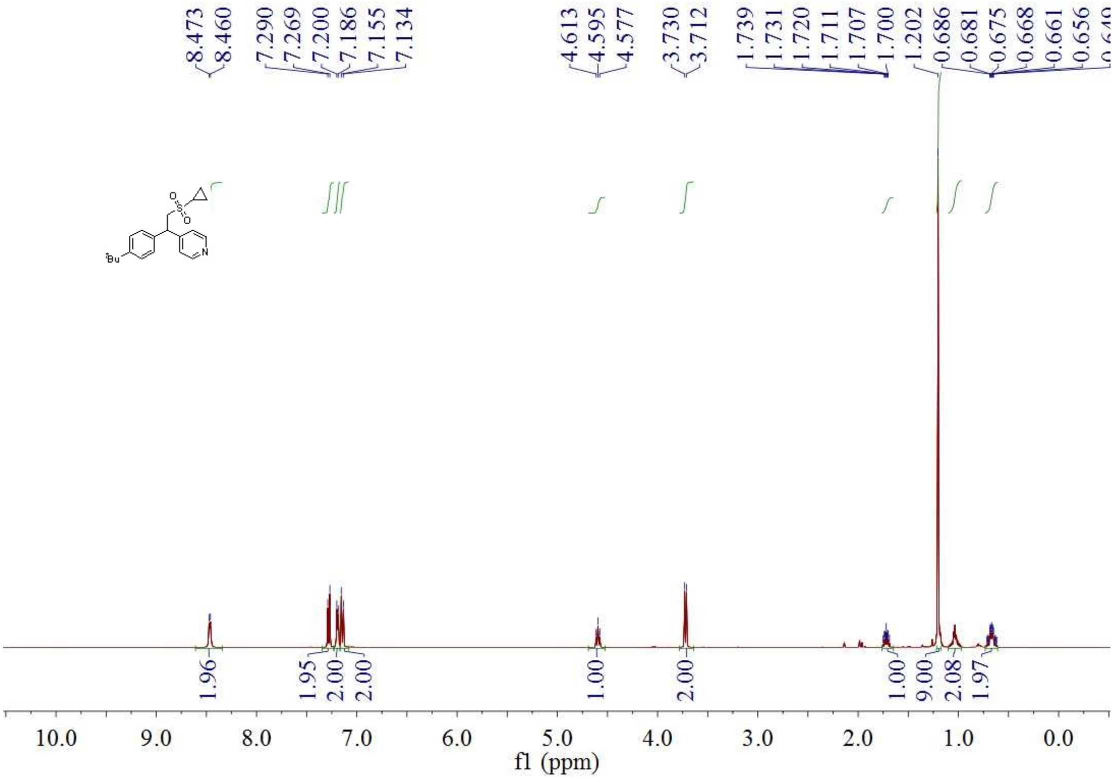

**43;** $^{13}$C NMR (101 MHz, $CDCl_3$)

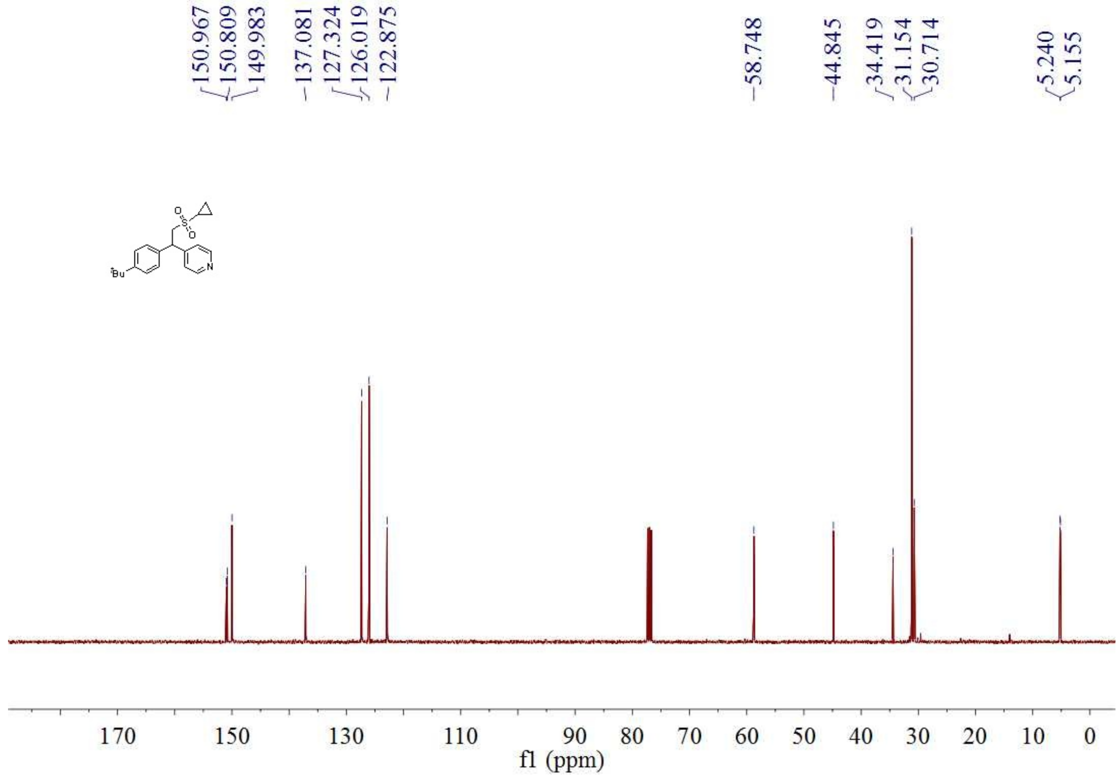


**45;** $^{1}$H NMR (400 MHz, $CDCl_3$)

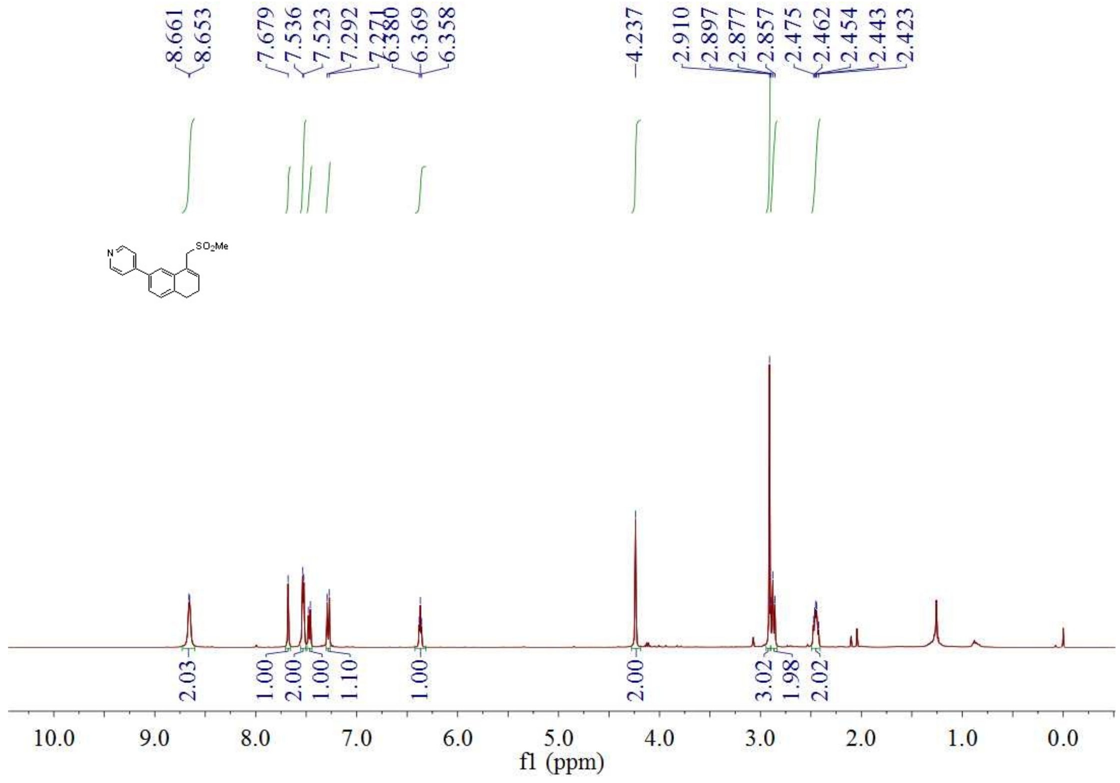

**45;** $^{13}$C NMR (101 MHz, $CDCl_3$)

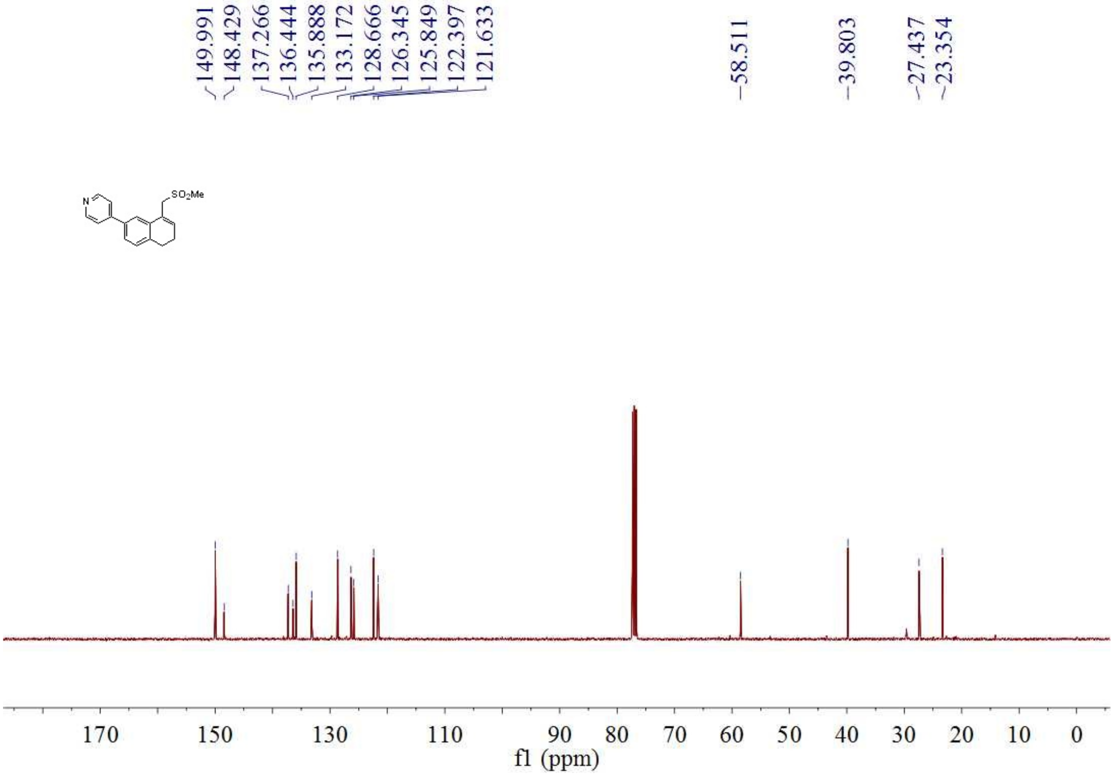


**S1;** $^{1}$H NMR (400 MHz, $CDCl_3$)

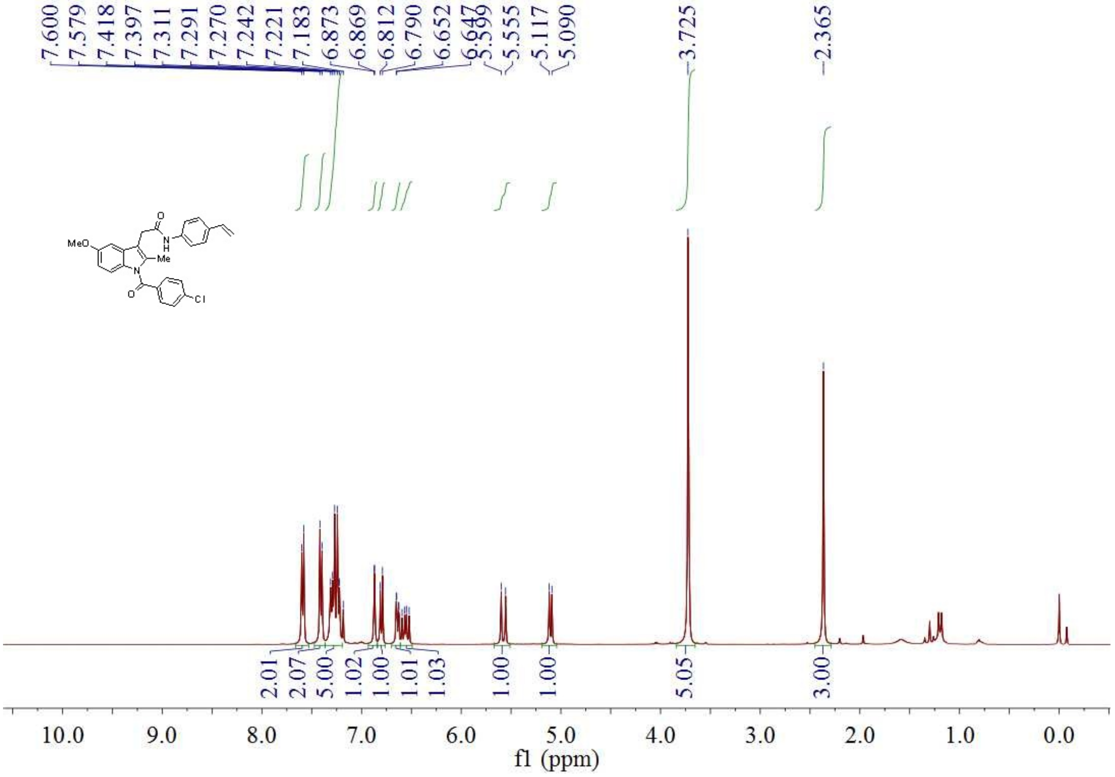

**S1;** $^{13}$C NMR (101 MHz, $CDCl_3$)

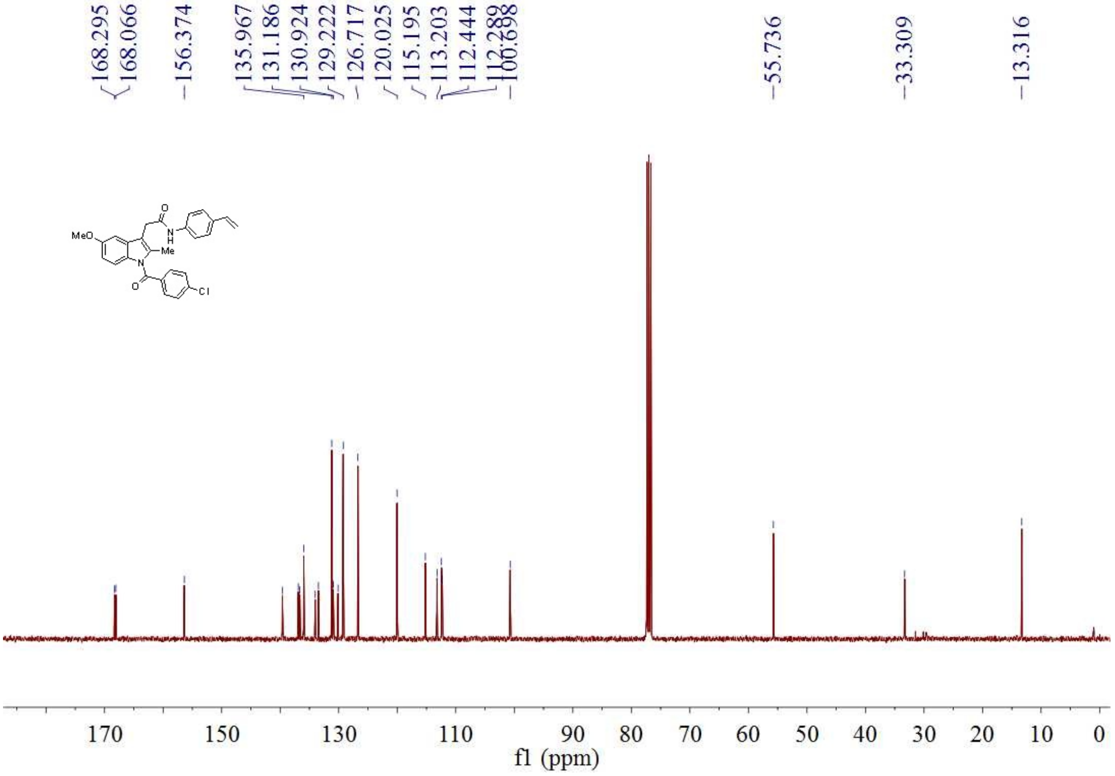

## 8. References


1. Lipp, B.; Kammer, L. M.; Kücükdisli, M.; Luque, A.; Kühlborn, J.; Pusch, S.; Matulevičiūtė, G.; Schollmeyer, D.; Šačkus, A.; Opatz, T., *Chem. Eur. J.* **2019,** *25*, 8965-8969.
2. Zhou, Y.; Bandar, J. S.; Buchwald, S. L., *J. Am. Chem. Soc.,* **2017,** *139* (24), 8126-8129.
3. Kunitski, M.; Eicke, N.; Huber, P.; Köhler, J.; Zeller, S.; Voigtsberger, J.; Schlott, N.; Henrichs, K.; Sann, H.; Trinter, F.; Schmidt, L. P. H.; Kalinin, A.; Schöffler, M. S.; Jahnke, T.; Lein, M.; Dörner, R., *Nat. Comm.* **2019,** *10*, 1.
4. Zhu, S.; Qin, J.; Wang, F.; Li, H.; Chu, L., *Nat. Comm.* **2019,** *10*, 749.
